# Supplementary material for: Does early intervention improve outcomes in the physiotherapy management of lumbar radicular syndrome? Results of the POLAR pilot randomised controlled trial
Source: BMJ Open. 2018 Jul 28;8(7):e021631. doi: 10.1136/bmjopen-2018-021631 (PMC6067394; doi:10.1136/bmjopen-2018-021631)
Supplement: Supplementary data [file bmjopen-2018-021631supp001.pdf]

POLAR

Physiotherapy for Sciatica Study

PhysiOtherapy Management of LumbAr Radicular Syndrome

Does Early Intervention Improve Outcomes

Patient Recruitment Flow

G.P sees Patient with LRS

Ineligible

Eligible

Give Patient Information Sheet

Put Date on sheet

G.P refers patient to PhysioWorks

Fax to: 0114 3051698

Eligibility Criteria

Must be & have:

1. Unilateral symptoms of **sciatica** or **femoral** radicular pain

   (See over for definitions)

Exclusion Criteria

Must **not** have:

1. 'Red flag' signs or symptoms
2. Cancer at the time of the study
3. Proven vascular claudication
4. Bilateral signs/symptoms
5. Spinal fracture within the last 3 months
6. Poor English skills (necessitating the use of an interpreter)

Characteristics of Sciatica (L4,5,S1,S2)

- Pain and/or paraesthesia and/or numbness in a L4-S2 dermatomal distribution.
- Symptoms usually buttock, posterior or lateral thigh and anterior/posterior/lateral leg.
- Shooting, burning or lancinating type pain.
- Symptoms extend below the knee.

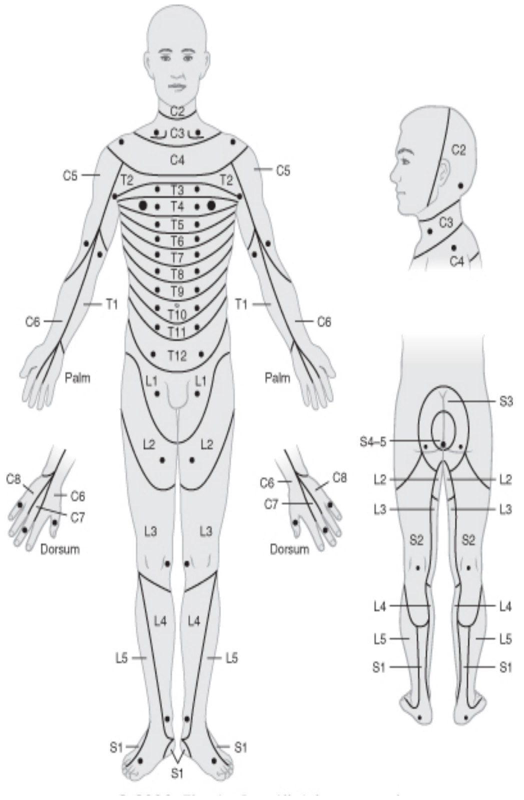

Characteristics of Femoral radicular pain (L1,2,3)

- Anterior/Antero-lateral thigh pain and/or paraesthesia and/or numbness in L1-3 dermatomal distribution.
- Shooting, burning or lancinating type pain.
- Symptoms extend above or to the knee, not below the knee.

Red Flag Signs and Symptoms

Red flag signs or symptoms are those which arose suspicion of potential serious or life-threatening pathology. There are hundreds of red flags in the literature, most of which of spurious validity. However a few useful ones in relation to low back pain and sciatica are:

- Previous cancer
- Systemically unwell (? Potential discitis)
- Spinal deformity (? fracture)
- Significant, unexplained weight loss
- Severe, unremitting pain
- I.V drug use (infection-?discitis)
- Cauda equina syndrome
- Upper motor neurone signs or symptoms (loss of dexterity, worsening balance, gait disturbance, falls, positive UMN tests-hyperreflexia, positive Romberg sign, positive Hoffman sign, muscular hypertonicity etc)

This list is of course not exhaustive and services only to highlight those patients who require further investigation rather than physiotherapy.
