# Supplementary material for: Does early intervention improve outcomes in the physiotherapy management of lumbar radicular syndrome? Results of the POLAR pilot randomised controlled trial
Source: BMJ Open. 2018 Jul 28;8(7):e021631. doi: 10.1136/bmjopen-2018-021631 (PMC6067394; doi:10.1136/bmjopen-2018-021631)
Supplement: Supplementary data [file bmjopen-2018-021631supp002.pdf]

Does early intervention improve outcomes in the physiotherapy management of Lumbar Radicular Syndrome?

Logic Model

Intervention

1. Intervention components

a. Movement restriction
b. Neurological treatment
c. Treatment of Psychological barriers
d. Education regarding aetiology/prognosis of the problem
e. Conditioning for function
f. Movement control exercise
g. Pain control
h. Patient-centred-goal orientated care

2. Referral System change

a. Faxed referral
b. Designated slots for early intervention

3. Training

a. Specialist training for participating physiotherapists in diagnosis & management of LRS
b. Referral training for G.Ps

Short-term outcomes

1. Goal Achievement

- Individual, dynamic SMART goal achievement

2. Physical Factors

- Improved movement
- Improved neurological signs &/or symptoms
- Improved conditioning for function
- Improved uni or multi-planar neuro-muscular control
- Improved pain control
- Improve meaningful function

3. Psychological factors

- Decreased psychological barriers a to successful outcome
- Improved understanding of the problem(s)
- Increase confidence in movement & function

System Change

- Available capacity for appointments
- G.Ps able to refer for physiotherapy immediately
- Improved quality of referrals

Training

- Improved specialist knowledge & skills for physiotherapists

Moderating & Mediating Factors

Patient Factors

- Acceptability of the intervention
- Fear (kinesophobia & prognosis)
- Lack of understanding
- Perception of aetiology
- Perception of prognosis
- Perceived value of physiotherapy
- Availability of parking
- Age of patient
- Degree of severity

G.P factors

- Perception of physiotherapy & service provider
- Knowledge of LRS
- Fear (litigation-CES, complaint)
- Research burden
- Research interest

Physiotherapy factors

- Training & support

System-related factors

- Booking POLAR patients into correct appointments slots
- POLAR slot availability

Outcomes

Physiotherapists

- Job satisfaction
- Increased knowledge
- Increased confidence in treating patients with LRS

Patient

- Return to function
- Satisfaction with service
- Time to treatment

Potential Impact

Referral pathway

Service delivery efficiency

Cost effectiveness
