# Supplementary material for: Does early intervention improve outcomes in the physiotherapy management of lumbar radicular syndrome? Results of the POLAR pilot randomised controlled trial
Source: BMJ Open. 2018 Jul 28;8(7):e021631. doi: 10.1136/bmjopen-2018-021631 (PMC6067394; doi:10.1136/bmjopen-2018-021631)
Supplement: Author's manuscript [file bmjopen-2018-021631.draft_revisions.pdf]

BMJ Open is committed to open peer review. As part of this commitment we make the peer review history of every article we publish publicly available.

When an article is published we post the peer reviewers' comments and the authors' responses online. We also post the versions of the paper that were used during peer review. These are the versions that the peer review comments apply to.

The versions of the paper that follow are the versions that were submitted during the peer review process. They are not the versions of record or the final published versions. They should not be cited or distributed as the published version of this manuscript.

BMJ Open is an open access journal and the full, final, typeset and author-corrected version of record of the manuscript is available on our site with no access controls, subscription charges or pay-per-view fees (http://bmjopen.bmj.com).

If you have any questions on BMJ Open's open peer review process please email info.bmjopen@bmj.com

BMJ Open

Does early intervention improve outcomes in the physiotherapy management of Lumbar Radicular Syndrome? Results of the POLAR Pilot Randomised Controlled Trial

Journal:	*BMJ Open*
Manuscript ID	bmjopen-2018-021631
Article Type:	Research
Date Submitted by the Author:	15-Jan-2018

Complete List of Authors:	Reddington, Michael; Sheffield Teaching Hospitals NHS Foundation Trust, Therapy Services<br>Walters, Stephen; University of Sheffield, ScHARR<br>Cohen, Judith; Hull York Medical School, Clinical Trials Research Unit<br>Baxter, Susan; University of Sheffield, ScHARR<br>Cole, Ashley; Sheffield Teaching Hospitals NHS Foundation Trust, Orthopaedic

Keywords:	Back pain < ORTHOPAEDIC & TRAUMA SURGERY, Musculoskeletal disorders < ORTHOPAEDIC & TRAUMA SURGERY, Spine < ORTHOPAEDIC & TRAUMA SURGERY

SCHOLARONE™
Manuscripts

Does early intervention improve outcomes in the physiotherapy management of Lumbar Radicular Syndrome? Results of the POLAR Pilot Randomised Controlled Trial

Corresponding author:

Michael Reddington
Therapy Services Outpatient Department
Sheffield Teaching Hospitals NHS Foundation Trust
Northern General Hospital
Herries Road
Sheffield S5 7AU
MReddington1@sheffield.ac.uk

Professor Stephen J Walters
Designs, Trials and Statistics
ScHARR, University of Sheffield
30 Regents Court
Sheffield S1 4DA
S.J.Walters@sheffield.ac.uk

Dr Judith Cohen
Hull Health Trials Unit
Hull York Medical School, University of Hull
Allam Medical Building, Cottingham Road
Hull HU6 7RX
J.Cohen@hull.ac.uk

Dr Susan Baxter
Section of Public Health
ScHARR, University of Sheffield
30 Regents Court
Sheffield S1 4DA
s.k.baxter@sheffield.ac.uk

Mr Ashley Cole
Consultant Orthopaedic Spinal Surgeon
Sheffield Teaching Hospitals NHS Foundation Trust
Northern General Hospital
Herries Road
Sheffield S5 7AU
Ashley.Cole@sth.nhs.uk

For peer review only

For peer review only - http://bmjopen.bmj.com/site/about/guidelines.xhtml

Abstract

Objective

To investigate the feasibility of undertaking a definitive RCT.

Setting

This was a pilot, pragmatic superiority Randomised Controlled Trial (RCT) with a qualitative element, recruiting from 14 General Practitioner (G.P) practices in England.

Participants

Patients over 18 presenting to their G.P with unilateral LRS were eligible to participate in the study, those who did not have a clear understanding of the English language or had co-morbidities preventing rehabilitation were ineligible.

Interventions

Participants were randomised into early intervention physiotherapy or usual care with both groups receiving a patient-centred, goal orientated physiotherapy programme specific to their needs. Participants received up to 6 treatment sessions over an 8-week period.

Outcome measures

Process outcomes to determine the feasibility of the study and an exploratory analysis of patient reported outcomes including self-rated disability, pain and general health, these were collected at baseline, 6, 12 and 26 weeks post randomisation.

Results

80 participants were recruited in 10 G.P practices (early intervention physiotherapy n= 42, usual care n=38). No evidence of selection bias was observed in the study. Follow-up rates at 26 weeks were 36 (86%) in the early intervention physiotherapy group and 32 (84%) in the usual care. Recruitment lasted 34 weeks. All feasibility objectives were met.

There was 1 serious adverse event observed in a participant in the early intervention physiotherapy group which was not associated with the intervention.

Conclusions

The results of the study suggest a full RCT is feasible and will provide evidence as to the optimal timing of physiotherapy for patients with LRS.

Trial Registration number ISRCTN: 25018352

For peer review only - http://bmjopen.bmj.com/site/about/guidelines.xhtml

Ethics & Dissemination

The study received favourable ethical review from the East of Scotland Research Ethics Service (EoSRES) on the 20th August 2015 (15/ES/0130). Recruitment began on the 1st March 2016 and closed in November 2016.

Strengths and limitations of the study

- This pilot RCT was conducted in the usual care setting with clinical staff delivering the intervention.
- All feasibility objectives were met, including recruitment and participant attrition, and so the study can directly inform the design and conduct of a definitive RCT.
- Participants self-referred into the study after an introduction from their G.P (a pre-requisite for ethics approval) and so this group of patients may not be representative of a wider population.
- This was a pilot RCT and as such all analyses are exploratory.

Protocol

The protocol for the POLAR study was published and can be accessed at: http://bmjopen.bmj.com/content/bmjopen/7/3/e014422.full.pdf

For peer review only

For peer review only - http://bmjopen.bmj.com/site/about/guidelines.xhtml

Introduction

Lumbar Radicular Syndrome (LRS) is a painful and disabling condition, usually of benign causation and in around 90% of cases associated with an intervertebral disc prolapse [1]. It can be self-limiting, lasting a short time with no significant sequelae or can be a major cause of prolonged disability, work loss and long-term presentation to healthcare with associated costs [2,3]. Lifetime prevalence is estimated to be between 1% and 43% [4] and an annual incidence of between 1% and 5% [5].

Spontaneous resorption of the Inter Vertebral Disc (IVD) prolapse and with it symptom resolution does occur, and it has been suggested that by 12 weeks 75% of LRS sufferers will have symptomatically resolved [6]. However, there is no reliable predictor of early, late or no recovery at all. Treatment guidelines encourage conservative management in the first instance before considering surgery.

Surgery for patients with LRS has been advocated, with good reported outcomes. The optimum timing for surgery appears to be between 4 weeks and 6 months after symptom onset [7–10]. However, a significant number of patients never have any substantial relief from surgery with unsatisfactory outcomes in over 20% of patients at 5 years [11–13].

Physiotherapy for LRS is commonly employed in the United Kingdom for the management of LRS however, there is a lack of consensus on the type, duration and timing of the physiotherapy intervention [14]. It is known that patients prefer and have improved outcomes with early intervention physiotherapy for Low Back Pain (LBP) [15–17] and that delayed initiation of physiotherapy for LRS increases healthcare consumption [18]. Patients who received physiotherapy less than 4 weeks after onset of their LBP had lower healthcare usage and associated costs than those who received physiotherapy more than 3 months after symptom onset [19]. This suggests early treatment is important therefore in terms of cost-savings and prevention of chronic symptom development [20] as increased symptom duration leads to worse outcomes for patients who undertake conservative or surgical care [21]. The decision about when to send patients with LRS for physiotherapy is difficult, too early and you may be treating patients who would enjoy spontaneous resolution, too late and the optimum window of treatment including surgery, may have closed.

For peer review only - http://bmjopen.bmj.com/site/about/guidelines.xhtml

For peer review only

Aims and Objectives

The study aim was to investigate the feasibility of undertaking a full Randomised Controlled Trial (RCT) to determine the effectiveness and cost-effectiveness of early intervention physiotherapy for patients with LRS.

Process Objectives

1. Successfully set-up recruitment sites in G.P practices.
2. Achieve a recruitment rate of 7 participants per month.
3. Demonstrate the ability to organise 75% of physiotherapy appointments within 2 weeks of randomisation.
4. Provide an appointment within 20 days of randomisation for >75% of participants randomised to the intervention group.
5. Achieve a participant attendance at >66% of physiotherapy appointments.
6. Achieve a participant attrition rate of <25% over the course of the study.
7. Achieve 80% return of Patient Reported Outcome Measures (PROMS) at 6/52 follow-up.

Research objectives

1. To test the feasibility, practicality, safety and acceptability of the study design and protocol.

2. Demonstrate acceptability of the primary and secondary outcome measures to patients and clinicians.

3. To inform the sample size calculation for the definitive RCT trial.

Methods

Design and setting

This was a mixed methods study comprising of an external pilot RCT with an embedded qualitative component in the form of stakeholder interviews in 14 G.P practices in a large city in England. Known as the POLAR study, the pilot RCT will be presented in this paper. A change was made to the inclusion criteria after 1 week of recruitment, the upper age limit of 70 was removed as this excluded a number of potential participants. The protocol for the study has been published, including extensive details of methods [22].

For peer review only - http://bmjopen.bmj.com/site/about/guidelines.xhtml

Randomisation

Information from the baseline dataset was used to randomise the participants using a web-based system. The Oswestry Disability Index (ODI) [23] was used as the stratification factor with 3 levels based on ODI severity; ‘mild & moderate’ (≤22-40%), ‘severe’ (>40 to 60%) and ‘crippled’ (>60 to 80%). A blinded block size was used to minimise predictability. The random allocation sequence and block size, stratified by centre and ODI disability score was independently generated by the Sheffield Clinical Trials Research Unit (CTRU).

Participants were informed of their group allocation within 1 working day of their consent and randomisation. Participants were randomised to treatment at either 2 or 6 weeks post-randomisation, we were unable to blind either patients or clinicians to the treatment allocation as it was obvious at what time-point they were receiving treatment. In an effort to minimise bias, both groups of patients received protocolised treatment based on the same assessment and treatment framework at the different time points.

Participants

Potential participants were identified by their G.P and given details of the study. If interested, they contacted a member of the research team who screened for eligibility and arranged to meet to discuss the study. Anyone over the age of 18 years with unilateral LRS and who could speak English were eligible. If they had ‘red flag’ signs or symptoms such as cancer, cauda equine syndrome, spinal fracture or had other physical or psychological disabilities preventing rehabilitation, they were ineligible.

Recruitment & consent

Written consent was obtained by the research team after meeting the potential participant. There were three recruitment cycles, each lasting up to 12 weeks or until 27 participants had been recruited for that cycle (26 for the final cycle). The remaining eight weeks were used for completion of treatment. A two-week period between cycles provided time to reflect and analyse the results from the stakeholder interviews and other feedback to refine the study processes as necessary.

For peer review only - http://bmjopen.bmj.com/site/about/guidelines.xhtml

The Intervention

The intervention was protocolised and allowed the treating physiotherapist a range of treatment options within each domain with the selected options recorded electronically for each treatment session. The goal-orientated physiotherapy regimen for both groups were tailored to the individuals' requirements based on the information gathered from the baseline interview data, PROMS and clinical assessment. Participants were assessed using a multi-dimensional approach based on seven different elements; psychological barriers to recovery, neurological factors, movement restriction, understanding, conditioning, movement control and pain. Individualised physiotherapy for LBP and LRS is known to be superior and more cost-effective than advice alone [24,25], it is flexible and directly relevant to the individual and their changing needs. Participants received a maximum of six sessions of physiotherapy over an eight-week period, fewer if their pre-determined goals had been achieved. A logic model has been developed for the intervention which can be found in the supplementary material.

Treatment Fidelity

Several strategies were employed to optimise fidelity, including a protocolised training package for the treating physiotherapists, standardised patient information, weekly feedback and support of treating physiotherapists and video analysis of each participating physiotherapist treating a study participant. The study took place in an NHS community setting using three physiotherapists, already employed by the host service provider. The physiotherapists had a mean age of 36 years (range 34-40 years) and a mean of 10 years postgraduate experience (range 7-12 years). They underwent 21 hours of training in the assessment and intervention and to promote and facilitate self-management, optimal function, pacing advice, analgesic advice together with equipping the patient with coping strategies.

Outcomes

Patients were asked to complete self-report and screening measures by post or face to face at four time points: firstly, at the time of consent and then at 6, 12 and 26 weeks post randomisation**.** The primary outcomes for the study were process outcomes as the objective was to determine the feasibility of carrying out a full-scale RCT. Secondary outcomes were the ODI, Visual Analogue Scale

For peer review only - http://bmjopen.bmj.com/site/about/guidelines.xhtml

(VAS) for back and leg pain, Keele STarT Back score [26], EQ5D-5L [27] and a self-report form focussing on functional loss, goals and medical history.

Sample size

It has been recommended that an external pilot study should have at least 70 measured participants (35 per group) when estimating the standard deviation for a continuous outcome [28]. A sample size of 80 patients, with approximately 10% allowance for loss to follow-up allows the standard deviation of an outcome to be estimated to within a precision of approximately ±16% of its true underlying value with 95% confidence.

Results

The flow chart of the participant journey for the POLAR study can be viewed in Figure 1. Ninety potential participants who were given details of the study by their respective G.Ps contacted the research team. Ten were excluded as they either did not meet the inclusion criteria or refused to be randomised, with 80 going on to be randomised from 10 different primary care G.P practices.

Baseline characteristics

The baseline characteristics of all participants, by group can be found in Table 1. This illustrates the comparability of the 2 arms with no evidence of selection bias. The groups were well matched for demographic factors such as age, gender and BMI as well as levels of disability, pain in leg and back, risk of chronicity and general health status. The early intervention physiotherapy group had longer symptom duration going into the study.

For peer review only

Table 1 Baseline characteristics of POLAR participants

	Early Intervention physiotherapy			Usual Care			Total		
	N	Mean	SD	N	Mean	SD	N	Mean	SD
**Age** (years)	42	47	14	38	47	13	80	47	13
**Female**	21			18			39		
**White British**	38			33			71		
**Height** (CM)	42	172.1	10.7	38	172.1	9.8	80	171.7	10.2
**Weight** (KG)	39[1]	81.5	14.8	38	80.6	15.7	77	81	15.2
**BMI**	39[1]	27.7	4.6	38	27.3	5.6	77	27.5	5.1
**ODI score** (%)	42	44.6	19.5	38	45.2	17.4	80	44.9	18.4
**Leg Pain**	42	7.2	1.8	38	6.9	2.3	80	7	2.1
**Back pain**	42	5.4	3.3	38	6	2.6	80	5.7	3.0
**EQ5D-5L** VAS	42	63.8	20.6	38	64.6	18.9	80	64.1	19.7
**EQ5D-5L** Utility score	42	0.44	0.29	38	0.52	0.25	80	0.48	0.27
**Keele StartBack**	42	5.7	2.0	38	5.7	1.8	80	5.7	1.9
**Keele Startback** Sub-score	42	2.0	1.5	38	2.7	1.3	80	2.8	1.4
**Symptoms duration** (days)	42	92	276	38	61	51	80	77	203
**Time to treatment** (days) [2]	38	11.1	10.5	31	43.6	8.9	69	25.7	19.0
**Number of physiotherapy sessions**	38	4	1	31	3	2	69	4	2

[1] 3 missing values

[2] Time between randomisation and first scheduled treatment session

Process Results

The POLAR study is a pilot trial and outlined below are the results of the feasibility objectives.

Set-up of recruitment sites in primary care

Twenty G.P practices were initially approached to take part in the study, with ten agreeing to participate. Towards the end of the second tranche of recruitment it was evident that one practice was recruiting a large number of participants and a decision was made to enrol new recruitment centres. Seven further G.P practices were therefore approached, with four agreeing to participate.

Recruitment rate

Eighty participants were recruited between the period 1st March 2016 and 7th November 2016 with a recruitment rate of 2.4 participants per week or 9.6 participants per month which enabled recruitment to end earlier than anticipated. Forty-two participants were randomised into the early intervention group and 38 in the usual care group.

For peer review only

For peer review only - http://bmjopen.bmj.com/site/about/guidelines.xhtml

Organisation of physiotherapy appointments

The target of 75% of physiotherapy appointments being made within two weeks of randomisation was surpassed in both groups. 100% (42/42) (95% CI: 92% to 100%) of early intervention physiotherapy participants received their appointment within 20 days of randomisation and 38/38 (95% CI:91% to 100%) in the usual care group. This illustrates the feasibility of making appointments for participants at short notice.

The feasibility of intervention delivery

A key feasibility parameter was the ability for at least 75% of early intervention physiotherapy participants to be seen by a physiotherapist, within 20 days of randomisation. 100% (42/42) (95% CI 92% to 100%) of participants reached this target, with a mean of 14.1 days between randomisation and first treatment session.

Participant treatment session attendance

The mean attendance rate for physiotherapy appointments in both groups was 92.6% (SD 16.2), 93.8% (SD 12.6) for the intervention group physiotherapy and 91.1% (SD 19.8) in the usual care group. All surpassed the a priori target of greater than 66% attendance.

Participant attrition

Eighty participants agreed to take part in the study. The intervention group attrition rate was 14% (6/42) (95% CI: 7% to 28%) and in the usual care group it was 16% (6/38) (95% CI 7% to 30%) at 26 weeks follow-up. The overall attrition rate for drop out of participants was 15% (95% CI 9% to 24%), all within the a priori limit set at 25%.

Outcome measure return

The outcome measure return rates surpassed expectations of 80% at six weeks and were as follows: 38/42 (91%; 95% CI: 78% to 96%) at six weeks post randomisation for the intervention group and 35/38 (92%; 95% CI 79% to 97%) for the usual care group.

Research results

Analysis of key clinical outcomes

For peer review only - http://bmjopen.bmj.com/site/about/guidelines.xhtml

Figure 2 shows the leg pain and ODI scores (likely primary outcome measures for definitive RCT) for participants with all 4 assessments completed. The blue line illustrates the increased rate of recovery in the early intervention physiotherapy group up to 6 weeks. When the usual care group begins their physiotherapy the rate of recovery assimilates and by 12 weeks and both groups have very similar scores.

The feasibility, practicality, safety and acceptability of the study design and protocol

The feasibility of the study has been suggested by the results of the feasibility parameters. There were several adjustments made to the processes of the study which were made possible by the breaks in recruitment. These included a brief weekly email to all participating G.Ps to remind them of the study and improve the clarity of inclusion and exclusion criteria. A change to the process of administering the six-week outcome measures was necessary, after the physiotherapists reported it too time consuming to administer. There were no changes made to the intervention, which appeared to be well received by both participants and clinicians alike. There were no adverse events or serious adverse events associated with the intervention or the study processes.

Harms

There was one Serious Adverse Event (SAE) during the course of the study in the early intervention physiotherapy group. The SAE rate was 2% (1/42) in the early intervention physiotherapy group and 0% (0/38) in the usual care group a difference of 2% (95% CI -7% to 12%). The participant was hospitalised after suffering a Cerebro-Vascular-Accident (CVA) related to pre-existing vascular hypertension. The participant had completed their physiotherapy intervention two weeks prior and made a full recovery at 6 months. This was reported to the ethics committee and Trial Management Group (TMG).

Acceptability of the primary and secondary outcome measures to patients and clinicians

The importance of examining acceptability of the outcome measures, processes and the intervention was a key area of investigation for the study, and the pilot trial included a qualitative element to explore these aspects. Details of the qualitative aspects of the study will be reported in forthcoming

For peer review only - http://bmjopen.bmj.com/site/about/guidelines.xhtml

papers. However, in summary the key processes necessary for implementation and evaluation of the study were reported to be acceptable by all stakeholders.

Fidelity

Physiotherapists recorded the components of their treatment sessions at each patient encounter in order to enhance and measure treatment fidelity. Participants in the early intervention physiotherapy group had a mean of 4 treatment sessions and those participants in the usual care group 3 sessions. There were 269 physiotherapy sessions carried out as part of the POLAR study with 1267 component parts (Table 2), 36 (3%) of which outside the protocolled treatment framework. The components outside the protocol consisted of three sessions of acupuncture and exercise other than that in the protocol. Video analysis was carried out independently on a purposive sample of 5 treatment sessions using a fidelity assessment tool developed by the lead author, clinical colleagues and PPIE representatives. The maximum score for 'essential' aspects of fidelity was 15/15. The median score for the videos was 14/15 (93%) with a range of 13-15 (87-100%).

Sample size calculation for the definitive RCT trial

For the definitive RCT we propose the primary outcome is the ODI at 26-weeks post-randomisation as the ODI has shown to be acceptable to patients and a commonly used measurement of self-rated disability. In this pilot trial, we observed a difference in means of 2.5 points (95% CU: -4.5 to 9.1) between the randomised groups. There is a lack of consensus regarding the Minimum Clinically Important Difference (MCID) for the ODI, with suggestions ranging from 6% to 30% [29,30]. If we assume a target difference of five-points on the ODI scale then with 217 patients per group (434 in total) we would have 90% power to detect a five-point difference or more (equivalent to standardised effect size of 0.31) between the randomised groups which would be statistically significant at the 5% two-sided level. Allowing for a conservative estimate of 20% attrition (we observed 15% in this pilot) we would need to recruit and randomise 272 per group (544 in total).

Based on the recruitment rates observed in this trial of 80 patients in 8.5 months of recruitment at 10 centres (a rate of 0.9 patients per centre month); the main trial would need around 24 centres recruiting for 24 months to achieve this target.

The descriptive statistics for all participants by group and time point can be found in Table 3.

For peer review only - http://bmjopen.bmj.com/site/about/guidelines.xhtml

Table 2 Intervention domains and components frequency table

Domain		Treatment options	Frequency	%
**Psychological barriers to recovery** [31–33]	Keele STarTBack Clinical interview & history	a. Treatment of Kinesiophobia with graded exposure, education and movement re-education	16	1.3
		b. Treatment of hypervigilance with education, distraction & desensitisation	17	1.4
		c. Treatment of faulty beliefs about pain, LRS, treatment and/or prognosis with education and self-management strategies	38	3.2
		d. Treatment of Iatrogenic beliefs and corresponding avoidance behaviours with education and movement re-education	3	0.2
		e. Treatment of aspects of work as a barrier to recovery and treatment with ergonomic advice and practise	15	1.2
		f. Identification of financial barriers to recovery and signposting e.g. debt management	15	1.2
		g. Identification of emotional barriers to recovery and signposting to appropriate therapy e.g. G.P/Psychology	57	4.7
**Neurological** [34–37]	Clinical assessment	a. Neural interface mobilisation	98	8.1
		b. Functional neurological movement re-education	7	0.6
**Movement restriction** [38,39]	Clinical assessment	a. Flexion mobilisation (Grade 2-4)	68	5.6
		b. Side-flexion mobilisation (Gr. 2-4)	5	0.4
		c. Extension mobilisation (Gr. 2-4)	15	1.2
		d. Rotation mobilisation (Gr. 2-4)	41	3.4
		e. Flexion+Side-flexion mobilisation (Gr. 2-4)	11	0.9
		f. Flexion+Side+flexion+rotation mobilisation (Gr. 2-4)	62	5.2
		g. Extension+Side flexion mobilisation (Grade 2-4)	0	0
		h. Manipulation (Gr. 5)	0	0
		i. Seated Mobilisation With Movement (MWM)	16	1.3
		j. Standing MWM	16	1.3
		k. Mobilisation into functional position	14	1.2
		l. Muscle stretches	61	5.1
		a. Pacing behaviours	53	4.4
		b. Goal attainment	58	4.8
		c. Health Promotion	80	6.6
		d. Identification and treatment of central sensitisation-liaison with G.P/pain clinic	8	0.7

For peer review only - http://bmjopen.bmj.com/site/about/guidelines.xhtml

		e. Identification and treatment of peripheral sensitisation-liaison with G.P/pain clinic	7	0.6
**Conditioning** [40,41]	Self-assessment answers, clinical interview & history	a. Cardiovascular & conditioning exercise relevant to patients' goals	83	6.9
		b. Function specific stretches	39	3.2
		c. Function specific strengthening	62	5.2
		d. Ergonomic advice	14	1.2
		e. Ergonomic practise	6	0.5
		f. Group exercise	0	0.0
		g. Perturbation training	7	0.6
**Movement control** [42]	Clinical assessment	a. Sagittal plane control in functional positions relevant to patients' problems/goals	24	2.0
		b. Coronal plane control in functional positions relevant to patients' problems/goals	15	1.2
		c. Axial plane control in functional positions relevant to patients' problems/goals	1	0.1
		d. Multi-planar control in functional positions relevant to patients' problems/goals	6	0.5
		e. Movement re-education in functional positions relevant to patients' problems/goals	18	1.5
**Pain** [43–45]	ODI<br>VAS back & leg<br>Clinical interview & history	a. Analgesic review & advice in liaison with G.P/Pharmacist	23	1.9
		b. Pain education	60	5.0
		c. Pain coping strategies	20	1.7
		d. Fear reduction intervention in liaison with psychologist/pain clinic	12	1.0
		e. Stress reduction intervention in liaison with psychologist/pain clinic	32	2.7
**Totals**			1267	99.8%[1]

[1] 0.2% missing data-2 treatment episodes where components not attributed.

For peer review only - http://bmjopen.bmj.com/site/about/guidelines.xhtml

Table 3 Descriptive statistics for outcome measures at each time point

Outcome	Baseline		6 Weeks		12 Weeks		26 weeks		AUC				
	Usual Care *n=38*	Early Intervention physiotherapy *n=42*	Usual Care *n=35*	Early Intervention physiotherapy *n=38*	Usual Care *n=32*	Early Intervention physiotherapy *n=36*	Usual Care *n=32*	Early Intervention physiotherapy *n=36*	Usual Care *n=32*	Early Intervention physiotherapy *n=36*	Difference 95% CI		
											Mean	Lower	Upper
**ODI**[1](SD)	45.2(17.4)	44.6 (19.5)	29.1(16.1)	24.0(18.7)	16.8(19.2)	16.0(19.0)	8.8(11.3)	11.3(15.5)	16.6(11.4)	16.0(14)	-0.6	-6.8	5.6
**VAS Back**[2] (SD)	6.0(2.6)	5.4(3.3)	4.6(2.7)	3.7(2.6)	3.1(2.5)	2.6(2.5)	2.1(2.1)	2.7(2.2)	2.9(1.5)	2.0(2.2)	0.5	1.2	0.3
**VAS Leg**[2] (SD)	6.9(2.3)	7.2(1.8)	5.2(2.9)	4.1(3.0)	2.6(2.9)	2.0(2.5)	0.9(2.2)	1.6(2.2)	2.6(1.6)	2.3(1.8)	-0.3	-1.1	0.6
**EQ5D5L**[3]VAS (SD)	64.6(18.9)	63.8(20.6)	68.9(16.4	72.7(17.7)	73.2(22.9)	79.6(17.5)	81.7(12)	79.6(16.3)	65.8(12.9)	68.4(13.9)	2.6	-3.9	9.1
**EQ5D-5L**[4] Utility score (SD)	0.52(0.25)	0.44(0.29)	0.7(0.26)	0.74(0.22)	0.83(0.23)	0.85(0.22)	0.92(0.12)	0.86(0.19)	0.72(0.15)	0.71(0.17)	-0.01	-0.09	0.07

[1]Oswestry Disability Index 0-100, higher score=higher level of self-rated disability.

[2]Visual Analogue Scale 0-10, higher score=higher self-report pain.

[3] EQ5D-5L VAS score, 0-100, self-rated health. the higher the score, the better the quality of life.

[4] EQ5D-5L Utility score, -0.6 to 1.00 with a higher score representing better quality of life.

For peer review only - http://bmjopen.bmj.com/site/about/guidelines.xhtml

Discussion

This pilot study is the first to explore the role of early intervention physiotherapy for LRS. The study aimed to determine the feasibility of carrying out a full-scale RCT to determine the effectiveness of early physiotherapy for LRS. All of the feasibility parameters were found to be acceptable, including the set-up of G.P centres to recruit participants, recruitment of participants and the retention of 85% of participants at 26 weeks. Both groups received the intervention at the appropriate time, within 2 weeks of randomisation for the early intervention physiotherapy group and after 6 weeks for the usual care group. The acceptance of the intervention, judged by the rate of attendance by participants at their treatment sessions, was better than anticipated.

There were some limitations to this study. Firstly, although recruitment was satisfactory and ahead of time, the G.Ps involved in the study were well motivated and supportive of the study, in a city with a proven track-record of G.P involvement in service development. This may not be the case across the country and further afield. Similarly, the support of the service provider clinical, administrative and management staff was a key factor in the success of the study, a factor which may not be reproducible in other centres. Patients self-referred into the study after an introduction from their G.P (a pre-requisite for ethics approval) and so this group of patients may not be representative of a wider population. These factors need to be taken in account when planning a definitive study, and we have taken a more conservative view of attrition in the definitive sample size calculation. Our recommendations about recruitment also suggest including a wider geographical spread of G.P centres to help meet the proposed recruitment rates. Site selection would need to consider current service provision and the ability to deliver the intervention in settings that are convenient and accessible to patients.

The strengths of the study are that it was a pragmatic study in a clinical setting, using clinical staff and available resources and as such represents the real world of the NHS. We demonstrated that the study is feasible and the potential of early intervention physiotherapy to improve patient care.

Conclusion

The POLAR study results indicate that a full-scale trial of early physiotherapy to treat patients with LRS is feasible. As there is a dearth of evidence about how and when best to treat this population, we conclude that a definitive trial is needed to help inform clinical practice.

For peer review only - http://bmjopen.bmj.com/site/about/guidelines.xhtml

Other information

Ethical review

Ethical approval was received from NHS Scotland, East of Scotland Research Ethics Service (EoSRES) in August 2015 (REC reference 15/ES/0130). The study was conducted in accordance with the declaration of Helsinki and local governance requirements.

Trial Registration

ISRCTN: 25018352

Clinical Trials.Gov: NCT02618278

Acknowledgments

The authors would like to thank the members of the Patient, Public Involvement and Engagement (PPIE) group (Paul Colton, Amanda Dempsey, Rachel Harrison and Claire Village) for their invaluable support and guidance. Thank-you to Dr Jon Dickson, Mark Rhymes and Mark Pinkerton of the Yorkshire and Humber CRN. Thank-you to the wonderful local physiotherapy service for their support, especially the fantastic physiotherapists who delivered the intervention and management (Sarah Withers & Helen Wilson). Finally, a massive thank-you to the participants.

Contributors

MR – Instigated the idea for the study, developed the funding proposal and applied for funding. He developed the protocol, intervention handbook, gained ethical approval and acted as CI for the study.

SW – Is the primary PhD supervisor for MRs' fellowship and contributed to the study conception, design, and writing of the protocol and provided guidance with the statistical analysis.

JC – Is an academic supervisor for MR and has provided specific guidance on protocol development, regulatory approvals and the design of the study.

SB – Provided input regarding the qualitative and mixed method design and analysis aspects of the study.

AAC- Provides clinical supervision for M.R. He has been involved in the conception of the study, its organisation, analysis and writing.

All authors read and commented on drafts, and approved the final version of the manuscript.

For peer review only

For peer review only - http://bmjopen.bmj.com/site/about/guidelines.xhtml

Competing interests

MR–None

SW-None

JC-None

SB-None

AAC-None

All authors have completed the Unified Competing Interest form at www.icmje.org/coi_disclosure.pdf (available on request from the corresponding author) and declare (1) No financial support for the submitted work from anyone other than their employer; (2) No financial relationships with commercial entities that might have an interest in the submitted work; (3) No spouses, partners, or children with relationships with commercial entities that might have an interest in the submitted work; (4) No non-financial interests that may be relevant to the submitted work.

Funding

The lead author (MR) has received a personal Clinical Doctoral Research Fellowship (CDRF) award from Health Education England (HEE) and the National Institute of Health Research (NIHR). Award number: CDRF-2014-05-046.

Disclaimer

This paper presents independent research funded by the National Institute for Health Research (NIHR). The views expressed are those of the author(s) and not necessarily those of the NHS, the NIHR or the Department of Health

Data sharing statement

There are no additional data available for the study which has not been published. All data is available to anyone interested by contacting the corresponding author.

References

1. Koes, Æ Bart W, van Tulder MW PW. The diagnosis and treatment of sciatica. Br Med J. 2007;334:1313–7.
2. Waddell G. The Back Pain Revolution. 2nd editio. Churchill Livingstone, Edinburgh; 2004.
3. Hoy D, March L, Brooks P, Blyth F, Woolf A, Bain C, et al. The global burden of low back pain:

For peer review only - http://bmjopen.bmj.com/site/about/guidelines.xhtml

estimates from the Global Burden of Disease 2010 study. Ann Rheum Dis. 2014;73(6):968–74.

4. Konstantinou K, Dunn KM. Sciatica Review of Epidemiological Studies and Prevalence Estimates. Spine (Phila Pa 1976). 2008;33(22):2464–72.
5. NICE. Low back pain and sciatica in over 16s: assessment and management. Https://Www.Nice.Org.Uk/Guidance/Ng59. 2016.
6. Vroomen PC a J, de Krom MCTFM, Knottnerus J a. Predicting the outcome of sciatica at short-term follow-up. Br J Gen Pract. 2002 Feb;52(475):119–23.
7. Jacobs WCH, van Tulder M, Arts M, Rubinstein SM, van Middelkoop M, Ostelo R, et al. Surgery versus conservative management of sciatica due to a lumbar herniated disc: a systematic review. Eur Spine J. 2011;20(4):513–22.
8. Peul WC, Arts MP, Brand R, Koes BW. Timing of surgery for sciatica: subgroup analysis alongside a randomized trial. Eur Spine J. 2009;18(4):538–45.
9. Peul WC, Brand R, Thomeer RTWM, Koes BW. Improving prediction of "inevitable" surgery during non-surgical treatment of sciatica. Pain. 2008;138(3):571–6.
10. Alentado VJ, Mroz TE, Lubelski D, Steinmetz MP, Benzel EC. Optimal Duration of Conservative Management Prior to Surgery for Cervical and Lumbar Radiculopathy : A Literature Review. Glob Spine J. 2014;279–86.
11. Machado GC., Witzleb AJ., Fritsch C., Maher CG., Ferreira PH., Ferreira ML. d. Patients with sciatica still experience pain and disability 5 years after surgery: A systematic review with meta-analysis of cohort studies. Eur J Pain. 2016;20(March):1–8
12. Lequin MB, Verbaan D, Jacobs WCH, Brand R, Bouma GJ, Vandertop WP, et al. Surgery versus prolonged conservative treatment for sciatica: 5-year results of a randomised controlled trial. BMJ Open. 2013 Jan [cited 2013 Jul 15];3(5):1–10.
13. Suri P, Carlson MJ, Rainville J. Nonoperative Treatment for Lumbosacral Radiculopathy: What Factors Predict Treatment Failure? Clin Orthop Relat Res. 2014;1931–9.
14. Thomas KC, Fisher CG, Boyd M, Bishop P, Wing P, Dvorak MF. Outcome Evaluation of Surgical and Nonsurgical Management of Lumbar Disc Protrusion Causing Radiculopathy. Spine (Phila Pa 1976). 2007;32(13):1414–22.
15. Lau PM-Y, Chow DH-K, Pope MH. Early physiotherapy intervention in an Accident and Emergency Department reduces pain and improves satisfaction for patients with acute low back pain: a randomised trial. Aust J Physiother. 2008 Jan;54(4):243–9.
16. Wand BM, Exspsc G, Bird C, Mcauley JH, Dore CJ, Macdowell M, et al. Early Intervention for the Management of Acute Low Back Pain Education , Manual Therapy , and Exercise. Spine (Phila Pa 1976). 2004;29(21):2350–6.
17. Whitfill T, Haggard R, Bierner SM, Pransky G, Hassett RG, Gatchel RJ. Early intervention options for acute low back pain patients: A randomized clinical trial with one-year follow-up outcomes. J Occup Rehabil. 2010;20(2):256–63.
18. Fritz JM, Childs JD, Wainner RS, Flynn TW. Primary care referral of patients with low back pain to physical therapy: impact on future health care utilization and costs. Spine (Phila Pa 1976). 2012 Dec 1;37(25):2114–21.
19. Gellhorn AC, Chan L, Martin B, Friedly J. Management patterns in acute low back pain: the role of physical therapy. Spine (Phila Pa 1976). 2012 Apr 20;37(9):775–82.
20. Lewis R, Williams NH, Matar HE-D, Din N, Fitzsimmons D, Phillips C, et al. The clinical effectiveness and cost-effectiveness of management strategies for sciatica: systematic review and economic model. Heal Technol Assess Winchester Engl. NETSCC; 2011;15(39):1–578.
21. Rihn JA, Hilibrand AS, Radcliff K, Kurd M, Lurie J, Blood E, et al. Duration of Symptoms Resulting from Lumbar Disc Herniation: Effect on Treatment Outcomes. J Bone Jt Surg. 2011;93–A(20):1906–14.
22. Reddington M, Walters SJ, Cohen J, Baxter S. Does early intervention improve outcomes in physiotherapy management of lumbar radicular syndrome? A mixed-methods study protocol. BMJ Open. 2017;7(3):e014422.
23. Fairbank JC, Pynsent PB. The Oswestry Disability Index. Spine (Phila Pa 1976). 2000 Nov 15;25(22):2940–52.
24. Hahne AJ, Ford JJ, Hinman RS, Richards MC, Surkitt LD, Chan AYP, et al. Individualized functional restoration as an adjunct to advice for lumbar disc herniation with associated radiculopathy. A preplanned subgroup analysis of a randomized controlled trial. Spine J.; 2017;17(3):346–59.
25. Hahne AJ, Ford JJ, Surkitt LD, Richards MC, Chan AYP, Slater SL, et al. Individualized Physical Therapy Is Cost-Effective Compared With Guideline-Based Advice for People With

For peer review only - http://bmjopen.bmj.com/site/about/guidelines.xhtml

Low Back Disorders. Spine (Phila Pa 1976). 2017;42(3):E169–76.
26. Hill JC, Dunn KM, Lewis M, Mullis R, Main CJ, Foster NE, et al. A primary care back pain screening tool: identifying patient subgroups for initial treatment. Arthritis Rheum. 2008;59(5):632–41.
27. EuroQol Group. EuroQol--a new facility for the measurement of health-related quality of life. Health Policy. 1990;16(3):199–208.
28. Teare MD, Dimairo M, Shephard N, Hayman A, Whitehead A, Walters SJ. Sample size requirements to estimate key design parameters from external pilot randomised controlled trials: a simulation study. Trials. 2014;15(1):264.
29. Fritz JM, Irrgang J.J. A Comparison of a Modified Oswestry Low Back Pain Disability Questionnaire and the Quebec Back Pain Disability Scale. Phys Ther. 2001;81(2):776–88.
30. Ostelo RWJG, Deyo RA, Stratford P, Waddell G, Croft P, Korff M Von, et al. Interpreting Change Scores for Pain and Functional Status in Low Back Pain. Spine (Phila Pa 1976). 2008;33(1):90–4.
31. Nicholas MK, George SZ. Psychologically informed interventions for low back pain: an update for physical therapists. Phys Ther. 2011;91(5):765–76.
32. Blyth FM, Macfarlane GJ, Nicholas MK. The contribution of psychosocial factors to the development of chronic pain: the key to better outcomes for patients? Pain. 2007;129(1–2):8–11.
33. Linton SJ, Shaw WS. Impact of psychological factors in the experience of pain. Phys Ther. 2011 May;91(5):700–11.
34. Coppieters MW, Butler DS. Do “sliders” slide and “tensioners” tension? An analysis of neurodynamic techniques and considerations regarding their application. Man Ther. 2008;13(3):213–21.
35. Basson A, Olivier B, Ellis R, Coppieters M, Stewart A, Mudzi W. The Effectiveness of Neural Mobilization for Neuro-Musculoskeletal Conditions: A Systematic Review and Meta-Analysis. J Orthop Sport Phys Ther. 2017;47(1401):1–76.
36. Efstathiou MA, Stefanakis M, Savva C, Giakas G. Effectiveness of neural mobilization inpatients with spinal radiculopathy: Acritical review. J Bodyw Mov Ther. Elsevier Ltd; 2015;19(2):205–12.
37. Butler D. The Sensitive Nervous System. 1st ed. NOI Group Publications; 2000.
38. Hengeveld E BK. Maitlands’ Vertebral Manipulation. 8th ed. Churchill Livingstone, Edinburgh; 2013.
39. Otoo, S.K.W, Hendrick, P, Ribeiro D. he comparative effectiveness of advice/education compared to active physiotherapy (manual therapy and exercise) in the management of chronic non-specific low back pain. Phys Ther Rev. 2015;20(1):16–26.
40. Searle A., Spink M, Ho A, Chuter V. Exercise interventions for the treatment of chronic low back pain: A systematic review and meta-analysis of randomised controlled trials. Clin Rehabil. 2015;12:1155–67.
41. Falla D, Whiteley R, Marco C, Hodges P. Therapeutic Exercise. In: Jull G, Moore A, Falla DL, Lewis J, McCarthy C, Sterling M, editors. Grieves Modern Musculoskeletal Physiotherapy. 4th ed. 2015. p. 298–310.
42. O’Sullivan P. Diagnosis and classification of chronic low back pain disorders: maladaptive movement and motor control impairments as underlying mechanism. Man Ther. 2005;10(4):242–55.
43. Lorig KR, Holman HR, Med AB. Self-Management Education : History , Definition , Outcomes , and Mechanisms. Ann Behav Med. 2003;26(1):1–7.
44. Pinto RZ, Maher CG, Ferreira ML, Ferreira PH, Hancock M, Oliveira VC, et al. Drugs for relief of pain in patients with sciatica: systematic review and meta-analysis. BMJ. 2012 Feb 13;344(feb13 1):e497–e497.
45. Clarke CL, Ryan CG, Martin DJ. Pain neurophysiology education for the management of individuals with chronic low back pain: A systematic review and meta-analysis. Man Ther. 2011;16(6):544–9.

Figure 1 POLAR CONSORT flowchart

Figure 2 Leg pain and ODI scores across groups

For peer review only

For peer review only - http://bmjopen.bmj.com/site/about/guidelines.xhtml

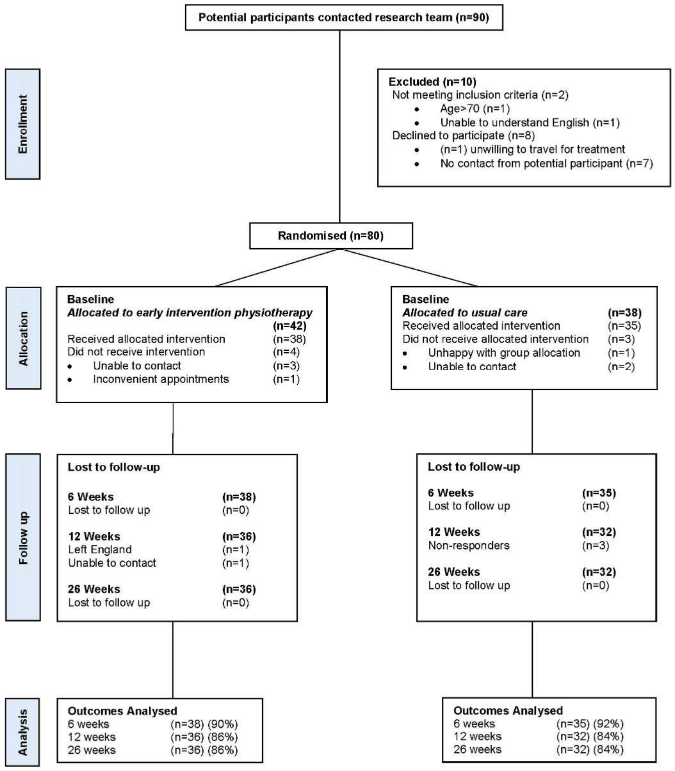

Figure 1 POLAR CONSORT flowchart

209x297mm (300 x 300 DPI)

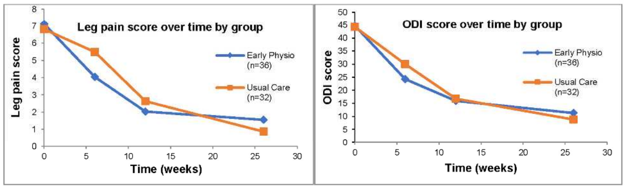

Figure 2 Leg pain and ODI scores across groups

209x297mm (300 x 300 DPI)

Does early intervention improve outcomes in the physiotherapy management of Lumbar Radicular Syndrome?

Logic Model

Intervention

1. Intervention components
a. Movement restriction
b. Neurological treatment
c. Treatment of Psychological barriers
d. Education regarding aetiology/prognosis of the problem
e. Conditioning for function
f. Movement control exercise
g. Pain control
h. Patient-centred-goal orientated care

2. Referral System change
a. Faxed referral
b. Designated slots for early intervention

3. Training
a. Specialist training for participating physiotherapists in diagnosis & management of LRS
b. Referral training for G.Ps

Short-term outcomes

1. Goal Achievement
- Individual, dynamic SMART goal achievement

2. Physical Factors
- Improved movement
- Improved neurological signs &/or symptoms
- Improved conditioning for function
- Improved uni or multi-planar neuro-muscular control
- Improved pain control
- Improve meaningful function

3. Psychological factors
- Decreased psychological barriers a to successful outcome
- Improved understanding of the problem(s)
- Increase confidence in movement & function

System Change
- Available capacity for appointments
- G.Ps able to refer for physiotherapy immediately
- Improved quality of referrals

Training
- Improved specialist knowledge & skills for physiotherapists

Moderating & Mediating Factors

Patient Factors
- Acceptability of the intervention
- Fear (kinesophobia & prognosis)
- Lack of understanding
- Perception of aetiology
- Perception of prognosis
- Perceived value of physiotherapy
- Availability of parking
- Age of patient
- Degree of severity

G.P factors
- Perception of physiotherapy & service provider
- Knowledge of LRS
- Fear (litigation-CES, complaint)
- Research burden
- Research interest

Physiotherapy factors
- Training & support

System-related factors
- Booking POLAR patients into correct appointments slots
- POLAR slot availability

Outcomes

Physiotherapists
- Job satisfaction
- Increased knowledge
- Increased confidence in treating patients with LRS

Patient
- Return to function
- Satisfaction with service
- Time to treatment

Potential Impact

Referral pathway

Service delivery efficiency

Cost effectiveness

Does early intervention improve outcomes in the physiotherapy management of Lumbar Radicular Syndrome? Logic Model V1 29.6.16

For peer review only

POLAR CONSORT 2010 checklist of information to include when reporting a pilot or feasibility trial*

Section/Topic	Item No	Checklist item	Reported on page No
**Title and abstract**			
	1a	Identification as a pilot or feasibility randomised trial in the title	1
	1b	Structured summary of pilot trial design, methods, results, and conclusions (for specific guidance see CONSORT abstract extension for pilot trials)	2,3
**Introduction**			
Background and objectives	2a	Scientific background and explanation of rationale for future definitive trial, and reasons for randomised pilot trial	4
	2b	Specific objectives or research questions for pilot trial	5
**Methods**			
Trial design	3a	Description of pilot trial design (such as parallel, factorial) including allocation ratio	5,6
	3b	Important changes to methods after pilot trial commencement (such as eligibility criteria), with reasons	5
Participants	4a	Eligibility criteria for participants	6
	4b	Settings and locations where the data were collected	5
	4c	How participants were identified and consented	6
Interventions	5	The interventions for each group with sufficient details to allow replication, including how and when they were actually administered	7
Outcomes	6a	Completely defined prespecified assessments or measurements to address each pilot trial objective specified in 2b, including how and when they were assessed	7, 8
	6b	Any changes to pilot trial assessments or measurements after the pilot trial commenced, with reasons	N/A
	6c	If applicable, prespecified criteria used to judge whether, or how, to proceed with future definitive trial	5
Sample size	7a	Rationale for numbers in the pilot trial	8
	7b	When applicable, explanation of any interim analyses and stopping guidelines	N/A
Randomisation:			
Sequence generation	8a	Method used to generate the random allocation sequence	6
	8b	Type of randomisation(s); details of any restriction (such as blocking and block size)	6
Allocation concealment mechanism	9	Mechanism used to implement the random allocation sequence (such as sequentially numbered containers), describing any steps taken to conceal the sequence until interventions were assigned	6

For peer review only

Implementation	10	Who generated the random allocation sequence, who enrolled participants, and who assigned participants to interventions	6
Blinding	11a	If done, who was blinded after assignment to interventions (for example, participants, care providers, those assessing outcomes) and how	N/A
	11b	If relevant, description of the similarity of interventions	7
Statistical methods	12	Methods used to address each pilot trial objective whether qualitative or quantitative	8
**Results**			
Participant flow (a diagram is strongly recommended)	13a	For each group, the numbers of participants who were approached and/or assessed for eligibility, randomly assigned, received intended treatment, and were assessed for each objective	9
	13b	For each group, losses and exclusions after randomisation, together with reasons	9
Recruitment	14a	Dates defining the periods of recruitment and follow-up	10
	14b	Why the pilot trial ended or was stopped	10
Baseline data	15	A table showing baseline demographic and clinical characteristics for each group	10
Numbers analysed	16	For each objective, number of participants (denominator) included in each analysis. If relevant, these numbers should be by randomised group	10,11,12
Outcomes and estimation	17	For each objective, results including expressions of uncertainty (such as 95% confidence interval) for any estimates. If relevant, these results should be by randomised group	11,12
Ancillary analyses	18	Results of any other analyses performed that could be used to inform the future definitive trial	
Harms	19	All important harms or unintended effects in each group (for specific guidance see CONSORT for harms)	12,13
	19a	If relevant, other important unintended consequences	N/A
**Discussion**			
Limitations	20	Pilot trial limitations, addressing sources of potential bias and remaining uncertainty about feasibility	18
Generalisability	21	Generalisability (applicability) of pilot trial methods and findings to future definitive trial and other studies	18
Interpretation	22	Interpretation consistent with pilot trial objectives and findings, balancing potential benefits and harms, and considering other relevant evidence	18
	22a	Implications for progression from pilot to future definitive trial, including any proposed amendments	18, 19
**Other information**			
Registration	23	Registration number for pilot trial and name of trial registry	19
Protocol	24	Where the pilot trial protocol can be accessed, if available	3
Funding	25	Sources of funding and other support (such as supply of drugs), role of funders	20
	26	Ethical approval or approval by research review committee, confirmed with reference number	19

For peer review only

Citation: Eldridge SM, Chan CL, Campbell MJ, Bond CM, Hopewell S, Thabane L, et al. CONSORT 2010 statement: extension to randomised pilot and feasibility trials. BMJ. 2016;355.

*We strongly recommend reading this statement in conjunction with the CONSORT 2010, extension to randomised pilot and feasibility trials, Explanation and Elaboration for important clarifications on all the items. If relevant, we also recommend reading CONSORT extensions for cluster randomised trials, non-inferiority and equivalence trials, non-pharmacological treatments, herbal interventions, and pragmatic trials. Additional extensions are forthcoming: for those and for up to date references relevant to this checklist, see www.consort-statement.org.

For peer review only

Does early intervention improve outcomes in the physiotherapy management of Lumbar Radicular Syndrome? Results of the POLAR Pilot Randomised Controlled Trial

Journal:	*BMJ Open*
Manuscript ID	bmjopen-2018-021631.R1
Article Type:	Research
Date Submitted by the Author:	14-Mar-2018
Complete List of Authors:	Reddington, Michael; Sheffield Teaching Hospitals NHS Foundation Trust, Therapy Services<br>Walters, Stephen; University of Sheffield, ScHARR<br>Cohen, Judith; Hull York Medical School, Clinical Trials Research Unit<br>Baxter, Susan; University of Sheffield, ScHARR<br>Cole, Ashley; Sheffield Teaching Hospitals NHS Foundation Trust, Orthopaedic
**Primary Subject Heading**:	Rehabilitation medicine
Secondary Subject Heading:	Qualitative research, Research methods, Patient-centred medicine, General practice / Family practice
Keywords:	Back pain < ORTHOPAEDIC & TRAUMA SURGERY, Musculoskeletal disorders < ORTHOPAEDIC & TRAUMA SURGERY, Spine < ORTHOPAEDIC & TRAUMA SURGERY

SCHOLARONE™
Manuscripts

For peer review only - http://bmjopen.bmj.com/site/about/guidelines.xhtml

Does early intervention improve outcomes in the physiotherapy management of Lumbar Radicular Syndrome? Results of the POLAR Pilot Randomised Controlled Trial

Corresponding author:

Michael Reddington
Therapy Services Outpatient Department
Sheffield Teaching Hospitals NHS Foundation Trust
Northern General Hospital
Herries Road
Sheffield S5 7AU
MReddington1@sheffield.ac.uk

Professor Stephen J Walters
Designs, Trials and Statistics
ScHARR, University of Sheffield
30 Regents Court
Sheffield S1 4DA
S.J.Walters@sheffield.ac.uk

Dr Judith Cohen
Hull Health Trials Unit
Hull York Medical School, University of Hull
Allam Medical Building, Cottingham Road
Hull HU6 7RX
J.Cohen@hull.ac.uk

Dr Susan Baxter
Section of Public Health
ScHARR, University of Sheffield
30 Regents Court
Sheffield S1 4DA
s.k.baxter@sheffield.ac.uk

Mr Ashley Cole
Consultant Orthopaedic Spinal Surgeon
Sheffield Teaching Hospitals NHS Foundation Trust
Northern General Hospital
Herries Road
Sheffield S5 7AU
Ashley.Cole@sth.nhs.uk

For peer review only

Abstract

Objective

To investigate the feasibility of undertaking a definitive RCT.

Setting

This was a pilot, pragmatic superiority Randomised Controlled Trial (RCT) with a qualitative element, recruiting from 14 General Practitioner (G.P) practices in England.

Participants

Patients over 18 presenting to their G.P with unilateral Lumbar Radicular Syndrome (LRS) defined as radicular pain and/or neurological symptoms originating from lumbar nerve roots, were eligible to participate in the study, those who did not have a clear understanding of the English language or had co-morbidities preventing rehabilitation were ineligible.

Interventions

Participants were randomised into early intervention physiotherapy or usual care with the former receiving their treatment within 2 weeks after randomisation and the latter 6 weeks post randomisation. Both groups received a patient-centred, goal orientated physiotherapy programme specific to their needs. Participants received up to 6 treatment sessions over an 8-week period.

Outcome measures

Process outcomes to determine the feasibility of the study and an exploratory analysis of patient reported outcomes including self-rated disability, pain and general health, these were collected at baseline, 6, 12 and 26 weeks post randomisation.

Results

80 participants were recruited in 10 G.P practices over 34 weeks and randomised to (early intervention physiotherapy n= 42, usual care n=38). Follow-up rates at 26 weeks were 32 (84%) in the usual care and 36 (86%) in the early intervention physiotherapy group. The mean area under the curve for the Oswestry Disability Index (ODI) over the 26 weeks was 16.6 (SD 11.4) in the Usual care group and 16.0 (SD 14.0), in the intervention group. A difference of -0.6 (95% CI: -0.68 to 5.6).

For peer review only - http://bmjopen.bmj.com/site/about/guidelines.xhtml

Conclusions

The results of the study suggest a full RCT is feasible and will provide evidence as to the optimal timing of physiotherapy for patients with LRS.

Trial Registration number ISRCTN: 25018352

Strengths and limitations of the study

- This pilot RCT was conducted in the usual care setting with clinical staff delivering the intervention.
- All feasibility objectives were met, including recruitment and participant attrition, and so the study can directly inform the design and conduct of a definitive RCT.
- Participants self-referred into the study after an introduction from their G.P (a pre-requisite for ethics approval) and so this group of patients may not be representative of a wider population.
- This was a pilot RCT and as such all analyses are exploratory.

Protocol

The protocol for the POLAR study was published and can be accessed at: http://bmjopen.bmj.com/content/bmjopen/7/3/e014422.full.pdf

For peer review only

Introduction

Lumbar Radicular Syndrome (LRS) is a painful and disabling condition, usually of benign causation and in around 90% of cases associated with an intervertebral disc prolapse [1]. It can be self-limiting, lasting a short time with no significant sequelae or can be a major cause of prolonged disability, work loss and long-term presentation to healthcare with associated costs [2,3]. Lifetime prevalence of LRS is estimated to be between 1% and 43% [4] with an annual incidence of between 1% and 5% [5]. Around 75% of LRS sufferers will have symptom resolution by 12 weeks, alongside spontaneous resorption of the Inter Vertebral Disc (IVD) [6]. However, there is no reliable predictor of early, late or no recovery at all. Treatment guidelines encourage initial conservative management in the first instance before considering surgery. Surgery for patients with LRS has been advocated, with good early reported outcomes [7]. The optimum timing for surgery appears to be between 4 weeks and 6 months after symptom onset. However, superiority studies of surgery and conservative management show a quicker improvement in surgical groups, but at a year results show no significant differences [8–11]. Interpretation of these studies is complicated by cross-over from the non-surgical to surgical group. Furthermore, a significant number of patients never have any substantial relief from surgery with unsatisfactory outcomes in over 20% of patients at 5 years [12–13].

Physiotherapy for LRS is commonly employed in the United Kingdom for the management of LRS however, there is a lack of consensus on the type, duration and timing of the physiotherapy intervention [14]. It is known that patients prefer and have improved outcomes with early intervention physiotherapy for Low Back Pain (LBP) [15–17] and that delayed initiation of physiotherapy for LRS increases healthcare consumption [18], no such evidence exists for LRS. Patients who received physiotherapy less than 4 weeks after onset of their LBP had lower healthcare usage and associated costs than those who received physiotherapy more than 3 months after symptom onset [19]. This suggests early treatment is important in terms of cost-savings and prevention of chronic symptom development [20] as increased symptom duration leads to worse outcomes for patients who undertake conservative or surgical care [21-22]. The decision about when to send patients with LRS for physiotherapy is difficult, too early and you may be treating patients who would enjoy spontaneous resolution, too late and the optimum window of treatment including surgery, may have closed.

For peer review only

Aims and Objectives

The study aim was to investigate the feasibility of undertaking a full Randomised Controlled Trial (RCT) to determine the effectiveness and cost-effectiveness of early intervention physiotherapy for patients with LRS.

Process Objectives

1. Successfully set-up recruitment sites in G.P practices.
2. Achieve a recruitment rate of 7 participants per month.
3. Demonstrate the ability to organise 75% of physiotherapy appointments within 2 weeks of randomisation.
4. Provide an appointment within 20 days of randomisation for >75% of participants randomised to the intervention group.
5. Achieve a participant attendance at >66% of physiotherapy appointments.
6. Achieve a participant attrition rate of <25% over the course of the study.
7. Achieve 80% return of Patient Reported Outcome Measures (PROMS) at 6/52 follow-up.

Research objectives

1. To test the feasibility, practicality, safety and acceptability of the study design and protocol.

2. Demonstrate acceptability of the primary and secondary outcome measures to patients and clinicians.

3. To inform the sample size calculation for the definitive RCT trial.

Methods

Design and setting

This was a mixed methods study comprising of an external pilot RCT with an embedded qualitative component in the form of stakeholder interviews in 14 G.P practices in a large city in England. Known as the POLAR study, the pilot RCT will be presented in this paper. A change was made to the inclusion criteria after 1 week of recruitment, the upper age limit of 70 was removed as this excluded a number of potential participants. The protocol for the study has been published, including extensive details of methods [23].

Patient and Public Involvement

The research question was informed directly from patient feedback on physiotherapy services. Current and past patients who have experienced LRS and current physiotherapy and or surgical services were involved from the inception to the end of the study in various ways. Firstly, they were involved in developing the research question, iteration of the intervention and the study processes. They were invaluable in developing patient information and insight into recruitment strategies. Finally, they have been actively involved in the interpretation of the results and discussions of where to go in the next stage of the study. Results will be distributed by email or post to participants who opted to receive the results at consent.

Randomisation

Information from the baseline dataset was used to randomise the participants using a web-based system. The Oswestry Disability Index 2.0 (ODI) [24] was used as the stratification factor with 3 levels based on ODI severity [25]; 'mild & moderate' (≤22-40%), 'severe' (>40 to 60%) and 'crippled' (>60 to 80%). A blinded block size was used to minimise predictability. The random allocation sequence and block size, stratified by centre and ODI disability score was independently generated by the Sheffield Clinical Trials Research Unit (CTRU).

Participants were informed of their group allocation within 1 working day of their consent and randomisation. Participants were randomised to treatment at either 2 or 6 weeks post-randomisation, we were unable to blind either patients or clinicians to the treatment allocation as it was obvious at what time-point they were receiving treatment. In an effort to minimise bias, both groups of patients received protocolised treatment based on the same assessment and treatment framework at the different time points.

Participants

Potential participants with a clinical diagnosis of LRS were identified by their G.P and given details of the study. Each participating G.P underwent training and were equipped with a diagnostic aide memoire for clinically identifying patients with LRS. If interested, they contacted a member of the research team who screened for eligibility and arranged to meet to discuss the study. Anyone over the age of 18 years with unilateral LRS and who could speak English were eligible. If they had 'red

For peer review only

flag' signs or symptoms such as cancer, cauda equine syndrome, spinal fracture or had other physical or psychological disabilities preventing rehabilitation, they were ineligible.

Recruitment & consent

Written consent was obtained by the research team after meeting the potential participant and confirming eligibility criteria including the clinical diagnosis of LRS. There were three recruitment cycles, each lasting up to 12 weeks or until 27 participants had been recruited for that cycle (26 for the final cycle). The remaining eight weeks were used for completion of treatment. A two-week period between cycles provided time to reflect and analyse the results from the stakeholder interviews and other feedback to refine the study processes as necessary.

The Intervention

The intervention was protocolised and allowed the treating physiotherapist a range of treatment options within each domain with the selected options recorded electronically for each treatment session. The goal-orientated physiotherapy regimen for both groups were tailored to the individuals' requirements based on the information gathered from the baseline interview data, PROMS and clinical assessment. Participants were assessed using a multi-dimensional approach based on seven different elements; psychological barriers to recovery, neurological factors, movement restriction, understanding, conditioning, movement control and pain. Individualised physiotherapy for LBP and LRS is known to be superior and more cost-effective than advice alone [26,27], it is flexible and directly relevant to the individual and their changing needs. Participants received a maximum of six sessions of physiotherapy over an eight-week period, fewer if their pre-determined goals had been achieved. A logic model has been developed for the intervention which can be found in the supplementary material.

Treatment Fidelity

Several strategies were employed to optimise fidelity, including a protocolised training package for the treating physiotherapists, standardised patient information, weekly feedback and support of treating physiotherapists and video analysis of each participating physiotherapist treating a study participant. The study took place in an NHS community setting using three physiotherapists, already employed by

For peer review only

the host service provider. The physiotherapists had a mean age of 36 years (range 34-40 years) and a mean of 10 years postgraduate experience (range 7-12 years). They underwent 21 hours of training in the assessment and intervention and to promote and facilitate self-management, optimal function, pacing advice, analgesic advice together with equipping the patient with coping strategies.

Outcomes

Patients were asked to complete self-report and screening measures by post or face to face at four-time points: firstly, at the time of consent and then at 6, 12 and 26 weeks post randomisation**.** The primary outcomes for the study were process outcomes as the objective was to determine the feasibility of carrying out a full-scale RCT. Secondary outcomes were the ODI, Visual Analogue Scale (VAS) for back and leg pain, Keele STarT Back score [28], EQ5D-5L [29] and a self-report form focussing on functional loss, goals and medical history.

Sample size

It has been recommended that an external pilot study should have at least 70 measured participants (35 per group) when estimating the standard deviation for a continuous outcome [30]. A sample size of 80 patients, with approximately 10% allowance for loss to follow-up allows the standard deviation of an outcome to be estimated to within a precision of approximately ±16% of its true underlying value with 95% confidence.

Results

The flow chart of the participant journey for the POLAR study can be viewed in Figure 1. Ninety potential participants who were given details of the study by their respective G.Ps contacted the research team. Ten were excluded as they either did not meet the inclusion criteria or refused to be randomised, with 80 going on to be randomised from 10 different primary care G.P practices.

Baseline characteristics

The baseline characteristics of all participants, by group can be found in Table 1. This illustrates the comparability of the 2 arms with no evidence of selection bias. The groups were well matched for demographic factors such as age, gender and BMI as well as levels of disability, pain in leg and back,

For peer review only

risk of chronicity and general health status. However, there was evidence of a difference in the EQ-5D utility scores but not the EQ-5D VAS which is attributable to chance as all participants were randomised. The early intervention physiotherapy group had longer symptom duration going into the study.

Table 1 Baseline characteristics of POLAR participants

	**Early Intervention physiotherapy**			**Usual Care**			**Total**		
	N	%		N	%		N	%	
**Female**	21	50		18	47		39	49	
**White British**	38	90		33	87		71	89	
	**N**	**Mean**	**SD**	**N**	**Mean**	**SD**	**N**	**Mean**	**SD**
**Age** (years)	42	47	14	38	47	13	80	47	13
**Height (**CM)	42	172.1	10.7	38	172.1	9.8	80	171.7	10.2
**Weight** (KG)	39[1]	81.5	14.8	38	80.6	15.7	77	81	15.2
**BMI**	39[1]	27.7	4.6	38	27.3	5.6	77	27.5	5.1
**ODI score** (%)	42	44.6	19.5	38	45.2	17.4	80	44.9	18.4
**Leg Pain**	42	7.2	1.8	38	6.9	2.3	80	7	2.1
**Back pain**	42	5.4	3.3	38	6	2.6	80	5.7	3.0
**EQ5D-5L** VAS	42	63.8	20.6	38	64.6	18.9	80	64.1	19.7
**EQ5D-5L** Utility score	42	0.44	0.29	38	0.52	0.25	80	0.48	0.27
**Keele STarT-Back**	42	5.7	2.0	38	5.7	1.8	80	5.7	1.9
**Keele STarT-Back** Sub-score	42	2.0	1.5	38	2.7	1.3	80	2.8	1.4
**Time to treatment** (days) [2]	38	11.1	10.5	31	43.6	8.9	69	25.7	19.0
	**N**	**Median**	**IQR**	**N**	**Median**	**IQR**	**N**	**Median**	**IQR**
**Symptoms duration** (days)	42	92	276	38	61	51	80	77	203

[1] 3 missing values

[2] Time between randomisation and first scheduled treatment session

For peer review only

Process Results

The POLAR study is a pilot trial and outlined below are the results of the feasibility objectives.

Set-up of recruitment sites in primary care

Twenty G.P practices were initially approached to take part in the study, with ten agreeing to participate. Towards the end of the second tranche of recruitment it was evident that one practice was recruiting a large number of participants and a decision was made to enrol new recruitment centres. Seven further G.P practices were therefore approached, with four agreeing to participate.

Recruitment rate

Eighty participants were recruited between the period 1st March 2016 and 7th November 2016 with a recruitment rate of 2.4 participants per week or 9.6 participants per month which enabled recruitment to end earlier than anticipated. Forty-two participants were randomised into the early intervention group and 38 in the usual care group.

Organisation of physiotherapy appointments

The target of 75% of physiotherapy appointments being made within two weeks of randomisation was surpassed in both groups. 100% (42/42) (95% CI: 92% to 100%) of early intervention physiotherapy participants received their appointment within 20 days of randomisation and 38/38 (95% CI:91% to 100%) in the usual care group. This illustrates the feasibility of making appointments for participants at short notice.

The feasibility of intervention delivery

A key feasibility parameter was the ability for at least 75% of early intervention physiotherapy participants to be seen by a physiotherapist, within 20 days of randomisation. 100% (42/42) (95% CI 92% to 100%) of participants reached this target, with a mean of 14.1 days between randomisation and first treatment session.

Participant treatment session attendance

The mean attendance rate for physiotherapy appointments in both groups was 92.6% (SD 16.2), 93.8% (SD 12.6) for the intervention group physiotherapy and 91.1% (SD 19.8) in the usual care group. All surpassed the a priori target of greater than 66% attendance. The mean number of treatment sessions received by the intervention group was 4 (SD=1) and 3 in the usual care group (SD=2).

Participant attrition

Eighty participants agreed to take part in the study. The intervention group attrition rate was 14% (6/42) (95% CI: 7% to 28%) and in the usual care group it was 16% (6/38) (95% CI 7% to 30%) at 26 weeks follow-up. The overall attrition rate for drop out of participants was 15% (95% CI 9% to 24%), all within the a priori limit set at 25%.

For peer review only

Outcome measure return

The outcome measure return rates surpassed expectations of 80% at six weeks and were as follows: 38/42 (91%; 95% CI: 78% to 96%) at six weeks post randomisation for the intervention group and 35/38 (92%; 95% CI 79% to 97%) for the usual care group.

Research results

Analysis of key clinical outcomes

Figure 2 shows the leg pain and ODI scores (likely primary outcome measures for definitive RCT) for participants with all 4 assessments completed. The blue line illustrates the increased rate of recovery in the early intervention physiotherapy group up to 6 weeks. When the usual care group begins their physiotherapy the rate of recovery assimilates and by 12 weeks and both groups have very similar scores.

Two participants underwent lumbar micro-discectomy surgery for their LRS. Both participants had completed their respective courses of physiotherapy before undergoing surgery. S05/005 (usual care) failed to make significant improvements to their pain and with a severe level of pain and disability, surgery was undertaken. S06/027 (early intervention physiotherapy) had made significant improvements with physiotherapy, improving by over 20 points on the ODI, but required surgery due to 'impending' cauda equina syndrome.

The feasibility, practicality, safety and acceptability of the study design and protocol

The feasibility of the study has been suggested by the results of the feasibility parameters. There were several adjustments made to the processes of the study which were made possible by the breaks in recruitment. These included a brief weekly email to all participating G.Ps to remind them of the study and improve the clarity of inclusion and exclusion criteria. A change to the process of administering the six-week outcome measures was necessary, after the physiotherapists reported it too time consuming to administer. There were no changes made to the intervention, which appeared to be well received by both participants and clinicians alike. There were no adverse events or serious adverse events associated with the intervention or the study processes.

Harms

There was one Serious Adverse Event (SAE) during the course of the study in the early intervention physiotherapy group. The SAE rate was 2% (1/42) in the early intervention physiotherapy group and

For peer review only

0% (0/38) in the usual care group a difference of 2% (95% CI -7% to 12%). The participant was hospitalised after suffering a Cerebro-Vascular-Accident (CVA) related to pre-existing vascular hypertension. The participant had completed their physiotherapy intervention two weeks prior and made a full recovery at 6 months. This was reported to the ethics committee and Trial Management Group (TMG).

Acceptability of the primary and secondary outcome measures to patients and clinicians

The importance of examining acceptability of the outcome measures, processes and the intervention was a key area of investigation for the study, and the pilot trial included a qualitative element to explore these aspects. Details of the qualitative aspects of the study will be reported in forthcoming papers. However, in summary the key processes necessary for implementation and evaluation of the study were reported to be acceptable by all stakeholders.

Fidelity

Physiotherapists recorded the components of their treatment sessions at each patient encounter in order to enhance and measure treatment fidelity. Participants in the early intervention physiotherapy group had a mean of 4 treatment sessions and those participants in the usual care group 3 sessions. There were 269 physiotherapy sessions carried out as part of the POLAR study with 1267 component parts (Table 2), 36 (3%) of which outside the protocolled treatment framework. The components outside the protocol consisted of three sessions of acupuncture and exercise other than that in the protocol. Video analysis was carried out independently on a purposive sample of 5 treatment sessions using a fidelity assessment tool developed by the lead author, clinical colleagues and PPIE representatives. The maximum score for ‘essential’ aspects of fidelity was 15/15. The median score for the videos was 14/15 (93%) with a range of 13-15 (87-100%).

Sample size calculation for the definitive RCT trial

For the definitive RCT we propose the primary outcome is the ODI at 26-weeks post-randomisation as the ODI has shown to be acceptable to patients and a commonly used measurement of self-rated disability. In this pilot trial, we observed a difference in means of 2.5 points (95% CU: -4.5 to 9.1) between the randomised groups and a standard deviation of 16-points at 26 weeks. There is a lack of consensus regarding the Minimum Clinically Important Difference (MCID) for the ODI, with

For peer review only - http://bmjopen.bmj.com/site/about/guidelines.xhtml

suggestions ranging from 6% to 30% (31,32). If we assume a target difference of five-points on the ODI scale then with 217 patients per group (434 in total) we would have 90% power to detect a five-point difference or more (equivalent to standardised effect size of 0.31) between the randomised groups which would be statistically significant at the 5% two-sided level. Allowing for a conservative estimate of 20% attrition (we observed 15% in this pilot) we would need to recruit and randomise 272 per group (544 in total).

Based on the recruitment rates observed in this trial of 80 patients in 8.5 months of recruitment at 10 centres (a rate of 0.9 patients per centre month); the main trial would need around 24 centres recruiting for 24 months to achieve this target.

The descriptive statistics for all participants by group and time point can be found in Table 3.

For peer review only

Table 2 Intervention domains and components frequency table

Domain	No. of participants receiving component N=69	Method of assessment	Treatment options	Frequency of component used	%
**Psychological barriers to recovery** [33-35]	47 (68%)	Keele STarTBack Clinical interview & history	a. Treatment of Kinesiophobia with graded exposure, education and movement re-education	16	1.3
			b. Treatment of hypervigilance with education, distraction & desensitisation	17	1.4
			c. Treatment of faulty beliefs about pain, LRS, treatment and/or prognosis with education and self-management strategies	38	3.2
			d. Treatment of Iatrogenic beliefs and corresponding avoidance behaviours with education and movement re-education	3	0.2
			e. Treatment of aspects of work as a barrier to recovery and treatment with ergonomic advice and practise	15	1.2
			f. Identification of financial barriers to recovery and signposting e.g. debt management	15	1.2
			g. Identification of emotional barriers to recovery and signposting to appropriate therapy e.g. G.P/Psychology	57	4.7
**Neurological** [36–39]	39 (58%)	Clinical assessment	a. Neural interface mobilisation	98	8.1
			b. Functional neurological movement re-education	7	0.6
**Movement restriction** [40]	59 (86%)	Clinical assessment	a. Flexion mobilisation (Grade 2-4)	68	5.6
			b. Side-flexion mobilisation (Gr. 2-4)	5	0.4
			c. Extension mobilisation (Gr. 2-4)	15	1.2
			d. Rotation mobilisation (Gr. 2-4)	41	3.4
			e. Flexion+Side-flexion mobilisation (Gr. 2-4)	11	0.9
			f. Flexion+Side+flexion+rotation mobilisation (Gr. 2-4)	62	5.2
			g. Extension+Side flexion mobilisation (Grade 2-4)	0	0
			h. Manipulation (Gr. 5)	0	0
			i. Seated Mobilisation With Movement (MWM)	16	1.3
			j. Standing MWM	16	1.3
			k. Mobilisation into functional position	14	1.2

For peer review only - http://bmjopen.bmj.com/site/about/guidelines.xhtml

			l. Muscle stretches	61	5.1
			m. Functional movement re-education	7	0.6
**Understanding** [41]			a. Management of erroneous believes relating to LRS provide education to help eradicate these beliefs	57	4.7
			b. Pacing behaviours	53	4.4
			c. Goal attainment	58	4.8
			d. Health Promotion	80	6.6
			e. Identification and treatment of central sensitisation-liaison with G.P/pain clinic	8	0.7
			f. Identification and treatment of peripheral sensitisation-liaison with G.P/pain clinic	7	0.6
**Conditioning** [42,43]	63 (91%)	Self-assessment answers, clinical interview & history	a. Cardiovascular & conditioning exercise relevant to patients' goals	83	6.9
			b. Function specific stretches	39	3.2
			c. Function specific strengthening	62	5.2
			d. Ergonomic advice	14	1.2
			e. Ergonomic practise	6	0.5
			f. Group exercise	0	0.0
			g. Perturbation training	7	0.6
**Movement control** [44]	33 (48%)	Clinical assessment	a. Sagittal plane control in functional positions relevant to patients' problems/goals	24	2.0
			b. Coronal plane control in functional positions relevant to patients' problems/goals	15	1.2
			c. Axial plane control in functional positions relevant to patients' problems/goals	1	0.1
			d. Multi-planar control in functional positions relevant to patients' problems/goals	6	0.5
			e. Movement re-education in functional positions relevant to patients' problems/goals	18	1.5
**Pain** [45–47]	52 (75%)	ODI<br>VAS back & leg<br>Clinical interview & history	a. Analgesic review & advice in liaison with G.P/Pharmacist	23	1.9
			b. Pain education	60	5.0
			c. Pain coping strategies	20	1.7
			d. Fear reduction intervention in liaison with psychologist/pain clinic	12	1.0
			e. Stress reduction intervention in liaison with psychologist/pain clinic	32	2.7
**Totals**				1267	99.8%[1]

[1] 0.2% missing data-2 treatment episodes where components not attributed.

For peer review only - http://bmjopen.bmj.com/site/about/guidelines.xhtml

Table 3 Descriptive statistics for outcome measures at each time point

Outcome	Baseline		6 Weeks		12 Weeks		26 weeks		AUC				
	Usual Care *n=38*	Early Intervention physiotherapy *n=42*	Usual Care *n=35*	Early Intervention physiotherapy *n=38*	Usual Care *n=32*	Early Intervention physiotherapy *n=36*	Usual Care *n=32*	Early Intervention physiotherapy *n=36*	Usual Care *n=32*	Early Intervention physiotherapy *n=36*	Difference 95% CI		
											Mean	Lower	Upper
**ODI**[1](SD)	45.2(17.4)	44.6 (19.5)	29.1(16.1)	24.0(18.7)	16.8(19.2)	16.0(19.0)	8.8(11.3)	11.3(15.5)	16.6(11.4)	16.0(14)	-0.6	-6.8	5.6
**VAS Back**[2] (SD)	6.0(2.6)	5.4(3.3)	4.6(2.7)	3.7(2.6)	3.1(2.5)	2.6(2.5)	2.1(2.1)	2.7(2.2)	2.9(1.5)	2.0(2.2)	0.5	1.2	0.3
**VAS Leg**[2] (SD)	6.9(2.3)	7.2(1.8)	5.2(2.9)	4.1(3.0)	2.6(2.9)	2.0(2.5)	0.9(2.2)	1.6(2.2)	2.6(1.6)	2.3(1.8)	-0.3	-1.1	0.6
**EQ5D5L**[3]VAS (SD)	64.6(18.9)	63.8(20.6)	68.9(16.4	72.7(17.7)	73.2(22.9)	79.6(17.5)	81.7(12)	79.6(16.3)	65.8(12.9)	68.4(13.9)	2.6	-3.9	9.1
**EQ5D-5L**[4] Utility score (SD)	0.52(0.25)	0.44(0.29)	0.7(0.26)	0.74(0.22)	0.83(0.23)	0.85(0.22)	0.92(0.12)	0.86(0.19)	0.72(0.15)	0.71(0.17)	-0.01	-0.09	0.07

[1]Oswestry Disability Index 0-100, higher score=higher level of self-rated disability.

[2]Visual Analogue Scale 0-10, higher score=higher self-report pain.

[3] EQ5D-5L VAS score, 0-100, self-rated health. the higher the score, the better the quality of life.

[4] EQ5D-5L Utility score, -0.6 to 1.00 with a higher score representing better quality of life.

For peer review only

Discussion

This pilot study is the first to explore the role of early intervention physiotherapy for LRS. The study aimed to determine the feasibility of carrying out a full-scale RCT to determine the effectiveness of early physiotherapy for LRS. All of the feasibility parameters were found to be acceptable, including the set-up of G.P centres to recruit participants, recruitment of participants and the retention of 85% of participants at 26 weeks. Both groups received the intervention at the appropriate time, within 2 weeks of randomisation for the early intervention physiotherapy group and after 6 weeks for the usual care group. The acceptance of the intervention, judged by the rate of attendance by participants at their treatment sessions, was better than anticipated.

There were some limitations to this study. Firstly, although recruitment was satisfactory and ahead of time, the G.Ps involved in the study were well motivated and supportive of the study, in a city with a proven track-record of G.P involvement in service development. This may not be the case across the country and further afield. Similarly, the support of the service provider clinical, administrative and management staff was a key factor in the success of the study, a factor which may not be reproducible in other centres. Patients self-referred into the study after an introduction from their G.P (a pre-requisite for ethics approval) and so this group of patients may not be representative of a wider population. These factors need to be taken in account when planning a definitive study, and we have taken a more conservative view of attrition in the definitive sample size calculation. Our recommendations about recruitment also suggest including a wider geographical spread of G.P centres to help meet the proposed recruitment rates. Site selection would need to consider current service provision and the ability to deliver the intervention in settings that are convenient and accessible to patients.

The strengths of the study are that it was a pragmatic study in a clinical setting, using clinical staff and available resources and as such represents the real world of the NHS. We demonstrated that the study is feasible and the potential of early intervention physiotherapy to improve patient care.

Conclusion

The POLAR study results indicate that a full-scale trial of early physiotherapy to treat patients with LRS is feasible. As there is a dearth of evidence about how and when best to treat this population, we conclude that a definitive trial is needed to help inform clinical practice.

For peer review only - http://bmjopen.bmj.com/site/about/guidelines.xhtml

Other information

Ethical review

Ethical approval was received from NHS Scotland, East of Scotland Research Ethics Service (EoSRES) in August 2015 (REC reference 15/ES/0130). The study was conducted in accordance with the declaration of Helsinki and local governance requirements.

Trial Registration

ISRCTN: 25018352

Clinical Trials.Gov: NCT02618278

Acknowledgments

The authors would like to thank the members of the Patient, Public Involvement and Engagement (PPIE) group (Paul Colton, Amanda Dempsey, Rachel Harrison and Claire Village) for their invaluable support and guidance. Thank-you to Dr Jon Dickson, Mark Rhymes and Mark Pinkerton of the Yorkshire and Humber CRN. Thank-you to the wonderful local physiotherapy service for their support, especially the fantastic physiotherapists who delivered the intervention and management (Sarah Withers & Helen Wilson). Finally, a massive thank-you to the participants.

Contributors

MR – Instigated the idea for the study, developed the funding proposal and applied for funding. He developed the protocol, intervention handbook, gained ethical approval and acted as CI for the study.

SW – Is the primary PhD supervisor for MRs' fellowship and contributed to the study conception, design, and writing of the protocol and provided guidance with the statistical analysis.

JC – Is an academic supervisor for MR and has provided specific guidance on protocol development, regulatory approvals and the design of the study.

SB – Provided input regarding the qualitative and mixed method design and analysis aspects of the study.

AAC- Provides clinical supervision for M.R. He has been involved in the conception of the study, its organisation, analysis and writing.

All authors read and commented on drafts and approved the final version of the manuscript.

For peer review only

Competing interests

MR–None

SW-None

JC-None

SB-None

AAC-None

All authors have completed the Unified Competing Interest form at www.icmje.org/coi_disclosure.pdf (available on request from the corresponding author) and declare (1) No financial support for the submitted work from anyone other than their employer; (2) No financial relationships with commercial entities that might have an interest in the submitted work; (3) No spouses, partners, or children with relationships with commercial entities that might have an interest in the submitted work; (4) No non-financial interests that may be relevant to the submitted work.

Funding

The lead author (MR) has received a personal Clinical Doctoral Research Fellowship (CDRF) award from Health Education England (HEE) and the National Institute of Health Research (NIHR). Award number: CDRF-2014-05-046.

Disclaimer

This paper presents independent research funded by the National Institute for Health Research (NIHR). The views expressed are those of the author(s) and not necessarily those of the NHS, the NIHR or the Department of Health

Data sharing statement

There are no additional data available for the study which has not been published. All data is available to anyone interested by contacting the corresponding author.

For peer review only

References

1. Koes, Æ Bart W, van Tulder MW PW. The diagnosis and treatment of sciatica. Br Med J. 2007;334:1313–7.
2. Waddell G. The Back Pain Revolution. 2nd editio. Churchill Livingstone, Edinburgh; 2004.
3. Hoy D, March L, Brooks P, Blyth F, Woolf A, Bain C, et al. The global burden of low back pain: estimates from the Global Burden of Disease 2010 study. Ann Rheum Dis [Internet]. 2014;73(6):968–74.
4. Konstantinou K, Dunn KM. Sciatica Review of Epidemiological Studies and Prevalence Estimates. Spine (Phila Pa 1976). 2008;33(22):2464–72.
5. NICE. Low back pain and sciatica in over 16s: assessment and management [Internet]. Https://Www.Nice.Org.Uk/Guidance/Ng59. 2016.
6. Vroomen PC a J, de Krom MCTFM, Knottnerus J a. Predicting the outcome of sciatica at short-term follow-up. Br J Gen Pract [Internet]. 2002 Feb;52(475):119–23.
7. Weinstein JN, Lurie JD, Tosteson TD, Tosteson AN a, Blood E a, Abdu W a, et al. Surgical versus nonoperative treatment for lumbar disc herniation: four-year results for the Spine Patient Outcomes Research Trial (SPORT). Spine (Phila Pa 1976) [Internet]. 2008 Dec;33(25):2789–800.
8. Jacobs WCH, van Tulder M, Arts M, Rubinstein SM, van Middelkoop M, Ostelo R, et al. Surgery versus conservative management of sciatica due to a lumbar herniated disc: a systematic review. Eur Spine J [Internet]. 2011;20(4):513–22.
9. Peul WC, Arts MP, Brand R, Koes BW. Timing of surgery for sciatica: subgroup analysis alongside a randomized trial. Eur Spine J [Internet]. 2009;18(4):538–45.
10. Peul WC, Brand R, Thomeer RTWM, Koes BW. Improving prediction of "inevitable" surgery during non-surgical treatment of sciatica. Pain. 2008;138(3):571–6.
11. Alentado VJ, Mroz TE, Lubelski D, Steinmetz MP, Benzel EC. Optimal Duration of Conservative Management Prior to Surgery for Cervical and Lumbar Radiculopathy : A Literature Review. Glob Spine J. 2014;279–86.
12. Machado GC., Witzleb AJ., Fritsch C., Maher CG., Ferreira PH., Ferreira ML. d. Patients with sciatica still experience pain and disability 5 years after surgery: A systematic review with meta-analysis of cohort studies. Eur J Pain (United Kingdom) [Internet]. 2016;20(March):1–8.
13. Lequin MB, Verbaan D, Jacobs WCH, Brand R, Bouma GJ, Vandertop WP, et al. Surgery versus prolonged conservative treatment for sciatica: 5-year results of a randomised controlled trial. BMJ Open [Internet]. 2013 Jan [cited 2013 Jul 15];3(5):1–10.
14. Suri P, Carlson MJ, Rainville J. Nonoperative Treatment for Lumbosacral Radiculopathy: What Factors Predict Treatment Failure? Clin Orthop Relat Res [Internet]. Springer US; 2014;1931–9.
15. Thomas KC, Fisher CG, Boyd M, Bishop P, Wing P, Dvorak MF. Outcome Evaluation of Surgical and Nonsurgical Management of Lumbar Disc Protrusion Causing Radiculopathy. Spine (Phila Pa 1976). 2007;32(13):1414–22.
16. Lau PM-Y, Chow DH-K, Pope MH. Early physiotherapy intervention in an Accident and Emergency Department reduces pain and improves satisfaction for patients with acute low back pain: a randomised trial. Aust J Physiother [Internet]. 2008 Jan;54(4):243–9.
17. Wand BM, Bird C, Mcauley JH, Dore CJ, Macdowell M, Souza LH De. Early Intervention for the Management of Acute Low Back Pain Education , Manual Therapy , and Exercise. Spine (Phila Pa 1976). 2004;29(21):2350–6.
18. Whitfill T, Haggard R, Bierner SM, Pransky G, Hassett RG, Gatchel RJ. Early intervention options for acute low back pain patients: A randomized clinical trial with one-year follow-up outcomes. J Occup Rehabil. 2010;20(2):256–63.
19. Fritz JM, Childs JD, Wainner RS, Flynn TW. Primary care referral of patients with low back pain to physical therapy: impact on future health care utilization and costs. Spine (Phila Pa 1976) [Internet]. 2012 Dec 1 [cited 2014 Jan 31];37(25):2114–21.
20. Gellhorn AC, Chan L, Martin B, Friedly J. Management patterns in acute low back pain: the role of physical therapy. Spine (Phila Pa 1976) [Internet]. 2012 Apr 20 [cited 2014 Feb 3];37(9):775–82.
21. Lewis R, Williams NH, Matar HE-D, Din N, Fitzsimmons D, Phillips C, et al. The clinical effectiveness and cost-effectiveness of management strategies for sciatica: systematic review and economic model. Heal Technol Assess Winchester Engl [Internet]. NETSCC; 2011;15(39):1–578.
22. Rihn JA, Hilibrand AS, Radcliff K, Kurd M, Lurie J, Blood E, et al. Duration of Symptoms

For peer review only - http://bmjopen.bmj.com/site/about/guidelines.xhtml

Resulting from Lumbar Disc Herniation: Effect on Treatment Outcomes. J Bone Jt Surg [Internet]. 2011;93–A(20):1906–14.

23. Reddington M, Walters SJ, Cohen J, Baxter S. Does early intervention improve outcomes in physiotherapy management of lumbar radicular syndrome? A mixed-methods study protocol. BMJ Open [Internet]. 2017;7(3):e014422.
24. Fairbank JC, Pynsent PB. The Oswestry Disability Index. Spine (Phila Pa 1976) [Internet]. 2000 Nov 15;25(22):2940–52; discussion 2952.
25. Fairbank, JC. Couper, J. Davies, JB. O'Brien J. The Oswestry low back pain disability questionnaire. Physiother. 1980;66(8):271–3.
26. Hahne AJ, Ford JJ, Hinman RS, Richards MC, Surkitt LD, Chan AYP, et al. Individualized functional restoration as an adjunct to advice for lumbar disc herniation with associated radiculopathy. A preplanned subgroup analysis of a randomized controlled trial. Spine J. Elsevier Inc.; 2017;17(3):346–59.
27. Hahne AJ, Ford JJ, Surkitt LD, Richards MC, Chan AYP, Slater SL, et al. Individualized Physical Therapy Is Cost-Effective Compared With Guideline-Based Advice for People With Low Back Disorders. Spine (Phila Pa 1976) [Internet]. 2017;42(3):E169–76.
28. Hill JC, Dunn KM, Lewis M, Mullis R, Main CJ, Foster NE, et al. A primary care back pain screening tool: identifying patient subgroups for initial treatment. Arthritis Rheum [Internet]. 2008;59(5):632–41. Available from: http://www.ncbi.nlm.nih.gov/pubmed/18438893
29. EuroQol Group. EuroQol--a new facility for the measurement of health-related quality of life. Health Policy [Internet]. 1990;16(3):199–208.
30. Teare MD, Dimairo M, Shephard N, Hayman A, Whitehead A, Walters SJ. Sample size requirements to estimate key design parameters from external pilot randomised controlled trials: a simulation study. Trials [Internet]. 2014;15(1):264.
31. Fritz JM, Irrgang J.J. A Comparison of a Modified Oswestry Low Back Pain Disability Questionnaire and the Quebec Back Pain Disability Scale. Phys Ther. 2001;81(2):776–88.
32. Ostelo RWJG, Deyo RA, Stratford P, Waddell G, Croft P, Korff M Von, et al. Interpreting Change Scores for Pain and Functional Status in Low Back Pain. Spine (Phila Pa 1976). 2008;33(1):90–4.
33. Nicholas MK, George SZ. Psychologically informed interventions for low back pain: an update for physical therapists. Phys Ther [Internet]. 2011 May [cited 2012 Nov 22];91(5):765–76.
34. Blyth FM, Macfarlane GJ, Nicholas MK. The contribution of psychosocial factors to the development of chronic pain: the key to better outcomes for patients? Pain [Internet]. 2007;129(1–2):8–11.
35. Linton SJ, Shaw WS. Impact of psychological factors in the experience of pain. Phys Ther [Internet]. 2011 May [cited 2012 Oct 28];91(5):700–11.
36. Coppieters MW, Butler DS. Do "sliders" slide and "tensioners" tension? An analysis of neurodynamic techniques and considerations regarding their application. Man Ther. 2008;13(3):213–21.
37. Basson A, Olivier B, Ellis R, Coppieters M, Stewart A, Mudzi W. The Effectiveness of Neural Mobilization for Neuro-Musculoskeletal Conditions: A Systematic Review and Meta-Analysis. J Orthop Sport Phys Ther [Internet]. 2017;47(1401):1–76.
38. Efstathiou MA, Stefanakis M, Savva C, Giakas G. Effectiveness of neural mobilization inpatients with spinal radiculopathy: Acritical review. J Bodyw Mov Ther [Internet]. Elsevier Ltd; 2015;19(2):205–12.
39. Butler D. The Sensitive Nervous System. 1st ed. NOI Group Publications; 2000.
40. Hengeveld E BK. Maitlands' Vertebral Manipulation. 8th ed. Churchill Livingstone, Edinburgh; 2013.
41. Otoo, S.K.W, Hendrick, P, Ribeiro D. he comparative effectiveness of advice/education compared to active physiotherapy (manual therapy and exercise) in the management of chronic non-specific low back pain. Phys Ther Rev. 2015;20(1):16–26.
42. Searle a., Spink M, Ho A, Chuter V. Exercise interventions for the treatment of chronic low back pain: A systematic review and meta-analysis of randomised controlled trials. Clin Rehabil [Internet]. 2015;12:1155–67.
43. Falla D, Whiteley R, Marco C, Hodges P. Therapeutic Exercise. In: Jull G, Moore A, Falla DL, Lewis J, McCarthy C, Sterling M, editors. Grieves Modern Musculoskeletal Physiotherapy. 4th ed. 2015. p. 298–310.
44. O'Sullivan P. Diagnosis and classification of chronic low back pain disorders: maladaptive movement and motor control impairments as underlying mechanism. Man Ther [Internet]. 2005;10(4):242–55.

For peer review only

45. Lorig KR, Holman HR, Med AB. Self-Management Education : History , Definition , Outcomes , and Mechanisms. Ann Behav Med. 2003;26(1):1–7.
46. Pinto RZ, Maher CG, Ferreira ML, Ferreira PH, Hancock M, Oliveira VC, et al. Drugs for relief of pain in patients with sciatica: systematic review and meta-analysis. Bmj [Internet]. 2012 Feb 13 [cited 2012 Mar 8];344(feb13 1):e497–e497.
47. Clarke CL, Ryan CG, Martin DJ. Pain neurophysiology education for the management of individuals with chronic low back pain: A systematic review and meta-analysis. Man Ther [Internet]. Elsevier Ltd; 2011;16(6):544–9.

Figure 1 POLAR CONSORT flowchart

Figure 2 Leg pain and ODI scores across groups

For peer review only

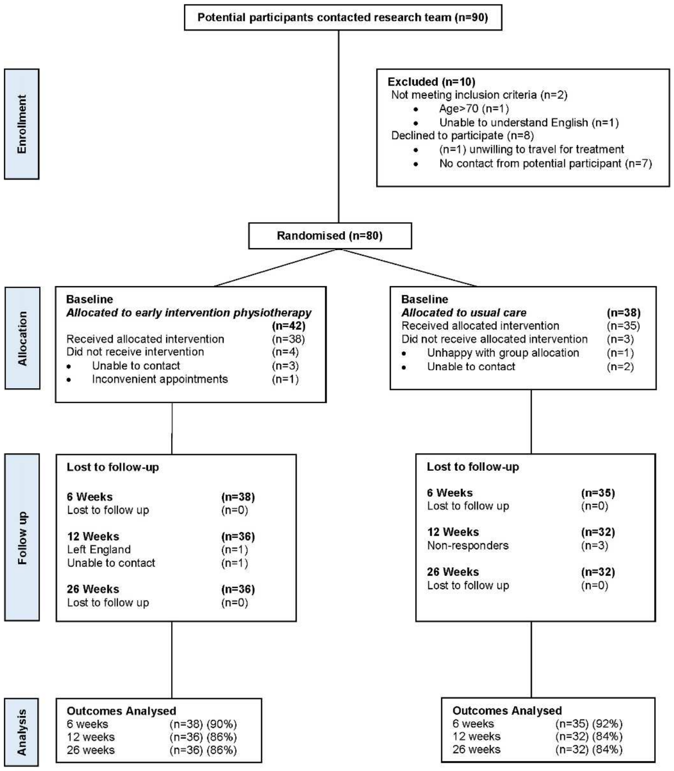

Figure 1 POLAR CONSORT flowchart

209x297mm (300 x 300 DPI)

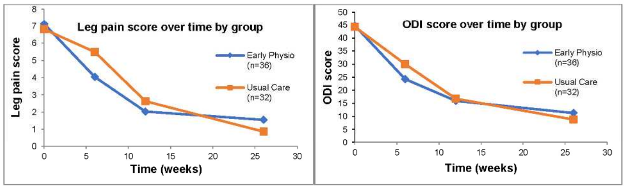

Figure 2 Leg pain and ODI scores across groups

209x297mm (300 x 300 DPI)

Does early intervention improve outcomes in the physiotherapy management of Lumbar Radicular Syndrome?

Logic Model

Intervention

1. Intervention components
a. Movement restriction
b. Neurological treatment
c. Treatment of Psychological barriers
d. Education regarding aetiology/prognosis of the problem
e. Conditioning for function
f. Movement control exercise
g. Pain control
h. Patient-centred-goal orientated care

2. Referral System change
a. Faxed referral
b. Designated slots for early intervention

3. Training
a. Specialist training for participating physiotherapists in diagnosis & management of LRS
b. Referral training for G.Ps

Short-term outcomes

1. Goal Achievement
- Individual, dynamic SMART goal achievement

2. Physical Factors
- Improved movement
- Improved neurological signs &/or symptoms
- Improved conditioning for function
- Improved uni or multi-planar neuro-muscular control
- Improved pain control
- Improve meaningful function

3. Psychological factors
- Decreased psychological barriers a to successful outcome
- Improved understanding of the problem(s)
- Increase confidence in movement & function

System Change
- Available capacity for appointments
- G.Ps able to refer for physiotherapy immediately
- Improved quality of referrals

Training
- Improved specialist knowledge & skills for physiotherapists

Moderating & Mediating Factors

Patient Factors
- Acceptability of the intervention
- Fear (kinesophobia & prognosis)
- Lack of understanding
- Perception of aetiology
- Perception of prognosis
- Perceived value of physiotherapy
- Availability of parking
- Age of patient
- Degree of severity

G.P factors
- Perception of physiotherapy & service provider
- Knowledge of LRS
- Fear (litigation-CES, complaint)
- Research burden
- Research interest

Physiotherapy factors
- Training & support

System-related factors
- Booking POLAR patients into correct appointments slots
- POLAR slot availability

Outcomes

Physiotherapists
- Job satisfaction
- Increased knowledge
- Increased confidence in treating patients with LRS

Patient
- Return to function
- Satisfaction with service
- Time to treatment

Potential Impact

Referral pathway

Service delivery efficiency

Cost effectiveness

Does early intervention improve outcomes in the physiotherapy management of Lumbar Radicular Syndrome? Logic Model V1 29.6.16

For peer review only

POLAR CONSORT 2010 checklist of information to include when reporting a pilot or feasibility trial*

Section/Topic	Item No	Checklist item	Reported on page No
**Title and abstract**			
	1a	Identification as a pilot or feasibility randomised trial in the title	1
	1b	Structured summary of pilot trial design, methods, results, and conclusions (for specific guidance see CONSORT abstract extension for pilot trials)	2,3
**Introduction**			
Background and objectives	2a	Scientific background and explanation of rationale for future definitive trial, and reasons for randomised pilot trial	4
	2b	Specific objectives or research questions for pilot trial	5
**Methods**			
Trial design	3a	Description of pilot trial design (such as parallel, factorial) including allocation ratio	5,6
	3b	Important changes to methods after pilot trial commencement (such as eligibility criteria), with reasons	5
Participants	4a	Eligibility criteria for participants	6
	4b	Settings and locations where the data were collected	5
	4c	How participants were identified and consented	6
Interventions	5	The interventions for each group with sufficient details to allow replication, including how and when they were actually administered	7
Outcomes	6a	Completely defined prespecified assessments or measurements to address each pilot trial objective specified in 2b, including how and when they were assessed	7, 8
	6b	Any changes to pilot trial assessments or measurements after the pilot trial commenced, with reasons	N/A
	6c	If applicable, prespecified criteria used to judge whether, or how, to proceed with future definitive trial	5
Sample size	7a	Rationale for numbers in the pilot trial	8
	7b	When applicable, explanation of any interim analyses and stopping guidelines	N/A
Randomisation:			
Sequence generation	8a	Method used to generate the random allocation sequence	6
	8b	Type of randomisation(s); details of any restriction (such as blocking and block size)	6
Allocation concealment mechanism	9	Mechanism used to implement the random allocation sequence (such as sequentially numbered containers), describing any steps taken to conceal the sequence until interventions were assigned	6

For peer review only

Implementation	10	Who generated the random allocation sequence, who enrolled participants, and who assigned participants to interventions	6
Blinding	11a	If done, who was blinded after assignment to interventions (for example, participants, care providers, those assessing outcomes) and how	N/A
	11b	If relevant, description of the similarity of interventions	7
Statistical methods	12	Methods used to address each pilot trial objective whether qualitative or quantitative	8
**Results**			
Participant flow (a diagram is strongly recommended)	13a	For each group, the numbers of participants who were approached and/or assessed for eligibility, randomly assigned, received intended treatment, and were assessed for each objective	9
	13b	For each group, losses and exclusions after randomisation, together with reasons	9
Recruitment	14a	Dates defining the periods of recruitment and follow-up	10
	14b	Why the pilot trial ended or was stopped	10
Baseline data	15	A table showing baseline demographic and clinical characteristics for each group	10
Numbers analysed	16	For each objective, number of participants (denominator) included in each analysis. If relevant, these numbers should be by randomised group	10,11,12
Outcomes and estimation	17	For each objective, results including expressions of uncertainty (such as 95% confidence interval) for any estimates. If relevant, these results should be by randomised group	11,12
Ancillary analyses	18	Results of any other analyses performed that could be used to inform the future definitive trial	
Harms	19	All important harms or unintended effects in each group (for specific guidance see CONSORT for harms)	12,13
	19a	If relevant, other important unintended consequences	N/A
**Discussion**			
Limitations	20	Pilot trial limitations, addressing sources of potential bias and remaining uncertainty about feasibility	18
Generalisability	21	Generalisability (applicability) of pilot trial methods and findings to future definitive trial and other studies	18
Interpretation	22	Interpretation consistent with pilot trial objectives and findings, balancing potential benefits and harms, and considering other relevant evidence	18
	22a	Implications for progression from pilot to future definitive trial, including any proposed amendments	18, 19
**Other information**			
Registration	23	Registration number for pilot trial and name of trial registry	19
Protocol	24	Where the pilot trial protocol can be accessed, if available	3
Funding	25	Sources of funding and other support (such as supply of drugs), role of funders	20
	26	Ethical approval or approval by research review committee, confirmed with reference number	19

For peer review only

Citation: Eldridge SM, Chan CL, Campbell MJ, Bond CM, Hopewell S, Thabane L, et al. CONSORT 2010 statement: extension to randomised pilot and feasibility trials. BMJ. 2016;355.

*We strongly recommend reading this statement in conjunction with the CONSORT 2010, extension to randomised pilot and feasibility trials, Explanation and Elaboration for important clarifications on all the items. If relevant, we also recommend reading CONSORT extensions for cluster randomised trials, non-inferiority and equivalence trials, non-pharmacological treatments, herbal interventions, and pragmatic trials. Additional extensions are forthcoming: for those and for up to date references relevant to this checklist, see www.consort-statement.org.

For peer review only

BMJ Open

Does early intervention improve outcomes in the physiotherapy management of Lumbar Radicular Syndrome? Results of the POLAR Pilot Randomised Controlled Trial

Journal:	*BMJ Open*
Manuscript ID	bmjopen-2018-021631.R2
Article Type:	Research
Date Submitted by the Author:	30-Apr-2018
Complete List of Authors:	Reddington, Michael; Sheffield Teaching Hospitals NHS Foundation Trust, Therapy Services<br>Walters, Stephen; University of Sheffield, ScHARR<br>Cohen, Judith; Hull York Medical School, Clinical Trials Research Unit<br>Baxter, Susan; University of Sheffield, ScHARR<br>Cole, Ashley; Sheffield Teaching Hospitals NHS Foundation Trust, Orthopaedic
**Primary Subject Heading**:	Rehabilitation medicine
Secondary Subject Heading:	Qualitative research, Research methods, Patient-centred medicine, General practice / Family practice
Keywords:	Back pain < ORTHOPAEDIC & TRAUMA SURGERY, Musculoskeletal disorders < ORTHOPAEDIC & TRAUMA SURGERY, Spine < ORTHOPAEDIC & TRAUMA SURGERY

SCHOLARONE™
Manuscripts

Does early intervention improve outcomes in the physiotherapy management of Lumbar Radicular Syndrome?
Results of the POLAR Pilot Randomised Controlled Trial

Corresponding author:

Michael Reddington
Therapy Services Outpatient Department
Sheffield Teaching Hospitals NHS Foundation Trust
Northern General Hospital
Herries Road
Sheffield S5 7AU
MReddington1@sheffield.ac.uk

Professor Stephen J Walters
Designs, Trials and Statistics
ScHARR, University of Sheffield
30 Regents Court
Sheffield S1 4DA
S.J.Walters@sheffield.ac.uk

Dr Judith Cohen
Hull Health Trials Unit
Hull York Medical School, University of Hull
Allam Medical Building, Cottingham Road
Hull HU6 7RX
J.Cohen@hull.ac.uk

Dr Susan Baxter
Section of Public Health
ScHARR, University of Sheffield
30 Regents Court
Sheffield S1 4DA
s.k.baxter@sheffield.ac.uk

Mr Ashley Cole
Consultant Orthopaedic Spinal Surgeon
Sheffield Teaching Hospitals NHS Foundation Trust
Northern General Hospital
Herries Road
Sheffield S5 7AU
Ashley.Cole@sth.nhs.uk

For peer review only

Abstract

Objective

To investigate the feasibility of undertaking a definitive Randomised Controlled Trial (RCT).

Setting

This was a pilot, pragmatic superiority RCT with a qualitative element, recruiting from 14 General Practitioner (G.P) practices in England.

Participants

Patients over 18 presenting to their G.P with unilateral Lumbar Radicular Syndrome (LRS) defined as radicular pain and/or neurological symptoms originating from lumbar nerve roots, were eligible to participate in the study, those who did not have a clear understanding of the English language or had co-morbidities preventing rehabilitation were ineligible.

Interventions

Participants were randomised into early intervention physiotherapy or usual care with the former receiving their treatment within 2 weeks after randomisation and the latter 6 weeks post randomisation. Both groups received a patient-centred, goal orientated physiotherapy programme specific to their needs. Participants received up to 6 treatment sessions over an 8-week period.

Outcome measures

Process outcomes to determine the feasibility of the study and an exploratory analysis of patient reported outcomes including self-rated disability, pain and general health, these were collected at baseline, 6, 12 and 26 weeks post randomisation.

Results

80 participants were recruited in 10 G.P practices over 34 weeks and randomised to (early intervention physiotherapy n= 42, usual care n=38). Follow-up rates at 26 weeks were 32 (84%) in the usual care and 36 (86%) in the early intervention physiotherapy group. The mean area under the curve (larger values indicating more disability) for the Oswestry Disability Index (ODI) over the 26 weeks was 16.6 (SD 11.4) in the Usual care group and 16.0 (SD 14.0), in the intervention group. A difference of -0.6 (95% CI: -0.68 to 5.6) in favour of the intervention group.

For peer review only

Conclusions

The results of the study suggest a full RCT is feasible and will provide evidence as to the optimal timing of physiotherapy for patients with LRS.

Trial Registration number ISRCTN: 25018352

Strengths and limitations of the study

- This pilot RCT was conducted in the usual care setting with clinical staff delivering the intervention.
- All feasibility objectives were met, including recruitment and participant attrition, and so the study can directly inform the design and conduct of a definitive RCT.
- Participants self-referred into the study after an introduction from their G.P (a pre-requisite for ethics approval) and so this group of patients may not be representative of a wider population.
- The diagnosis of LRS was made from the clinical history and examination and as such it is likely that there was a degree of diagnostic heterogeneity within the study sample.
- This was a pilot RCT and as such all analyses are exploratory.

For peer review only

Protocol

The protocol for the POLAR study was published and can be accessed at:

http://bmjopen.bmj.com/content/bmjopen/7/3/e014422.full.pdf

Introduction

Lumbar Radicular Syndrome (LRS) is a painful and disabling condition, usually of benign causation and in around 90% of cases associated with an intervertebral disc prolapse [1]. Symptomatic presentation of LRS is heterogenous, it can be self-limiting, lasting only a short time with no significant sequelae or can be a major cause of prolonged disability, work loss and long-term healthcare usage with associated costs [2-3]. Lifetime prevalence of LRS is estimated to be between 1% and 43% [4] with an annual incidence of between 1% and 5% [5].

Around 75% of LRS sufferers will have symptom resolution by 12 weeks, alongside spontaneous resorption of the Inter Vertebral Disc (IVD) [6]. However, there is no reliable predictor of early, late or no recovery at all [7]. Treatment guidelines encourage initial conservative management before considering surgery. Physiotherapy for LRS is commonly employed in the United Kingdom (U.K) for the management of LRS however, there is a lack of consensus on the type, duration and timing of the physiotherapy intervention [8]. Early intervention physiotherapy for Low Back Pain (LBP) has been found to improve patient outcomes, satisfaction and have lower healthcare usage and associated costs [9–11]. Delayed initiation of physiotherapy has been found to increase healthcare consumption in patients with LRS [12]. This suggests early treatment is important in terms of cost-savings and prevention of chronic symptom development [13] as increased symptom duration leads to worse outcomes for patients who undertake both conservative or surgical care [14-15] . Surgery for patients with LRS has been advocated, with optimum timing being between 4 weeks and 6 months after symptom onset [16-17]. Superiority studies of surgery and conservative management show a quicker improvement of patient symptoms in surgical groups, with results at a year showing no significant differences [18-19]. A significant number of patients never have any substantial relief from surgery with unsatisfactory outcomes in over 20% of patients at 5 years [20–21]. The timing of physiotherapy engagement for LRS has yet to be investigated.

Aims and Objectives

The study aim was to investigate the feasibility of undertaking a full Randomised Controlled Trial (RCT) to determine the effectiveness and cost-effectiveness of early intervention physiotherapy for patients with LRS.

For peer review only

Process Objectives

1. Successfully set-up recruitment sites in G.P practices.
2. Achieve a recruitment rate of 7 participants per month.
3. Demonstrate the ability to organise 75% of physiotherapy appointments within 2 weeks of randomisation.
4. Provide an appointment within 20 days of randomisation for >75% of participants randomised to the intervention group.
5. Achieve a participant attendance at >66% of physiotherapy appointments.
6. Achieve a participant attrition rate of <25% over the course of the study.
7. Achieve 80% return of Patient Reported Outcome Measures (PROMS) at 6/52 follow-up.

Research objectives

1. To test the feasibility, practicality, safety and acceptability of the study design and protocol.

2. Demonstrate acceptability of the primary and secondary outcome measures to patients and clinicians.

3. To inform the sample size calculation for the definitive RCT.

Methods

Design and setting

This was a mixed methods study comprising of an external pilot RCT with an embedded qualitative component in the form of stakeholder interviews in 14 G.P practices in a large city in England. Known as the POLAR study, the pilot RCT will be presented in this paper. A change was made to the inclusion criteria after 1 week of recruitment, the upper age limit of 70 was removed as this excluded a number of potential participants. The protocol for the study has been published, including extensive details of methods [22].

Patient and Public Involvement

The research question was informed directly from patient feedback on physiotherapy services. Current and past patients who have experienced LRS were involved from the inception to the end of the study in various ways. Firstly, they were involved in developing the research question, iteration of

For peer review only

the intervention and the study processes. They were invaluable in developing patient information and insight into recruitment strategies. Finally, they were actively involved in the interpretation of the results and discussions of the next stage of the study. Results will be distributed by email or post to participants who opted to receive the results at consent.

Randomisation

Information from the baseline dataset was used to randomise the participants using a web-based system. The Oswestry Disability Index 2.0 (ODI) [23] was used as the stratification factor with 3 levels based on ODI severity [24]; 'mild & moderate' (≤22-40%), 'severe' (>40 to 60%) and 'crippled' (>60 to 80%). A blinded block size was used to minimise predictability. The random allocation sequence and block size, stratified by centre and ODI disability score was independently generated by the Sheffield Clinical Trials Research Unit (CTRU).

Participants were informed of their group allocation within 1 working day of their consent and randomisation. Participants were randomised to treatment at either 2 or 6 weeks post-randomisation, we were unable to blind either patients or clinicians to the treatment allocation as it was obvious at what time-point they were receiving treatment. In an effort to minimise bias, both groups of patients received protocolised treatment based on the same assessment and treatment framework at the different time points.

Participants

Potential participants with a clinical diagnosis of LRS were identified by their G.P and given details of the study. Each participating G.P underwent training and were equipped with a diagnostic aide memoire for clinically identifying patients with LRS (See supplementary file 1). If interested, the patient contacted a member of the research team who screened for eligibility and arranged to meet to discuss the study. Anyone over the age of 18 years with unilateral LRS and who could speak English were eligible. If they had 'red flag' signs or symptoms such as cancer, cauda equine syndrome, spinal fracture or had other physical or psychological disabilities preventing rehabilitation, they were ineligible.

Recruitment & consent

For peer review only - http://bmjopen.bmj.com/site/about/guidelines.xhtml

Written consent was obtained by the research team after meeting the potential participant and confirming eligibility criteria including the clinical diagnosis of LRS. There were three recruitment cycles, each lasting up to 12 weeks or until 27 participants had been recruited for that cycle (26 for the final cycle). The remaining eight weeks were used for completion of treatment. A two-week period between cycles provided time to reflect and analyse the results from the stakeholder interviews and other feedback to refine the study processes as necessary.

The Intervention

The intervention was protocolised and allowed the treating physiotherapist a range of treatment options within each domain. Selected options were recorded electronically for each treatment session. The goal-orientated physiotherapy regimen for both groups were tailored to the individuals' requirements based on the information gathered from the baseline interview data, PROMS and clinical assessment. Participants were assessed using a multi-dimensional approach based on seven different elements; psychological barriers to recovery, neurological factors, movement restriction, understanding, conditioning, movement control and pain. Individualised physiotherapy for LBP and LRS is known to be superior and more cost-effective than advice alone [25,26], it is flexible and directly relevant to the individual and their changing needs. Participants received a maximum of six sessions of physiotherapy over an eight-week period, fewer if their pre-determined goals had been achieved. A logic model has been developed for the intervention which can be found as supplementary file 2.

Treatment Fidelity

Several strategies were employed to optimise fidelity, including a protocolised training package for the treating physiotherapists, standardised patient information, weekly feedback and support of treating physiotherapists and video analysis of each participating physiotherapist treating a study participant. The study took place in an NHS community setting using three physiotherapists, already employed by the host service provider. The physiotherapists had a mean age of 36 years (range 34-40 years) and a mean of 10 years postgraduate experience (range 7-12 years). They underwent 21 hours of training in the assessment and intervention and to promote and facilitate self-management, optimal function, pacing advice, analgesic advice together with equipping the patient with coping strategies.

For peer review only

Outcomes

Patients were asked to complete self-report and screening measures by post or face to face at four-time points: firstly, at the time of consent and then at 6, 12 and 26 weeks post randomisation**.** The primary outcomes for the study were process outcomes as the objective was to determine the feasibility of carrying out a full-scale RCT. Secondary outcomes were the ODI, Visual Analogue Scale (VAS) for back and leg pain, Keele STarT Back score [27], EQ5D-5L [28] and a self-report form focussing on functional loss, goals and medical history.

Sample size

It has been recommended that an external pilot study should have at least 70 measured participants (35 per group) when estimating the standard deviation for a continuous outcome [29]. A sample size of 80 patients, with approximately 10% allowance for loss to follow-up allows the standard deviation of an outcome to be estimated to within a precision of approximately ±16% of its true underlying value with 95% confidence.

Results

The flow chart of the participant journey for the POLAR study can be viewed in Figure 1. Ninety potential participants who were given details of the study by their respective G.Ps contacted the research team. Ten were excluded as they either did not meet the inclusion criteria or refused to be randomised, with 80 going on to be randomised from 10 different primary care G.P practices.

Baseline characteristics

The baseline characteristics of all participants, by group can be found in Table 1. This illustrates the comparability of the 2 arms with no evidence of selection bias. The groups were well matched for demographic factors such as age, gender and BMI as well as levels of disability, pain in leg and back, risk of chronicity and general health status. However, there was evidence of a difference in the EQ-5D utility scores which is attributable to chance as all participants were randomised. The early intervention physiotherapy group had longer symptom duration going into the study.

For peer review only

Table 1 Baseline characteristics of POLAR participants

	Early Intervention physiotherapy			Usual Care			Total		
	N	%		N	%		N	%	
**Female**	21	50		18	47		39	49	
**White British**	38	90		33	87		71	89	
	**N**	**Mean**	**SD**	**N**	**Mean**	**SD**	**N**	**Mean**	**SD**
**Age** (years)	42	47	14	38	47	13	80	47	13
**Height (**CM)	42	172.1	10.7	38	172.1	9.8	80	171.7	10.2
**Weight** (KG)	39[1]	81.5	14.8	38	80.6	15.7	77	81	15.2
**BMI**	39[1]	27.7	4.6	38	27.3	5.6	77	27.5	5.1
**ODI score** (%)	42	44.6	19.5	38	45.2	17.4	80	44.9	18.4
**Leg Pain**	42	7.2	1.8	38	6.9	2.3	80	7	2.1
**Back pain**	42	5.4	3.3	38	6	2.6	80	5.7	3.0
**EQ5D-5L** VAS	42	63.8	20.6	38	64.6	18.9	80	64.1	19.7
**EQ5D-5L** Utility score	42	0.44	0.29	38	0.52	0.25	80	0.48	0.27
**Keele STarT-Back**	42	5.7	2.0	38	5.7	1.8	80	5.7	1.9
**Keele STarT-Back** Sub-score	42	2.0	1.5	38	2.7	1.3	80	2.8	1.4
**Time to treatment** (days) [2]	38	11.1	10.5	31	43.6	8.9	69	25.7	19.0
	**N**	**Median**	**IQR**	**N**	**Median**	**IQR**	**N**	**Median**	**IQR**
**Symptoms duration** (days)	42	92	276	38	61	51	80	77	203

[1] 3 missing values

[2] Time between randomisation and first scheduled treatment session

Process Results

The POLAR study is a pilot trial and outlined below are the results of the feasibility objectives.

Set-up of recruitment sites in primary care

Twenty G.P practices were initially approached to take part in the study, with ten agreeing to participate. Towards the end of the second tranche of recruitment it was evident that one practice was recruiting a large number of participants and a decision was made to enrol new recruitment centres. Seven further G.P practices were therefore approached, with four agreeing to participate.

Recruitment rate

Eighty participants were recruited between the period 1st March 2016 and 7th November 2016 with a recruitment rate of 2.4 participants per week or 9.6 participants per month which enabled recruitment to end earlier than anticipated. Forty-two participants were randomised into the early intervention group and 38 in the usual care group.

Organisation of physiotherapy appointments

The target of 75% of physiotherapy appointments being made within two weeks of randomisation was surpassed in both groups. 100% (42/42) (95% CI: 92% to 100%) of early intervention physiotherapy participants received their appointment within 20 days of randomisation and 38/38 (95% CI:91% to 100%) in the usual care group. This illustrates the feasibility of making appointments for participants at short notice.

The feasibility of intervention delivery

A key feasibility parameter was the ability for at least 75% of early intervention physiotherapy participants to be seen by a physiotherapist, within 20 days of randomisation. 100% (42/42) (95% CI 92% to 100%) of participants reached this target, with a mean of 14.1 days between randomisation and first treatment session.

Participant treatment session attendance

The mean attendance rate for physiotherapy appointments in both groups was 92.6% (SD 16.2), 93.8% (SD 12.6) for the intervention group physiotherapy and 91.1% (SD 19.8) in the usual care group. All surpassed the a priori target of greater than 66% attendance. The mean number of treatment sessions received by the intervention group was 4 (SD=1) and 3 in the usual care group (SD=2).

Participant attrition

Eighty participants agreed to take part in the study. The intervention group attrition rate was 14% (6/42) (95% CI: 7% to 28%) and in the usual care group it was 16% (6/38) (95% CI 7% to 30%) at 26 weeks follow-up. The overall attrition rate for drop out of participants was 15% (95% CI 9% to 24%), all within the a priori limit set at 25%.

Outcome measure return

The outcome measure return rates surpassed expectations of 80% at six weeks and were as follows: 38/42 (91%; 95% CI: 78% to 96%) at six weeks post randomisation for the intervention group and 35/38 (92%; 95% CI 79% to 97%) for the usual care group.

For peer review only

Research results

Analysis of key clinical outcomes

Figure 2 shows the leg pain and ODI scores (likely primary outcome measures for definitive RCT) for participants with all 4 assessments completed. The blue line illustrates the increased rate of recovery in the early intervention physiotherapy group up to 6 weeks. When the usual care group begins their physiotherapy the rate of recovery assimilates and by 12 weeks and both groups have very similar scores. The descriptive statistics for all participants by group and time point can be found in Table 2. Two participants underwent lumbar micro-discectomy surgery for their LRS. Both participants had completed their respective courses of physiotherapy before undergoing surgery. S05/005 (usual care) failed to make significant improvements to their pain and with a severe level of pain and disability, surgery was undertaken. S06/027 (early intervention physiotherapy) had made significant improvements with physiotherapy, improving by over 20 points on the ODI, but required surgery due to ‘impending’ cauda equina syndrome.

The feasibility, practicality, safety and acceptability of the study design and protocol

The feasibility of the study has been suggested by the results of the feasibility parameters. There were several adjustments made to the processes of the study which were made possible by the breaks in recruitment. These included a brief weekly email to all participating G.Ps to remind them of the study and improve the clarity of inclusion and exclusion criteria. A change to the process of administering the six-week outcome measures was necessary, after the physiotherapists reported it too time consuming to administer. There were no changes made to the intervention, which appeared to be well received by both participants and clinicians alike. There were no adverse events or serious adverse events associated with the intervention or the study processes.

Harms

There was one Serious Adverse Event (SAE) during the course of the study in the early intervention physiotherapy group. The SAE rate was 2% (1/42) in the early intervention physiotherapy group and 0% (0/38) in the usual care group a difference of 2% (95% CI -7% to 12%). The participant was hospitalised after suffering a Cerebro-Vascular-Accident (CVA) related to pre-existing vascular hypertension. The participant had completed their physiotherapy intervention two weeks prior and

made a full recovery at 6 months. This was reported to the ethics committee and Trial Management Group (TMG).

Acceptability of the primary and secondary outcome measures to patients and clinicians

The importance of examining acceptability of the outcome measures, processes and the intervention was a key area of investigation for the study, and the pilot trial included a qualitative element to explore these aspects. Details of the qualitative aspects of the study will be reported in forthcoming papers. However, in summary the key processes necessary for implementation and evaluation of the study were reported to be acceptable by all stakeholders.

Fidelity

Physiotherapists recorded the components of their treatment sessions at each patient encounter in order to enhance and measure treatment fidelity. Participants in the early intervention physiotherapy group had a mean of 4 treatment sessions and those participants in the usual care group 3 sessions. There were 269 physiotherapy sessions carried out as part of the POLAR study with 1267 component parts (Table 3), 36 (3%) of which outside the protocolled treatment framework. The components outside the protocol consisted of three sessions of acupuncture and exercise other than that in the protocol. Video analysis was carried out independently on a purposive sample of 5 treatment sessions using a fidelity assessment tool developed by the lead author, clinical colleagues and PPIE representatives. The maximum score for 'essential' aspects of fidelity was 15/15. The median score for the videos was 14/15 (93%) with a range of 13-15 (87-100%).

Sample size calculation for the definitive RCT trial

For the definitive RCT we propose the primary outcome is the ODI at 26-weeks post-randomisation as the ODI has shown to be acceptable to patients and a commonly used measurement of self-rated disability. In this pilot trial, we observed a difference in means (in favour of the control group) of 2.5 points (95% CU: -4.5 to 9.1) between the randomised groups and a standard deviation of 16-points at 26 weeks. There is a lack of consensus regarding the Minimum Clinically Important Difference (MCID) for the ODI, with suggestions ranging from 6% to 30% [30,31]. Table 4 shows a range of sample sizes for varying target differences in the ODI. If we assume a target difference of five-points on the ODI scale, then with 217 patients per group (434 in total) we would have 90% power to detect a five-point

difference or more (equivalent to standardised effect size of 0.31) between the randomised groups which would be statistically significant at the 5% two-sided level. Allowing for a conservative estimate of 20% attrition (we observed 15% in this pilot) we would need to recruit and randomise 272 per group (544 in total).

Based on the recruitment rates observed in this trial of 80 patients in 8.5 months of recruitment at 10 centres (a rate of 0.9 patients per centre month); the main trial would need around 24 centres recruiting for 24 months to achieve this target.

For peer review only

Table 2 Descriptive statistics for outcome measures at each time point

Outcome	Baseline		6 Weeks		12 Weeks		26 weeks		Area under the response curve (AUC)				
											Difference 95% CI		
	Usual Care *n=38*	Early Intervention physiotherapy *n=42*	Usual Care *n=35*	Early Intervention physiotherapy *n=38*	Usual Care *n=32*	Early Intervention physiotherapy *n=36*	Usual Care *n=32*	Early Intervention physiotherapy *n=36*	**Usual Care *n=32***	**Early Intervention physiotherapy *n=36***	**Mean**	**Lower**	**Upper**
**ODI**[1](SD)	45.2(17.4)	44.6 (19.5)	29.1(16.1)	24.0(18.7)	16.8(19.2)	16.0(19.0)	8.8(11.3)	11.3(15.5)	**16.6 (11.4)**	**16.0 (14.0)**	**-0.6**	**-6.8**	**5.6**
**VAS Back**[2] (SD)	6.0(2.6)	5.4(3.3)	4.6(2.7)	3.7(2.6)	3.1(2.5)	2.6(2.5)	2.1(2.1)	2.7(2.2)	**1.8 (0.8)**	**1.5 (1.0)**	**-0.3**	**-0.7**	**0.1**
**VAS Leg**[2] (SD)	6.9(2.3)	7.2(1.8)	5.2(2.9)	4.1(3.0)	2.6(2.9)	2.0(2.5)	0.9(2.2)	1.6(2.2)	**1.7 (0.9)**	**1.5 (1.0)**	**-0.2**	**-0.6**	**0.3**
**EQ5D5L**[3]VAS (SD)	64.6(18.9)	63.8(20.6)	68.9(16.4	72.7(17.7)	73.2(22.9)	79.6(17.5)	81.7(12)	79.6(16.3)	**36.8 (7.1)**	**38.1 (7.8)**	**1.4**	**-2.2**	**5.0**
**EQ5D-5L**[4] Utility score (SD)	0.52(0.25)	0.44(0.29)	0.7(0.26)	0.74(0.22)	0.83(0.23)	0.85(0.22)	0.92(0.12)	0.86(0.19)	**0.39 (0.09)**	**0.39 (0.10)**	**0.00**	**-0.05**	**0.04**

[1]Oswestry Disability Index (ODI) 0-100, higher score=higher level of self-rated disability. For the ODI a larger AUC represents a greater level of disability over the 26 weeks. A negative difference means the Early Intervention Physiotherapy group has the better outcome (lower levels of disability) over the 26-weeks follow-up.

[2]Visual Analogue Scale 0-10, higher score=higher self-report pain. For the VAS back pain and leg pain outcomes a larger AUC represents a higher level of pain over the 26 weeks. A negative difference means the Early Intervention Physiotherapy group has the better outcome (lower levels of pain) over the 26-weeks follow-up.

[3] EQ5D-5L VAS score, 0-100, self-rated health. the higher the score, the better the quality of life. For the EQ5D-5L VAS score a larger AUC represents a higher level of quality of life over the 26 weeks. A positive difference means the Early Intervention Physiotherapy group has the better outcome (higher levels of quality of life) over the 26-weeks follow-up.

[4] EQ5D-5L Utility score, -0.6 to 1.00 with a higher score representing better quality of life. For the EQ5D-5L Utility score a larger AUC represents a higher level of quality of life over the 26 weeks. A positive difference means the Early Intervention Physiotherapy group has the better outcome (higher levels of quality of life) over the 26-weeks follow-up.

For peer review only - http://bmjopen.bmj.com/site/about/guidelines.xhtml

Table 3 Intervention domains and components frequency table

Domain	No. of participants receiving component N=69	Method of assessment	Treatment options	Frequency of component used	%
**Psychological barriers to recovery** [32-34]	47 (68%)	Keele STarTBack Clinical interview & history	a. Treatment of Kinesiophobia with graded exposure, education and movement re-education	16	1.3
			b. Treatment of hypervigilance with education, distraction & desensitisation	17	1.4
			c. Treatment of faulty beliefs about pain, LRS, treatment and/or prognosis with education and self-management strategies	38	3.2
			d. Treatment of Iatrogenic beliefs and corresponding avoidance behaviours with education and movement re-education	3	0.2
			e. Treatment of aspects of work as a barrier to recovery and treatment with ergonomic advice and practise	15	1.2
			f. Identification of financial barriers to recovery and signposting e.g. debt management	15	1.2
			g. Identification of emotional barriers to recovery and signposting to appropriate therapy e.g. G.P/Psychology	57	4.7
**Neurological** [35–38]	39 (58%)	Clinical assessment	a. Neural interface mobilisation	98	8.1
			b. Functional neurological movement re-education	7	0.6
**Movement restriction** [39]	59 (86%)	Clinical assessment	a. Flexion mobilisation (Grade 2-4)	68	5.6
			b. Side-flexion mobilisation (Gr. 2-4)	5	0.4
			c. Extension mobilisation (Gr. 2-4)	15	1.2
			d. Rotation mobilisation (Gr. 2-4)	41	3.4
			e. Flexion+Side-flexion mobilisation (Gr. 2-4)	11	0.9
			f. Flexion+Side+flexion+rotation mobilisation (Gr. 2-4)	62	5.2
			g. Extension+Side flexion mobilisation (Grade 2-4)	0	0
			h. Manipulation (Gr. 5)	0	0
			i. Seated Mobilisation With Movement (MWM)	16	1.3
			j. Standing MWM	16	1.3
			k. Mobilisation into functional position	14	1.2

For peer review only - http://bmjopen.bmj.com/site/about/guidelines.xhtml

			l.	Muscle stretches	61	5.1
			m.	Functional movement re-education	7	0.6
**Understanding** [40]	66 (96%)		a.	Management of erroneous believes relating to LRS provide education to help eradicate these beliefs	57	4.7
			b.	Pacing behaviours	53	4.4
			c.	Goal attainment	58	4.8
			d.	Health Promotion	80	6.6
			e.	Identification and treatment of central sensitisation-liaison with G.P/pain clinic	8	0.7
			f.	Identification and treatment of peripheral sensitisation-liaison with G.P/pain clinic	7	0.6
**Conditioning** [41,42]	63 (91%)	Self-assessment answers, clinical interview & history	a.	Cardiovascular & conditioning exercise relevant to patients' goals	83	6.9
			b.	Function specific stretches	39	3.2
			c.	Function specific strengthening	62	5.2
			d.	Ergonomic advice	14	1.2
			e.	Ergonomic practise	6	0.5
			f.	Group exercise	0	0.0
			g.	Perturbation training	7	0.6
**Movement control** [43]	33 (48%)	Clinical assessment	a.	Sagittal plane control in functional positions relevant to patients' problems/goals	24	2.0
			b.	Coronal plane control in functional positions relevant to patients' problems/goals	15	1.2
			c.	Axial plane control in functional positions relevant to patients' problems/goals	1	0.1
			d.	Multi-planar control in functional positions relevant to patients' problems/goals	6	0.5
			e.	Movement re-education in functional positions relevant to patients' problems/goals	18	1.5
**Pain** [44–46]	52 (75%)	ODI VAS back & leg Clinical interview & history	a.	Analgesic review & advice in liaison with G.P/Pharmacist	23	1.9
			b.	Pain education	60	5.0
			c.	Pain coping strategies	20	1.7
			d.	Fear reduction intervention in liaison with psychologist/pain clinic	12	1.0
			e.	Stress reduction intervention in liaison with psychologist/pain clinic	32	2.7
**Totals**					1267	99.8%[1]

[1] 0.2% missing data-2 treatment episodes where components not attributed.

For peer review only

For peer review only - http://bmjopen.bmj.com/site/about/guidelines.xhtml

Table 4 Sample sizes for main RCT for a range of target mean differences with a primary outcome of the ODI score at 26-weeks post-randomisation

Significance Level	Power	SD	Target Mean Difference	Standardised Effect Size	Number in each group	Total Sample Size (N)	Total sample size Dropout	
							15%	20%
5%	90%	16	2	0.13	1346	2692	3168	3366
5%	90%	16	2.5	0.16	862	1724	2030	2156
5%	90%	16	3	0.19	599	1198	1410	1498
5%	90%	16	3.5	0.22	441	882	1038	1104
5%	90%	16	4	0.25	338	676	796	846
5%	90%	16	4.5	0.28	267	534	630	668
5%	90%	16	5	0.31	217	434	512	544
5%	90%	16	5.5	0.34	179	358	422	448
5%	90%	16	6	0.38	151	302	356	378
5%	90%	16	6.5	0.41	129	258	304	324
5%	90%	16	7	0.44	111	222	262	278
5%	90%	16	7.5	0.47	97	194	230	244
5%	90%	16	8	0.50	86	172	204	216
5%	90%	16	8.5	0.53	76	152	180	190
5%	90%	16	9	0.56	68	136	160	170
5%	90%	16	9.5	0.59	61	122	144	154
5%	90%	16	10	0.63	55	110	130	138

For peer review only

Discussion

This pilot study is the first to explore the role of early intervention physiotherapy for LRS. The study aimed to determine the feasibility of carrying out a full-scale RCT to determine the effectiveness of early physiotherapy for LRS. All of the feasibility parameters were found to be acceptable, including the set-up of G.P centres to recruit participants, recruitment of participants and the retention of 85% of participants at 26 weeks. Both groups received the intervention at the appropriate time, within 2 weeks of randomisation for the early intervention physiotherapy group and after 6 weeks for the usual care group. The acceptance of the intervention, judged by the rate of attendance by participants at their treatment sessions, was better than anticipated.

There were some limitations to this study. Firstly, although recruitment was satisfactory and ahead of time, the G.Ps involved in the study were well motivated and supportive of the study, in a city with a proven track-record of G.P involvement in service development and research. This may not be the case across the country and further afield. Similarly, the support of the service provider clinical, administrative and management staff was a key factor in the success of the study, a factor which may not be reproducible in other centres. Patients self-referred into the study after an introduction from their G.P (a pre-requisite for ethics approval) and so this group of patients may not be representative of a wider population. These factors need to be taken in account when planning a definitive study, and we have taken a more conservative view of attrition in the definitive sample size calculation. Our recommendations about recruitment also suggest including a wider geographical spread of G.P centres to help meet the proposed recruitment rates. Site selection would need to consider current service provision and the ability to deliver the intervention in settings that are convenient and accessible to patients. The reliance on a clinical diagnosis of LRS made by the G.P and physiotherapists is a potential limitation. The limitation being that there is likely to be a degree of diagnostic heterogeneity within the sample using a patho-anatomical model of care. There is therefore potential that participants with LRS in the study may have symptoms from something other than nerve root inflammation, including pseud-radicular symptoms, somatic or visceral referred symptoms.

The strengths of the study are that it was a pragmatic study in a clinical setting, using clinical staff and available resources and as such represents the real world of the NHS. We demonstrated that the study is feasible and the potential of early intervention physiotherapy to improve patient care.

For peer review only - http://bmjopen.bmj.com/site/about/guidelines.xhtml

Conclusion

The POLAR study results indicate that a full-scale trial of early physiotherapy to treat patients with LRS is feasible. As there is a dearth of evidence about how and when best to treat this population, we conclude that a definitive trial is needed to help inform clinical practice.

Other information

Ethical review

Ethical approval was received from NHS Scotland, East of Scotland Research Ethics Service (EoSRES) in August 2015 (REC reference 15/ES/0130). The study was conducted in accordance with the declaration of Helsinki and local governance requirements.

Trial Registration

ISRCTN: 25018352

Clinical Trials.Gov: NCT02618278

Acknowledgments

The authors would like to thank the members of the Patient, Public Involvement and Engagement (PPIE) group (Paul Colton, Amanda Dempsey, Rachel Harrison and Claire Village) for their invaluable support and guidance. Thank-you to Dr Jon Dickson, Mark Rhymes and Mark Pinkerton of the Yorkshire and Humber CRN. Thank-you to the wonderful local physiotherapy service for their support, especially the fantastic physiotherapists who delivered the intervention and management (Sarah Withers & Helen Wilson). Finally, and most importantly a massive thank-you to the participants.

Contributors

MR – Instigated the idea for the study, developed the funding proposal and applied for funding. He developed the protocol, intervention handbook, gained ethical approval and acted as CI for the study.

SW – Is the primary PhD supervisor for MRs' fellowship and contributed to the study conception, design, and writing of the protocol and provided guidance with the statistical analysis.

JC – Is an academic supervisor for MR and has provided specific guidance on protocol development, regulatory approvals and the design of the study.

For peer review only

SB – Provided input regarding the qualitative and mixed method design and analysis aspects of the study.

AAC- Provides clinical supervision for M.R. He has been involved in the conception of the study, its organisation, analysis and writing.

All authors read and commented on drafts and approved the final version of the manuscript.

Competing interests

MR–None

SW-None

JC-None

SB-None

AAC-None

All authors have completed the Unified Competing Interest form at www.icmje.org/coi_disclosure.pdf (available on request from the corresponding author) and declare (1) No financial support for the submitted work from anyone other than their employer; (2) No financial relationships with commercial entities that might have an interest in the submitted work; (3) No spouses, partners, or children with relationships with commercial entities that might have an interest in the submitted work; (4) No non-financial interests that may be relevant to the submitted work.

Funding

The lead author (MR) has received a personal Clinical Doctoral Research Fellowship (CDRF) award from Health Education England (HEE) and the National Institute of Health Research (NIHR). Award number: CDRF-2014-05-046.

Disclaimer

This paper presents independent research funded by the National Institute for Health Research (NIHR). The views expressed are those of the author(s) and not necessarily those of the NHS, the NIHR or the Department of Health

Data sharing statement

For peer review only

There are no additional data available for the study which has not been published. All data is available to anyone interested by contacting the corresponding author.

For peer review only

References

1. Koes, Æ Bart W, van Tulder MW PW. The diagnosis and treatment of sciatica. Br Med J. 2007;334:1313–7.
2. Waddell G. The Back Pain Revolution. 2nd editio. Churchill Livingstone, Edinburgh; 2004.
3. Hoy D, March L, Brooks P, Blyth F, Woolf A, Bain C, et al. The global burden of low back pain: estimates from the Global Burden of Disease 2010 study. Ann Rheum Dis [Internet]. 2014;73(6):968–74.
4. Konstantinou K, Dunn KM. Sciatica Review of Epidemiological Studies and Prevalence Estimates. Spine (Phila Pa 1976). 2008;33(22):2464–72.
5. NICE. Low back pain and sciatica in over 16s: assessment and management [Internet]. Https://Www.Nice.Org.Uk/Guidance/Ng59. 2016.
6. Vroomen PC a J, de Krom MCTFM, Knottnerus J a. Predicting the outcome of sciatica at short-term follow-up. Br J Gen Pract [Internet]. 2002 Feb;52(475):119–23.
7. Suri P, Carlson MJ, Rainville J. Nonoperative Treatment for Lumbosacral Radiculopathy: What Factors Predict Treatment Failure? Clin Orthop Relat Res [Internet]. Springer US; 2014;1931–9.
8. Thomas KC, Fisher CG, Boyd M, Bishop P, Wing P, Dvorak MF. Outcome Evaluation of Surgical and Nonsurgical Management of Lumbar Disc Protrusion Causing Radiculopathy. Spine (Phila Pa 1976). 2007;32(13):1414–22.
9. Lau PM-Y, Chow DH-K, Pope MH. Early physiotherapy intervention in an Accident and Emergency Department reduces pain and improves satisfaction for patients with acute low back pain: a randomised trial. Aust J Physiother [Internet]. 2008 Jan;54(4):243–9.
10. Wand BM, Bird C, Mcauley JH, Dore CJ, Macdowell M, Souza LH De. Early Intervention for the Management of Acute Low Back Pain Education , Manual Therapy , and Exercise. Spine (Phila Pa 1976). 2004;29(21):2350–6.
11. Whitfill T, Haggard R, Bierner SM, Pransky G, Hassett RG, Gatchel RJ. Early intervention options for acute low back pain patients: A randomized clinical trial with one-year follow-up outcomes. J Occup Rehabil. 2010;20(2):256–63.
12. Fritz JM, Childs JD, Wainner RS, Flynn TW. Primary care referral of patients with low back pain to physical therapy: impact on future health care utilization and costs. Spine (Phila Pa 1976) [Internet]. 2012 Dec 1 [cited 2014 Jan 31];37(25):2114–21.
13. Gellhorn AC, Chan L, Martin B, Friedly J. Management patterns in acute low back pain: the role of physical therapy. Spine (Phila Pa 1976) [Internet]. 2012 Apr 20 [cited 2014 Feb 3];37(9):775–82.
14. Rihn JA, Hilibrand AS, Radcliff K, Kurd M, Lurie J, Blood E, et al. Duration of Symptoms Resulting from Lumbar Disc Herniation: Effect on Treatment Outcomes. J Bone Jt Surg [Internet]. 2011;93–A(20):1906–14.
15. Weinstein JN, Lurie JD, Tosteson TD, Tosteson AN a, Blood E a, Abdu W a, et al. Surgical versus nonoperative treatment for lumbar disc herniation: four-year results for the Spine Patient Outcomes Research Trial (SPORT). Spine (Phila Pa 1976) [Internet]. 2008 Dec;33(25):2789–800.
16. Jacobs WCH, van Tulder M, Arts M, Rubinstein SM, van Middelkoop M, Ostelo R, et al. Surgery versus conservative management of sciatica due to a lumbar herniated disc: a systematic review. Eur Spine J [Internet]. 2011;20(4):513–22.
17. Peul WC, Arts MP, Brand R, Koes BW. Timing of surgery for sciatica: subgroup analysis alongside a randomized trial. Eur Spine J [Internet]. 2009;18(4):538–45.
18. Peul WC, Brand R, Thomeer RTWM, Koes BW. Improving prediction of “inevitable” surgery during non-surgical treatment of sciatica. Pain. 2008;138(3):571–6.
19. Alentado VJ, Mroz TE, Lubelski D, Steinmetz MP, Benzel EC. Optimal Duration of Conservative Management Prior to Surgery for Cervical and Lumbar Radiculopathy : A Literature Review. Glob Spine J. 2014;279–86.
20. Machado GC., Witzleb AJ., Fritsch C., Maher CG., Ferreira PH., Ferreira ML. d. Patients with sciatica still experience pain and disability 5 years after surgery: A systematic review with meta-analysis of cohort studies. Eur J Pain (United Kingdom) [Internet]. 2016;20(March):1–8.
21. Lequin MB, Verbaan D, Jacobs WCH, Brand R, Bouma GJ, Vandertop WP, et al. Surgery versus prolonged conservative treatment for sciatica: 5-year results of a randomised controlled trial. BMJ Open [Internet]. 2013 Jan [cited 2013 Jul 15];3(5):1–10.
22. Reddington M, Walters SJ, Cohen J, Baxter S. Does early intervention improve outcomes in physiotherapy management of lumbar radicular syndrome? A mixed-methods study protocol.

For peer review only

BMJ Open [Internet]. 2017;7(3):e014422.
23. Fairbank JC, Pynsent PB. The Oswestry Disability Index. Spine (Phila Pa 1976) [Internet]. 2000 Nov 15;25(22):2940–52; discussion 2952.
24. Fairbank, JC. Couper, J. Davies, JB. O'Brien J. The Oswestry low back pain disability questionnaire. Physiother. 1980;66(8):271–3.
25. Hahne AJ, Ford JJ, Hinman RS, Richards MC, Surkitt LD, Chan AYP, et al. Individualized functional restoration as an adjunct to advice for lumbar disc herniation with associated radiculopathy. A preplanned subgroup analysis of a randomized controlled trial. Spine J. Elsevier Inc.; 2017;17(3):346–59.
26. Hahne AJ, Ford JJ, Surkitt LD, Richards MC, Chan AYP, Slater SL, et al. Individualized Physical Therapy Is Cost-Effective Compared With Guideline-Based Advice for People With Low Back Disorders. Spine (Phila Pa 1976) [Internet]. 2017;42(3):E169–76.
27. Hill JC, Dunn KM, Lewis M, Mullis R, Main CJ, Foster NE, et al. A primary care back pain screening tool: identifying patient subgroups for initial treatment. Arthritis Rheum [Internet]. 2008;59(5):632–41.
28. EuroQol Group. EuroQol--a new facility for the measurement of health-related quality of life. Health Policy [Internet]. 1990;16(3):199–208.
29. Teare MD, Dimairo M, Shephard N, Hayman A, Whitehead A, Walters SJ. Sample size requirements to estimate key design parameters from external pilot randomised controlled trials: a simulation study. Trials [Internet]. 2014;15(1):264.
30. Fritz JM, Irrgang J.J. A Comparison of a Modified Oswestry Low Back Pain Disability Questionnaire and the Quebec Back Pain Disability Scale. Phys Ther. 2001;81(2):776–88.
31. Ostelo RWJG, Deyo RA, Stratford P, Waddell G, Croft P, Korff M Von, et al. Interpreting Change Scores for Pain and Functional Status in Low Back Pain. Spine (Phila Pa 1976). 2008;33(1):90–4.
32. Nicholas MK, George SZ. Psychologically informed interventions for low back pain: an update for physical therapists. Phys Ther [Internet]. 2011 May [cited 2012 Nov 22];91(5):765–76.
33. Blyth FM, Macfarlane GJ, Nicholas MK. The contribution of psychosocial factors to the development of chronic pain: the key to better outcomes for patients? Pain [Internet]. 2007;129(1–2):8–11.
34. Linton SJ, Shaw WS. Impact of psychological factors in the experience of pain. Phys Ther [Internet]. 2011 May [cited 2012 Oct 28];91(5):700–11.
35. Coppieters MW, Butler DS. Do "sliders" slide and "tensioners" tension? An analysis of neurodynamic techniques and considerations regarding their application. Man Ther. 2008;13(3):213–21.
36. Basson A, Olivier B, Ellis R, Coppieters M, Stewart A, Mudzi W. The Effectiveness of Neural Mobilization for Neuro-Musculoskeletal Conditions: A Systematic Review and Meta-Analysis. J Orthop Sport Phys Ther [Internet]. 2017;47(1401):1–76.
37. Efstathiou MA, Stefanakis M, Savva C, Giakas G. Effectiveness of neural mobilization inpatients with spinal radiculopathy: Acritical review. J Bodyw Mov Ther [Internet]. Elsevier Ltd; 2015;19(2):205–12.
38. Butler D. The Sensitive Nervous System. 1st ed. NOI Group Publications; 2000.
39. Hengeveld E BK. Maitlands' Vertebral Manipulation. 8th ed. Churchill Livingstone, Edinburgh; 2013.
40. Otoo, S.K.W, Hendrick, P, Ribeiro D. The comparative effectiveness of advice/education compared to active physiotherapy (manual therapy and exercise) in the management of chronic non-specific low back pain. Phys Ther Rev. 2015;20(1):16–26.
41. Searle A., Spink M, Ho A, Chuter V. Exercise interventions for the treatment of chronic low back pain: A systematic review and meta-analysis of randomised controlled trials. Clin Rehabil [Internet]. 2015;12:1155–67.
42. Falla D, Whiteley R, Marco C, Hodges P. Therapeutic Exercise. In: Jull G, Moore A, Falla DL, Lewis J, McCarthy C, Sterling M, editors. Grieves Modern Musculoskeletal Physiotherapy. 4th ed. 2015. p. 298–310.
43. O'Sullivan P. Diagnosis and classification of chronic low back pain disorders: maladaptive movement and motor control impairments as underlying mechanism. Man Ther [Internet]. 2005;10(4):242–55.
44. Lorig KR, Holman HR, Med AB. Self-Management Education : History , Definition , Outcomes , and Mechanisms. Ann Behav Med. 2003;26(1):1–7.
45. Pinto RZ, Maher CG, Ferreira ML, Ferreira PH, Hancock M, Oliveira VC, et al. Drugs for relief of pain in patients with sciatica: systematic review and meta-analysis. Bmj [Internet]. 2012 Feb

For peer review only

13 [cited 2012 Mar 8];344(feb13 1):e497–e497.
46. Clarke CL, Ryan CG, Martin DJ. Pain neurophysiology education for the management of individuals with chronic low back pain: A systematic review and meta-analysis. Man Ther [Internet]. Elsevier Ltd; 2011;16(6):544–9.

Figure 1 POLAR CONSORT flowchart

Figure 2 Leg pain and ODI scores across groups

For peer review only

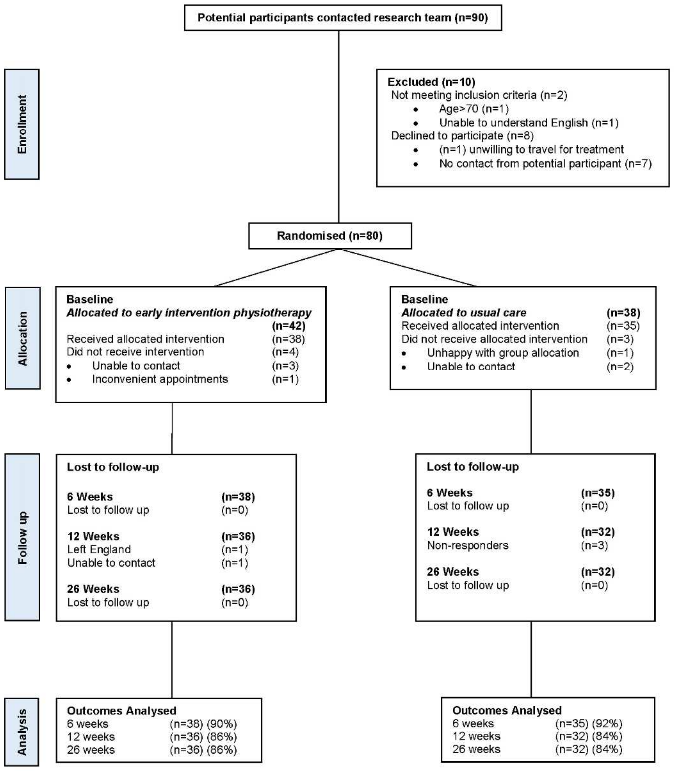

Figure 1 POLAR CONSORT flowchart

209x297mm (300 x 300 DPI)

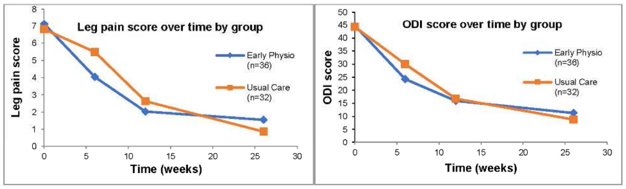

Figure 2 Leg pain and ODI scores across groups

209x297mm (300 x 300 DPI)

POLAR
Physiotherapy for Sciatica Study

PhysiOtherapy Management of LumbAr Radicular Syndrome

Does Early Intervention Improve Outcomes

Patient Recruitment Flow

G.P sees Patient with LRS

Ineligible

Eligible

Give Patient Information Sheet

Put Date on sheet

G.P refers patient to PhysioWorks

Fax to: 0114 3051698

Eligibility Criteria

Must be & have:

1. Unilateral symptoms of **sciatica** or **femoral** radicular pain
(See over for definitions)

Exclusion Criteria

Must **not** have:

1. 'Red flag' signs or symptoms
2. Cancer at the time of the study
3. Proven vascular claudication
4. Bilateral signs/symptoms
5. Spinal fracture within the last 3 months
6. Poor English skills (necessitating the use of an interpreter)

For peer review only - http://bmjopen.bmj.com/site/about/guidelines.xhtml

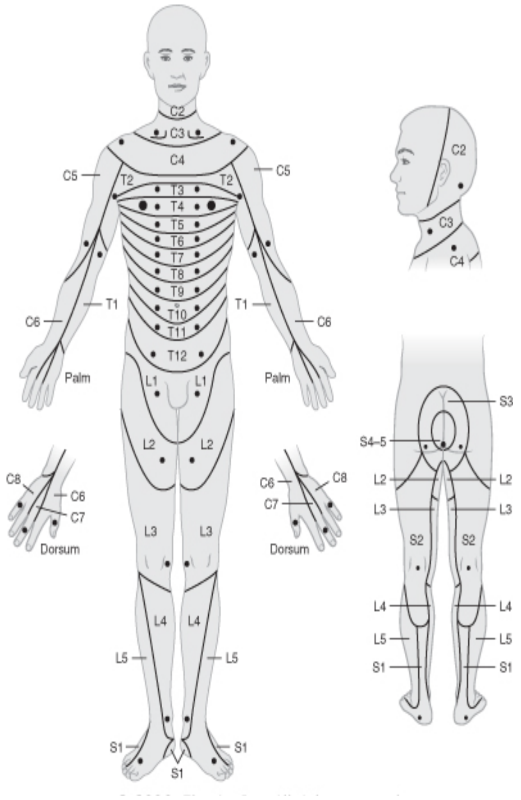

Characteristics of Sciatica (L4,5,S1,S2)

- Pain and/or paraesthesia and/or numbness in a L4-S2 dermatomal distribution.
- Symptoms usually buttock, posterior or lateral thigh and anterior/posterior/lateral leg.
- Shooting, burning or lancinating type pain.
- Symptoms extend below the knee.

Characteristics of Femoral radicular pain (L1,2,3)

- Anterior/Antero-lateral thigh pain and/or paraesthesia and/or numbness in L1-3 dermatomal distribution.
- Shooting, burning or lancinating type pain.
- Symptoms extend above or to the knee, not below the knee.

Red Flag Signs and Symptoms

Red flag signs or symptoms are those which arose suspicion of potential serious or life-threatening pathology. There are hundreds of red flags in the literature, most of which of spurious validity. However a few useful ones in relation to low back pain and sciatica are:

- Previous cancer
- Systemically unwell (? Potential discitis)
- Spinal deformity (? fracture)
- Significant, unexplained weight loss
- Severe, unremitting pain
- I.V drug use (infection-?discitis)
- Cauda equina syndrome
- Upper motor neurone signs or symptoms (loss of dexterity, worsening balance, gait disturbance, falls, positive UMN tests-hyperreflexia, positive Romberg sign, positive Hoffman sign, muscular hypertonicity etc)

This list is of course not exhaustive and services only to highlight those patients who require further investigation rather than physiotherapy.

For peer review only

For peer review only - http://bmjopen.bmj.com/site/about/guidelines.xhtml

Does early intervention improve outcomes in the physiotherapy management of Lumbar Radicular Syndrome?

Logic Model

Intervention

1. Intervention components

a. Movement restriction
b. Neurological treatment
c. Treatment of Psychological barriers
d. Education regarding aetiology/prognosis of the problem
e. Conditioning for function
f. Movement control exercise
g. Pain control
h. Patient-centred-goal orientated care

2. Referral System change

a. Faxed referral
b. Designated slots for early intervention

3. Training

a. Specialist training for participating physiotherapists in diagnosis & management of LRS
b. Referral training for G.Ps

Short-term outcomes

1. Goal Achievement

- Individual, dynamic SMART goal achievement

2. Physical Factors

- Improved movement
- Improved neurological signs &/or symptoms
- Improved conditioning for function
- Improved uni or multi-planar neuro-muscular control
- Improved pain control
- Improve meaningful function

3. Psychological factors

- Decreased psychological barriers a to successful outcome
- Improved understanding of the problem(s)
- Increase confidence in movement & function

System Change

- Available capacity for appointments
- G.Ps able to refer for physiotherapy immediately
- Improved quality of referrals

Training

- Improved specialist knowledge & skills for physiotherapists

Moderating & Mediating Factors

Patient Factors

- Acceptability of the intervention
- Fear (kinesophobia & prognosis)
- Lack of understanding
- Perception of aetiology
- Perception of prognosis
- Perceived value of physiotherapy
- Availability of parking
- Age of patient
- Degree of severity

G.P factors

- Perception of physiotherapy & service provider
- Knowledge of LRS
- Fear (litigation-CES, complaint)
- Research burden
- Research interest

Physiotherapy factors

- Training & support

System-related factors

- Booking POLAR patients into correct appointments slots
- POLAR slot availability

Outcomes

Physiotherapists

- Job satisfaction
- Increased knowledge
- Increased confidence in treating patients with LRS

Patient

- Return to function
- Satisfaction with service
- Time to treatment

Potential Impact

Referral pathway

Service delivery efficiency

Cost effectiveness

For peer review only

Does early intervention improve outcomes in the physiotherapy management of Lumbar Radicular Syndrome? Logic Model V1 29.6.16

POLAR CONSORT 2010 checklist of information to include when reporting a pilot or feasibility trial*

Section/Topic	Item No	Checklist item	Reported on page No
**Title and abstract**			
	1a	Identification as a pilot or feasibility randomised trial in the title	1
	1b	Structured summary of pilot trial design, methods, results, and conclusions (for specific guidance see CONSORT abstract extension for pilot trials)	2,3
**Introduction**			
Background and objectives	2a	Scientific background and explanation of rationale for future definitive trial, and reasons for randomised pilot trial	4
	2b	Specific objectives or research questions for pilot trial	5
**Methods**			
Trial design	3a	Description of pilot trial design (such as parallel, factorial) including allocation ratio	5,6
	3b	Important changes to methods after pilot trial commencement (such as eligibility criteria), with reasons	5
Participants	4a	Eligibility criteria for participants	6
	4b	Settings and locations where the data were collected	5
	4c	How participants were identified and consented	6
Interventions	5	The interventions for each group with sufficient details to allow replication, including how and when they were actually administered	7
Outcomes	6a	Completely defined prespecified assessments or measurements to address each pilot trial objective specified in 2b, including how and when they were assessed	7, 8
	6b	Any changes to pilot trial assessments or measurements after the pilot trial commenced, with reasons	N/A
	6c	If applicable, prespecified criteria used to judge whether, or how, to proceed with future definitive trial	5
Sample size	7a	Rationale for numbers in the pilot trial	8
	7b	When applicable, explanation of any interim analyses and stopping guidelines	N/A
Randomisation:			
Sequence generation	8a	Method used to generate the random allocation sequence	6
	8b	Type of randomisation(s); details of any restriction (such as blocking and block size)	6
Allocation concealment mechanism	9	Mechanism used to implement the random allocation sequence (such as sequentially numbered containers), describing any steps taken to conceal the sequence until interventions were assigned	6

For peer review only

Implementation	10	Who generated the random allocation sequence, who enrolled participants, and who assigned participants to interventions	6
Blinding	11a	If done, who was blinded after assignment to interventions (for example, participants, care providers, those assessing outcomes) and how	N/A
	11b	If relevant, description of the similarity of interventions	7
Statistical methods	12	Methods used to address each pilot trial objective whether qualitative or quantitative	8
**Results**			
Participant flow (a diagram is strongly recommended)	13a	For each group, the numbers of participants who were approached and/or assessed for eligibility, randomly assigned, received intended treatment, and were assessed for each objective	9
	13b	For each group, losses and exclusions after randomisation, together with reasons	9
Recruitment	14a	Dates defining the periods of recruitment and follow-up	10
	14b	Why the pilot trial ended or was stopped	10
Baseline data	15	A table showing baseline demographic and clinical characteristics for each group	10
Numbers analysed	16	For each objective, number of participants (denominator) included in each analysis. If relevant, these numbers should be by randomised group	10,11,12
Outcomes and estimation	17	For each objective, results including expressions of uncertainty (such as 95% confidence interval) for any estimates. If relevant, these results should be by randomised group	11,12
Ancillary analyses	18	Results of any other analyses performed that could be used to inform the future definitive trial	
Harms	19	All important harms or unintended effects in each group (for specific guidance see CONSORT for harms)	12,13
	19a	If relevant, other important unintended consequences	N/A
**Discussion**			
Limitations	20	Pilot trial limitations, addressing sources of potential bias and remaining uncertainty about feasibility	18
Generalisability	21	Generalisability (applicability) of pilot trial methods and findings to future definitive trial and other studies	18
Interpretation	22	Interpretation consistent with pilot trial objectives and findings, balancing potential benefits and harms, and considering other relevant evidence	18
	22a	Implications for progression from pilot to future definitive trial, including any proposed amendments	18, 19
**Other information**			
Registration	23	Registration number for pilot trial and name of trial registry	19
Protocol	24	Where the pilot trial protocol can be accessed, if available	3
Funding	25	Sources of funding and other support (such as supply of drugs), role of funders	20
	26	Ethical approval or approval by research review committee, confirmed with reference number	19

For peer review only

Citation: Eldridge SM, Chan CL, Campbell MJ, Bond CM, Hopewell S, Thabane L, et al. CONSORT 2010 statement: extension to randomised pilot and feasibility trials. BMJ. 2016;355.
*We strongly recommend reading this statement in conjunction with the CONSORT 2010, extension to randomised pilot and feasibility trials, Explanation and Elaboration for important clarifications on all the items. If relevant, we also recommend reading CONSORT extensions for cluster randomised trials, non-inferiority and equivalence trials, non-pharmacological treatments, herbal interventions, and pragmatic trials. Additional extensions are forthcoming: for those and for up to date references relevant to this checklist, see www.consort-statement.org.

For peer review only

BMJ Open

Does early intervention improve outcomes in the physiotherapy management of Lumbar Radicular Syndrome? Results of the POLAR Pilot Randomised Controlled Trial

Journal:	*BMJ Open*
Manuscript ID	bmjopen-2018-021631.R3
Article Type:	Research
Date Submitted by the Author:	17-May-2018
Complete List of Authors:	Reddington, Michael; Sheffield Teaching Hospitals NHS Foundation Trust, Therapy Services<br>Walters, Stephen; University of Sheffield, ScHARR<br>Cohen, Judith; Hull York Medical School, Clinical Trials Research Unit<br>Baxter, Susan; University of Sheffield, ScHARR<br>Cole, Ashley; Sheffield Teaching Hospitals NHS Foundation Trust, Orthopaedic
**Primary Subject Heading**:	Rehabilitation medicine
Secondary Subject Heading:	Qualitative research, Research methods, Patient-centred medicine, General practice / Family practice
Keywords:	Back pain < ORTHOPAEDIC & TRAUMA SURGERY, Musculoskeletal disorders < ORTHOPAEDIC & TRAUMA SURGERY, Spine < ORTHOPAEDIC & TRAUMA SURGERY

SCHOLARONE™
Manuscripts

only

Does early intervention improve outcomes in the physiotherapy management of Lumbar Radicular Syndrome? Results of the POLAR Pilot Randomised Controlled Trial

Corresponding author:

Michael Reddington
Therapy Services Outpatient Department
Sheffield Teaching Hospitals NHS Foundation Trust
Northern General Hospital
Herries Road
Sheffield S5 7AU
MReddington1@sheffield.ac.uk

Professor Stephen J Walters
Designs, Trials and Statistics
ScHARR, University of Sheffield
30 Regents Court
Sheffield S1 4DA
S.J.Walters@sheffield.ac.uk

Dr Judith Cohen
Hull Health Trials Unit
Hull York Medical School, University of Hull
Allam Medical Building, Cottingham Road
Hull HU6 7RX
J.Cohen@hull.ac.uk

Dr Susan Baxter
Section of Public Health
ScHARR, University of Sheffield
30 Regents Court
Sheffield S1 4DA
s.k.baxter@sheffield.ac.uk

Mr Ashley Cole
Consultant Orthopaedic Spinal Surgeon
Sheffield Teaching Hospitals NHS Foundation Trust
Northern General Hospital
Herries Road
Sheffield S5 7AU
Ashley.Cole@sth.nhs.uk

For peer review only

Abstract

Objective

To investigate the feasibility of undertaking a definitive Randomised Controlled Trial (RCT).

Setting

This was a pilot, pragmatic superiority RCT with a qualitative element, recruiting from 14 General Practitioner (G.P) practices in England.

Participants

Patients over 18 presenting to their G.P with unilateral Lumbar Radicular Syndrome (LRS) defined as radicular pain and/or neurological symptoms originating from lumbar nerve roots, were eligible to participate in the study, those who did not have a clear understanding of the English language or had co-morbidities preventing rehabilitation were ineligible.

Interventions

Participants were randomised into early intervention physiotherapy or usual care with the former receiving their treatment within 2 weeks after randomisation and the latter 6 weeks post randomisation. Both groups received a patient-centred, goal orientated physiotherapy programme specific to their needs. Participants received up to 6 treatment sessions over an 8-week period.

Outcome measures

Process outcomes to determine the feasibility of the study and an exploratory analysis of patient reported outcomes including self-rated disability, pain and general health, these were collected at baseline, 6, 12 and 26 weeks post randomisation.

Results

80 participants were recruited in 10 G.P practices over 34 weeks and randomised to (early intervention physiotherapy n= 42, usual care n=38). Follow-up rates at 26 weeks were 32 (84%) in the usual care and 36 (86%) in the early intervention physiotherapy group. The mean area under the curve (larger values indicating more disability) for the Oswestry Disability Index (ODI) over the 26 weeks was 16.6 (SD 11.4) in the Usual care group and 16.0 (SD 14.0), in the intervention group. A difference of -0.6 (95% CI: -0.68 to 5.6) in favour of the intervention group.

For peer review only

Conclusions

The results of the study suggest a full RCT is feasible and will provide evidence as to the optimal timing of physiotherapy for patients with LRS.

Trial Registration number ISRCTN: 25018352

Strengths and limitations of the study

- This pilot RCT was conducted in the usual care setting with clinical staff delivering the intervention.
- All feasibility objectives were met, including recruitment and participant attrition, and so the study can directly inform the design and conduct of a definitive RCT.
- Participants self-referred into the study after an introduction from their G.P (a pre-requisite for ethics approval) and so this group of patients may not be representative of a wider population.
- The diagnosis of LRS was made from the clinical history and participant symptomatology and as such it is likely that there was a degree of diagnostic heterogeneity within the study sample.
- This was a pilot RCT and as such all analyses are exploratory.

Protocol

The protocol for the POLAR study was published and can be accessed at:

http://bmjopen.bmj.com/content/bmjopen/7/3/e014422.full.pdf

For peer review only

Introduction

Lumbar Radicular Syndrome (LRS) is a painful and disabling condition, usually of benign causation and in around 90% of cases associated with an intervertebral disc prolapse [1]. Symptomatic presentation of LRS is heterogenous, it can be self-limiting, lasting only a short time with no significant sequelae or can be a major cause of prolonged disability, work loss and long-term healthcare usage with associated costs [2-3]. Lifetime prevalence of LRS is estimated to be between 1% and 43% [4] with an annual incidence of between 1% and 5% [5].

Around 75% of LRS sufferers will have symptom resolution by 12 weeks, alongside spontaneous resorption of the Inter Vertebral Disc (IVD) [6]. However, there is no reliable predictor of early, late or no recovery at all [7]. Treatment guidelines encourage initial conservative management before considering surgery. Physiotherapy for LRS is commonly employed in the United Kingdom (U.K) for the management of LRS however, there is a lack of consensus on the type, duration and timing of the physiotherapy intervention [8]. Early intervention physiotherapy for Low Back Pain (LBP) has been found to improve patient outcomes, satisfaction and have lower healthcare usage and associated costs [9–11]. Delayed initiation of physiotherapy has been found to increase healthcare consumption in patients with LRS [12]. This suggests early treatment is important in terms of cost-savings and prevention of chronic symptom development [13] as increased symptom duration leads to worse outcomes for patients who undertake both conservative or surgical care [14-15] . Surgery for patients with LRS has been advocated, with optimum timing being between 4 weeks and 6 months after symptom onset [16-17]. Superiority studies of surgery and conservative management show a quicker improvement of patient symptoms in surgical groups, with results at a year showing no significant differences [18-19]. A significant number of patients never have any substantial relief from surgery with unsatisfactory outcomes in over 20% of patients at 5 years [20–21]. The timing of physiotherapy engagement for LRS has yet to be investigated.

Aims and Objectives

The study aim was to investigate the feasibility of undertaking a full Randomised Controlled Trial (RCT) to determine the effectiveness and cost-effectiveness of early intervention physiotherapy for patients with LRS.

For peer review only - http://bmjopen.bmj.com/site/about/guidelines.xhtml

Process Objectives

1. Successfully set-up recruitment sites in G.P practices.
2. Achieve a recruitment rate of 7 participants per month.
3. Demonstrate the ability to organise 75% of physiotherapy appointments within 2 weeks of randomisation.
4. Provide an appointment within 20 days of randomisation for >75% of participants randomised to the intervention group.
5. Achieve a participant attendance at >66% of physiotherapy appointments.
6. Achieve a participant attrition rate of <25% over the course of the study.
7. Achieve 80% return of Patient Reported Outcome Measures (PROMS) at 6/52 follow-up.

Research objectives

1. To test the feasibility, practicality, safety and acceptability of the study design and protocol.

2. Demonstrate acceptability of the primary and secondary outcome measures to patients and clinicians.

3. To inform the sample size calculation for the definitive RCT.

Methods

Design and setting

This was a mixed methods study comprising of an external pilot RCT with an embedded qualitative component in the form of stakeholder interviews in 14 G.P practices in a large city in England. Known as the POLAR study, the pilot RCT will be presented in this paper. A change was made to the inclusion criteria after 1 week of recruitment, the upper age limit of 70 was removed as this excluded a number of potential participants. The protocol for the study has been published, including extensive details of methods [22].

Patient and Public Involvement

The research question was informed directly from patient feedback on physiotherapy services. Current and past patients who have experienced LRS were involved from the inception to the end of the study in various ways. Firstly, they were involved in developing the research question, iteration of

For peer review only

the intervention and the study processes. They were invaluable in developing patient information and insight into recruitment strategies. Finally, they were actively involved in the interpretation of the results and discussions of the next stage of the study. Results will be distributed by email or post to participants who opted to receive the results at consent.

Randomisation

Information from the baseline dataset was used to randomise the participants using a web-based system. The Oswestry Disability Index 2.0 (ODI) [23] was used as the stratification factor with 3 levels based on ODI severity [24]; 'mild & moderate' (≤22-40%), 'severe' (>40 to 60%) and 'crippled' (>60 to 80%). A blinded block size was used to minimise predictability. The random allocation sequence and block size, stratified by centre and ODI disability score was independently generated by the Sheffield Clinical Trials Research Unit (CTRU).

Participants were informed of their group allocation within 1 working day of their consent and randomisation. Participants were randomised to treatment at either 2 or 6 weeks post-randomisation, we were unable to blind either patients or clinicians to the treatment allocation as it was obvious at what time-point they were receiving treatment. In an effort to minimise bias, both groups of patients received protocolised treatment based on the same assessment and treatment framework at the different time points.

Participants

Potential participants with a clinical diagnosis of LRS were identified by their G.P and given details of the study. Each participating G.P underwent training and were equipped with a diagnostic aide memoire for clinically identifying patients with LRS (See supplementary file 1). If interested, the patient contacted a member of the research team who screened for eligibility and arranged to meet to discuss the study. Anyone over the age of 18 years with unilateral LRS and who could speak English were eligible. If they had 'red flag' signs or symptoms such as cancer, cauda equine syndrome, spinal fracture or had other physical or psychological disabilities preventing rehabilitation, they were ineligible.

Recruitment & consent

Written consent was obtained by the research team after meeting the potential participant and confirming eligibility criteria including the clinical diagnosis of LRS. There were three recruitment cycles, each lasting up to 12 weeks or until 27 participants had been recruited for that cycle (26 for the final cycle). The remaining eight weeks were used for completion of treatment. A two-week period between cycles provided time to reflect and analyse the results from the stakeholder interviews and other feedback to refine the study processes as necessary.

The Intervention

The intervention was protocolised and allowed the treating physiotherapist a range of treatment options within each domain. Selected options were recorded electronically for each treatment session. The goal-orientated physiotherapy regimen for both groups were tailored to the individuals' requirements based on the information gathered from the baseline interview data, PROMS and clinical assessment. Participants were assessed using a multi-dimensional approach based on seven different elements; psychological barriers to recovery, neurological factors, movement restriction, understanding, conditioning, movement control and pain. Individualised physiotherapy for LBP and LRS is known to be superior and more cost-effective than advice alone [25,26], it is flexible and directly relevant to the individual and their changing needs. Participants received a maximum of six sessions of physiotherapy over an eight-week period, fewer if their pre-determined goals had been achieved. A logic model has been developed for the intervention which can be found as supplementary file 2.

Treatment Fidelity

Several strategies were employed to optimise fidelity, including a protocolised training package for the treating physiotherapists, standardised patient information, weekly feedback and support of treating physiotherapists and video analysis of each participating physiotherapist treating a study participant. The study took place in an NHS community setting using three physiotherapists, already employed by the host service provider. The physiotherapists had a mean age of 36 years (range 34-40 years) and a mean of 10 years postgraduate experience (range 7-12 years). They underwent 21 hours of training in the assessment and intervention and to promote and facilitate self-management, optimal function, pacing advice, analgesic advice together with equipping the patient with coping strategies.

For peer review only

Outcomes

Patients were asked to complete self-report and screening measures by post or face to face at four-time points: firstly, at the time of consent and then at 6, 12 and 26 weeks post randomisation**.** The primary outcomes for the study were process outcomes as the objective was to determine the feasibility of carrying out a full-scale RCT. Secondary outcomes were the ODI, Visual Analogue Scale (VAS) for back and leg pain, Keele STarT Back score [27], EQ5D-5L [28] and a self-report form focussing on functional loss, goals and medical history.

Sample size

It has been recommended that an external pilot study should have at least 70 measured participants (35 per group) when estimating the standard deviation for a continuous outcome [29]. A sample size of 80 patients, with approximately 10% allowance for loss to follow-up allows the standard deviation of an outcome to be estimated to within a precision of approximately ±16% of its true underlying value with 95% confidence.

Results

The flow chart of the participant journey for the POLAR study can be viewed in Figure 1. Ninety potential participants who were given details of the study by their respective G.Ps contacted the research team. Ten were excluded as they either did not meet the inclusion criteria or refused to be randomised, with 80 going on to be randomised from 10 different primary care G.P practices.

Baseline characteristics

The baseline characteristics of all participants, by group can be found in Table 1. This illustrates the comparability of the 2 arms with no evidence of selection bias. The groups were well matched for demographic factors such as age, gender and BMI as well as levels of disability, pain in leg and back, risk of chronicity and general health status. However, there was evidence of a difference in the EQ-5D utility scores which is attributable to chance as all participants were randomised. The early intervention physiotherapy group had longer symptom duration going into the study.

For peer review only

Table 1 Baseline characteristics of POLAR participants

	Early Intervention physiotherapy			Usual Care			Total		
	N	%		N	%		N	%	
**Female**	21	50		18	47		39	49	
**White British**	38	90		33	87		71	89	
	**N**	**Mean**	**SD**	**N**	**Mean**	**SD**	**N**	**Mean**	**SD**
**Age** (years)	42	47	14	38	47	13	80	47	13
**Height (**CM)	42	172.1	10.7	38	172.1	9.8	80	171.7	10.2
**Weight** (KG)	39[1]	81.5	14.8	38	80.6	15.7	77	81	15.2
**BMI**	39[1]	27.7	4.6	38	27.3	5.6	77	27.5	5.1
**ODI score** (%)	42	44.6	19.5	38	45.2	17.4	80	44.9	18.4
**Leg Pain**	42	7.2	1.8	38	6.9	2.3	80	7	2.1
**Back pain**	42	5.4	3.3	38	6	2.6	80	5.7	3.0
**EQ5D-5L** VAS	42	63.8	20.6	38	64.6	18.9	80	64.1	19.7
**EQ5D-5L** Utility score	42	0.44	0.29	38	0.52	0.25	80	0.48	0.27
**Keele STarT-Back**	42	5.7	2.0	38	5.7	1.8	80	5.7	1.9
**Keele STarT-Back** Sub-score	42	2.0	1.5	38	2.7	1.3	80	2.8	1.4
**Time to treatment** (days)[2]	38	11.1	10.5	31	43.6	8.9	69	25.7	19.0
	**N**	**Median**	**IQR**	**N**	**Median**	**IQR**	**N**	**Median**	**IQR**
**Symptoms duration** (days)	42	92	276	38	61	51	80	77	203

[1] 3 missing values

[2] Time between randomisation and first scheduled treatment session

For peer review only

Process Results

The POLAR study is a pilot trial and outlined below are the results of the feasibility objectives.

Set-up of recruitment sites in primary care

Twenty G.P practices were initially approached to take part in the study, with ten agreeing to participate. Towards the end of the second tranche of recruitment it was evident that one practice was recruiting a large number of participants and a decision was made to enrol new recruitment centres. Seven further G.P practices were therefore approached, with four agreeing to participate.

Recruitment rate

Eighty participants were recruited between the period 1st March 2016 and 7th November 2016 with a recruitment rate of 2.4 participants per week or 9.6 participants per month which enabled recruitment to end earlier than anticipated. Forty-two participants were randomised into the early intervention group and 38 in the usual care group.

Organisation of physiotherapy appointments

The target of 75% of physiotherapy appointments being made within two weeks of randomisation was surpassed in both groups. 100% (42/42) (95% CI: 92% to 100%) of early intervention physiotherapy participants received their appointment within 20 days of randomisation and 38/38 (95% CI:91% to 100%) in the usual care group. This illustrates the feasibility of making appointments for participants at short notice.

The feasibility of intervention delivery

A key feasibility parameter was the ability for at least 75% of early intervention physiotherapy participants to be seen by a physiotherapist, within 20 days of randomisation. 100% (42/42) (95% CI 92% to 100%) of participants reached this target, with a mean of 14.1 days between randomisation and first treatment session.

Participant treatment session attendance

The mean attendance rate for physiotherapy appointments in both groups was 92.6% (SD 16.2), 93.8% (SD 12.6) for the intervention group physiotherapy and 91.1% (SD 19.8) in the usual care group. All surpassed the a priori target of greater than 66% attendance. The mean number of treatment sessions received by the intervention group was 4 (SD=1) and 3 in the usual care group (SD=2).

Participant attrition

Eighty participants agreed to take part in the study. The intervention group attrition rate was 14% (6/42) (95% CI: 7% to 28%) and in the usual care group it was 16% (6/38) (95% CI 7% to 30%) at 26 weeks follow-up. The overall attrition rate for drop out of participants was 15% (95% CI 9% to 24%), all within the a priori limit set at 25%.

Outcome measure return

The outcome measure return rates surpassed expectations of 80% at six weeks and were as follows: 38/42 (91%; 95% CI: 78% to 96%) at six weeks post randomisation for the intervention group and 35/38 (92%; 95% CI 79% to 97%) for the usual care group.

For peer review only - http://bmjopen.bmj.com/site/about/guidelines.xhtml

Research results

Analysis of key clinical outcomes

Figure 2 shows the leg pain and ODI scores (likely primary outcome measures for definitive RCT) for participants with all 4 assessments completed. The blue line illustrates the increased rate of recovery in the early intervention physiotherapy group up to 6 weeks. When the usual care group begins their physiotherapy the rate of recovery assimilates and by 12 weeks and both groups have very similar scores. The descriptive statistics for all participants by group and time point can be found in Table 2. Two participants underwent lumbar micro-discectomy surgery for their LRS. Both participants had completed their respective courses of physiotherapy before undergoing surgery. S05/005 (usual care) failed to make significant improvements to their pain and with a severe level of pain and disability, surgery was undertaken. S06/027 (early intervention physiotherapy) had made significant improvements with physiotherapy, improving by over 20 points on the ODI, but required surgery due to ‘impending’ cauda equina syndrome.

The feasibility, practicality, safety and acceptability of the study design and protocol

The feasibility of the study has been suggested by the results of the feasibility parameters. There were several adjustments made to the processes of the study which were made possible by the breaks in recruitment. These included a brief weekly email to all participating G.Ps to remind them of the study and improve the clarity of inclusion and exclusion criteria. A change to the process of administering the six-week outcome measures was necessary, after the physiotherapists reported it too time consuming to administer. There were no changes made to the intervention, which appeared to be well received by both participants and clinicians alike. There were no adverse events or serious adverse events associated with the intervention or the study processes.

Harms

There was one Serious Adverse Event (SAE) during the course of the study in the early intervention physiotherapy group. The SAE rate was 2% (1/42) in the early intervention physiotherapy group and 0% (0/38) in the usual care group a difference of 2% (95% CI -7% to 12%). The participant was hospitalised after suffering a Cerebro-Vascular-Accident (CVA) related to pre-existing vascular hypertension. The participant had completed their physiotherapy intervention two weeks prior and

made a full recovery at 6 months. This was reported to the ethics committee and Trial Management Group (TMG).

Acceptability of the primary and secondary outcome measures to patients and clinicians

The importance of examining acceptability of the outcome measures, processes and the intervention was a key area of investigation for the study, and the pilot trial included a qualitative element to explore these aspects. Details of the qualitative aspects of the study will be reported in forthcoming papers. However, in summary the key processes necessary for implementation and evaluation of the study were reported to be acceptable by all stakeholders.

Fidelity

Physiotherapists recorded the components of their treatment sessions at each patient encounter in order to enhance and measure treatment fidelity. Participants in the early intervention physiotherapy group had a mean of 4 treatment sessions and those participants in the usual care group 3 sessions. There were 269 physiotherapy sessions carried out as part of the POLAR study with 1267 component parts (Table 3), 36 (3%) of which outside the protocolled treatment framework. The components outside the protocol consisted of three sessions of acupuncture and exercise other than that in the protocol. Video analysis was carried out independently on a purposive sample of 5 treatment sessions using a fidelity assessment tool developed by the lead author, clinical colleagues and PPIE representatives. The maximum score for 'essential' aspects of fidelity was 15/15. The median score for the videos was 14/15 (93%) with a range of 13-15 (87-100%).

Sample size calculation for the definitive RCT trial

For the definitive RCT we propose the primary outcome is the ODI at 26-weeks post-randomisation as the ODI has shown to be acceptable to patients and a commonly used measurement of self-rated disability. In this pilot trial, we observed a difference in means (in favour of the control group) of 2.5 points (95% CU: -4.5 to 9.1) between the randomised groups and a standard deviation of 16-points at 26 weeks. There is a lack of consensus regarding the Minimum Clinically Important Difference (MCID) for the ODI, with suggestions ranging from 6% to 30% [30,31]. Table 4 shows a range of sample sizes for varying target differences in the ODI. If we assume a target difference of five-points on the ODI scale, then with 217 patients per group (434 in total) we would have 90% power to detect a five-point

For peer review only

difference or more (equivalent to standardised effect size of 0.31) between the randomised groups which would be statistically significant at the 5% two-sided level. Allowing for a conservative estimate of 20% attrition (we observed 15% in this pilot) we would need to recruit and randomise 272 per group (544 in total).

Based on the recruitment rates observed in this trial of 80 patients in 8.5 months of recruitment at 10 centres (a rate of 0.9 patients per centre month); the main trial would need around 24 centres recruiting for 24 months to achieve this target.

For peer review only

Table 2 Descriptive statistics for outcome measures at each time point

Outcome	Baseline		6 Weeks		12 Weeks		26 weeks		Area under the response curve (AUC)				
											Difference 95% CI		
	Usual Care *n=38*	Early Intervention physiotherapy *n=42*	Usual Care *n=35*	Early Intervention physiotherapy *n=38*	Usual Care *n=32*	Early Intervention physiotherapy *n=36*	Usual Care *n=32*	Early Intervention physiotherapy *n=36*	**Usual Care *n=32***	**Early Intervention physiotherapy *n=36***	**Mean**	**Lower**	**Upper**
**ODI**[1](SD)	45.2(17.4)	44.6 (19.5)	29.1(16.1)	24.0(18.7)	16.8(19.2)	16.0(19.0)	8.8(11.3)	11.3(15.5)	**16.6 (11.4)**	**16.0 (14.0)**	**-0.6**	**-6.8**	**5.6**
**VAS Back**[2] (SD)	6.0(2.6)	5.4(3.3)	4.6(2.7)	3.7(2.6)	3.1(2.5)	2.6(2.5)	2.1(2.1)	2.7(2.2)	**1.8 (0.8)**	**1.5 (1.0)**	**-0.3**	**-0.7**	**0.1**
**VAS Leg**[2] (SD)	6.9(2.3)	7.2(1.8)	5.2(2.9)	4.1(3.0)	2.6(2.9)	2.0(2.5)	0.9(2.2)	1.6(2.2)	**1.7 (0.9)**	**1.5 (1.0)**	**-0.2**	**-0.6**	**0.3**
**EQ5D5L**[3]VAS (SD)	64.6(18.9)	63.8(20.6)	68.9(16.4	72.7(17.7)	73.2(22.9)	79.6(17.5)	81.7(12)	79.6(16.3)	**36.8 (7.1)**	**38.1 (7.8)**	**1.4**	**-2.2**	**5.0**
**EQ5D-5L**[4] Utility score (SD)	0.52(0.25)	0.44(0.29)	0.7(0.26)	0.74(0.22)	0.83(0.23)	0.85(0.22)	0.92(0.12)	0.86(0.19)	**0.39 (0.09)**	**0.39 (0.10)**	**0.00**	**-0.05**	**0.04**

[1]Oswestry Disability Index (ODI) 0-100, higher score=higher level of self-rated disability. For the ODI a larger AUC represents a greater level of disability over the 26 weeks. A negative difference means the Early Intervention Physiotherapy group has the better outcome (lower levels of disability) over the 26-weeks follow-up.

[2]Visual Analogue Scale 0-10, higher score=higher self-report pain. For the VAS back pain and leg pain outcomes a larger AUC represents a higher level of pain over the 26 weeks. A negative difference means the Early Intervention Physiotherapy group has the better outcome (lower levels of pain) over the 26-weeks follow-up.

[3] EQ5D-5L VAS score, 0-100, self-rated health. the higher the score, the better the quality of life. For the EQ5D-5L VAS score a larger AUC represents a higher level of quality of life over the 26 weeks. A positive difference means the Early Intervention Physiotherapy group has the better outcome (higher levels of quality of life) over the 26-weeks follow-up.

[4] EQ5D-5L Utility score, -0.6 to 1.00 with a higher score representing better quality of life. For the EQ5D-5L Utility score a larger AUC represents a higher level of quality of life over the 26 weeks. A positive difference means the Early Intervention Physiotherapy group has the better outcome (higher levels of quality of life) over the 26-weeks follow-up.

For peer review only

For peer review only - http://bmjopen.bmj.com/site/about/guidelines.xhtml

Table 3 Intervention domains and components frequency table

Domain	No. of participants receiving component N=69	Method of assessment	Treatment options	Frequency of component used	%
**Psychological barriers to recovery** [32-34]	47 (68%)	Keele STarTBack Clinical interview & history	a. Treatment of Kinesiophobia with graded exposure, education and movement re-education	16	1.3
			b. Treatment of hypervigilance with education, distraction & desensitisation	17	1.4
			c. Treatment of faulty beliefs about pain, LRS, treatment and/or prognosis with education and self-management strategies	38	3.2
			d. Treatment of Iatrogenic beliefs and corresponding avoidance behaviours with education and movement re-education	3	0.2
			e. Treatment of aspects of work as a barrier to recovery and treatment with ergonomic advice and practise	15	1.2
			f. Identification of financial barriers to recovery and signposting e.g. debt management	15	1.2
			g. Identification of emotional barriers to recovery and signposting to appropriate therapy e.g. G.P/Psychology	57	4.7
**Neurological** [35–38]	39 (58%)	Clinical assessment	a. Neural interface mobilisation	98	8.1
			b. Functional neurological movement re-education	7	0.6
**Movement restriction** [39]	59 (86%)	Clinical assessment	a. Flexion mobilisation (Grade 2-4)	68	5.6
			b. Side-flexion mobilisation (Gr. 2-4)	5	0.4
			c. Extension mobilisation (Gr. 2-4)	15	1.2
			d. Rotation mobilisation (Gr. 2-4)	41	3.4
			e. Flexion+Side-flexion mobilisation (Gr. 2-4)	11	0.9
			f. Flexion+Side+flexion+rotation mobilisation (Gr. 2-4)	62	5.2
			g. Extension+Side flexion mobilisation (Grade 2-4)	0	0
			h. Manipulation (Gr. 5)	0	0
			i. Seated Mobilisation With Movement (MWM)	16	1.3
			j. Standing MWM	16	1.3
			k. Mobilisation into functional position	14	1.2

For peer review only - http://bmjopen.bmj.com/site/about/guidelines.xhtml

			l. Muscle stretches	61	5.1
			m. Functional movement re-education	7	0.6
**Understanding** [40]	66 (96%)		a. Management of erroneous believes relating to LRS provide education to help eradicate these beliefs	57	4.7
			b. Pacing behaviours	53	4.4
			c. Goal attainment	58	4.8
			d. Health Promotion	80	6.6
			e. Identification and treatment of central sensitisation-liaison with G.P/pain clinic	8	0.7
			f. Identification and treatment of peripheral sensitisation-liaison with G.P/pain clinic	7	0.6
**Conditioning** [41,42]	63 (91%)	Self-assessment answers, clinical interview & history	a. Cardiovascular & conditioning exercise relevant to patients' goals	83	6.9
			b. Function specific stretches	39	3.2
			c. Function specific strengthening	62	5.2
			d. Ergonomic advice	14	1.2
			e. Ergonomic practise	6	0.5
			f. Group exercise	0	0.0
			g. Perturbation training	7	0.6
**Movement control** [43]	33 (48%)	Clinical assessment	a. Sagittal plane control in functional positions relevant to patients' problems/goals	24	2.0
			b. Coronal plane control in functional positions relevant to patients' problems/goals	15	1.2
			c. Axial plane control in functional positions relevant to patients' problems/goals	1	0.1
			d. Multi-planar control in functional positions relevant to patients' problems/goals	6	0.5
			e. Movement re-education in functional positions relevant to patients' problems/goals	18	1.5
**Pain** [44–46]	52 (75%)	ODI VAS back & leg Clinical interview & history	a. Analgesic review & advice in liaison with G.P/Pharmacist	23	1.9
			b. Pain education	60	5.0
			c. Pain coping strategies	20	1.7
			d. Fear reduction intervention in liaison with psychologist/pain clinic	12	1.0
			e. Stress reduction intervention in liaison with psychologist/pain clinic	32	2.7
**Totals**				1267	99.8%[1]

[1] 0.2% missing data-2 treatment episodes where components not attributed.

For peer review only - http://bmjopen.bmj.com/site/about/guidelines.xhtml

Table 4 Sample sizes for main RCT for a range of target mean differences with a primary outcome of the ODI score at 26-weeks post-randomisation

Significance Level	Power	SD	Target Mean Difference	Standardised Effect Size	Number in each group	Total Sample Size (N)	Total sample size Dropout 15%	Total sample size Dropout 20%
5%	90%	16	2	0.13	1346	2692	3168	3366
5%	90%	16	2.5	0.16	862	1724	2030	2156
5%	90%	16	3	0.19	599	1198	1410	1498
5%	90%	16	3.5	0.22	441	882	1038	1104
5%	90%	16	4	0.25	338	676	796	846
5%	90%	16	4.5	0.28	267	534	630	668
5%	90%	16	5	0.31	217	434	512	544
5%	90%	16	5.5	0.34	179	358	422	448
5%	90%	16	6	0.38	151	302	356	378
5%	90%	16	6.5	0.41	129	258	304	324
5%	90%	16	7	0.44	111	222	262	278
5%	90%	16	7.5	0.47	97	194	230	244
5%	90%	16	8	0.50	86	172	204	216
5%	90%	16	8.5	0.53	76	152	180	190
5%	90%	16	9	0.56	68	136	160	170
5%	90%	16	9.5	0.59	61	122	144	154
5%	90%	16	10	0.63	55	110	130	138

For peer review only

Discussion

This pilot study is the first to explore the role of early intervention physiotherapy for LRS. The study aimed to determine the feasibility of carrying out a full-scale RCT to determine the effectiveness of early physiotherapy for LRS. All of the feasibility parameters were found to be acceptable, including the set-up of G.P centres to recruit participants, recruitment of participants and the retention of 85% of participants at 26 weeks. Both groups received the intervention at the appropriate time, within 2 weeks of randomisation for the early intervention physiotherapy group and after 6 weeks for the usual care group. The acceptance of the intervention, judged by the rate of attendance by participants at their treatment sessions, was better than anticipated.

There were some limitations to this study. Firstly, although recruitment was satisfactory and ahead of time, the G.Ps involved in the study were well motivated and supportive of the study, in a city with a proven track-record of G.P involvement in service development and research. This may not be the case across the country and further afield. Similarly, the support of the service provider clinical, administrative and management staff was a key factor in the success of the study, a factor which may not be reproducible in other centres. Patients self-referred into the study after an introduction from their G.P (a pre-requisite for ethics approval) and so this group of patients may not be representative of a wider population. These factors need to be taken in account when planning a definitive study, and we have taken a more conservative view of attrition in the definitive sample size calculation. Our recommendations about recruitment also suggest including a wider geographical spread of G.P centres to help meet the proposed recruitment rates. Site selection would need to consider current service provision and the ability to deliver the intervention in settings that are convenient and accessible to patients. The reliance on a clinical diagnosis of LRS made by the G.P and physiotherapists is a potential limitation. The limitation being that there is likely to be a degree of diagnostic heterogeneity within the sample using a patho-anatomical model of care. There is therefore potential that participants with LRS in the study may have symptoms from something other than nerve root inflammation, including pseud-radicular symptoms, somatic or visceral referred symptoms.

The strengths of the study are that it was a pragmatic study in a clinical setting, using clinical staff and available resources and as such represents the real world of the NHS. We demonstrated that the study is feasible and the potential of early intervention physiotherapy to improve patient care.

For peer review only - http://bmjopen.bmj.com/site/about/guidelines.xhtml

Conclusion

The POLAR study results indicate that a full-scale trial of early physiotherapy to treat patients with LRS is feasible. As there is a dearth of evidence about how and when best to treat this population, we conclude that a definitive trial is needed to help inform clinical practice.

Other information

Ethical review

Ethical approval was received from NHS Scotland, East of Scotland Research Ethics Service (EoSRES) in August 2015 (REC reference 15/ES/0130). The study was conducted in accordance with the declaration of Helsinki and local governance requirements.

Trial Registration

ISRCTN: 25018352

Clinical Trials.Gov: NCT02618278

Acknowledgments

The authors would like to thank the members of the Patient, Public Involvement and Engagement (PPIE) group (Paul Colton, Amanda Dempsey, Rachel Harrison and Claire Village) for their invaluable support and guidance. Thank-you to Dr Jon Dickson, Mark Rhymes and Mark Pinkerton of the Yorkshire and Humber CRN. Thank-you to the wonderful local physiotherapy service for their support, especially the fantastic physiotherapists who delivered the intervention and management (Sarah Withers & Helen Wilson). Finally, and most importantly a massive thank-you to the participants.

Contributors

MR – Instigated the idea for the study, developed the funding proposal and applied for funding. He developed the protocol, intervention handbook, gained ethical approval and acted as CI for the study.

SW – Is the primary PhD supervisor for MRs' fellowship and contributed to the study conception, design, and writing of the protocol and provided guidance with the statistical analysis.

JC – Is an academic supervisor for MR and has provided specific guidance on protocol development, regulatory approvals and the design of the study.

For peer review only

SB – Provided input regarding the qualitative and mixed method design and analysis aspects of the study.

AAC- Provides clinical supervision for M.R. He has been involved in the conception of the study, its organisation, analysis and writing.

All authors read and commented on drafts and approved the final version of the manuscript.

Competing interests

MR–None

SW-None

JC-None

SB-None

AAC-None

All authors have completed the Unified Competing Interest form at www.icmje.org/coi_disclosure.pdf (available on request from the corresponding author) and declare (1) No financial support for the submitted work from anyone other than their employer; (2) No financial relationships with commercial entities that might have an interest in the submitted work; (3) No spouses, partners, or children with relationships with commercial entities that might have an interest in the submitted work; (4) No non-financial interests that may be relevant to the submitted work.

Funding

The lead author (MR) has received a personal Clinical Doctoral Research Fellowship (CDRF) award from Health Education England (HEE) and the National Institute of Health Research (NIHR). Award number: CDRF-2014-05-046.

Disclaimer

This paper presents independent research funded by the National Institute for Health Research (NIHR). The views expressed are those of the author(s) and not necessarily those of the NHS, the NIHR or the Department of Health

Data sharing statement

For peer review only

There are no additional data available for the study which has not been published. All data is available to anyone interested by contacting the corresponding author.

For peer review only

References

1. Koes, Æ Bart W, van Tulder MW PW. The diagnosis and treatment of sciatica. Br Med J. 2007;334:1313–7.
2. Waddell G. The Back Pain Revolution. 2nd editio. Churchill Livingstone, Edinburgh; 2004.
3. Hoy D, March L, Brooks P, Blyth F, Woolf A, Bain C, et al. The global burden of low back pain: estimates from the Global Burden of Disease 2010 study. Ann Rheum Dis [Internet]. 2014;73(6):968–74.
4. Konstantinou K, Dunn KM. Sciatica Review of Epidemiological Studies and Prevalence Estimates. Spine (Phila Pa 1976). 2008;33(22):2464–72.
5. NICE. Low back pain and sciatica in over 16s: assessment and management [Internet]. Https://Www.Nice.Org.Uk/Guidance/Ng59. 2016.
6. Vroomen PC a J, de Krom MCTFM, Knottnerus J a. Predicting the outcome of sciatica at short-term follow-up. Br J Gen Pract [Internet]. 2002 Feb;52(475):119–23.
7. Suri P, Carlson MJ, Rainville J. Nonoperative Treatment for Lumbosacral Radiculopathy: What Factors Predict Treatment Failure? Clin Orthop Relat Res [Internet]. Springer US; 2014;1931–9.
8. Thomas KC, Fisher CG, Boyd M, Bishop P, Wing P, Dvorak MF. Outcome Evaluation of Surgical and Nonsurgical Management of Lumbar Disc Protrusion Causing Radiculopathy. Spine (Phila Pa 1976). 2007;32(13):1414–22.
9. Lau PM-Y, Chow DH-K, Pope MH. Early physiotherapy intervention in an Accident and Emergency Department reduces pain and improves satisfaction for patients with acute low back pain: a randomised trial. Aust J Physiother [Internet]. 2008 Jan;54(4):243–9.
10. Wand BM, Bird C, Mcauley JH, Dore CJ, Macdowell M, Souza LH De. Early Intervention for the Management of Acute Low Back Pain Education , Manual Therapy , and Exercise. Spine (Phila Pa 1976). 2004;29(21):2350–6.
11. Whitfill T, Haggard R, Bierner SM, Pransky G, Hassett RG, Gatchel RJ. Early intervention options for acute low back pain patients: A randomized clinical trial with one-year follow-up outcomes. J Occup Rehabil. 2010;20(2):256–63.
12. Fritz JM, Childs JD, Wainner RS, Flynn TW. Primary care referral of patients with low back pain to physical therapy: impact on future health care utilization and costs. Spine (Phila Pa 1976) [Internet]. 2012 Dec 1 [cited 2014 Jan 31];37(25):2114–21.
13. Gellhorn AC, Chan L, Martin B, Friedly J. Management patterns in acute low back pain: the role of physical therapy. Spine (Phila Pa 1976) [Internet]. 2012 Apr 20 [cited 2014 Feb 3];37(9):775–82.
14. Rihn JA, Hilibrand AS, Radcliff K, Kurd M, Lurie J, Blood E, et al. Duration of Symptoms Resulting from Lumbar Disc Herniation: Effect on Treatment Outcomes. J Bone Jt Surg [Internet]. 2011;93–A(20):1906–14.
15. Weinstein JN, Lurie JD, Tosteson TD, Tosteson AN a, Blood E a, Abdu W a, et al. Surgical versus nonoperative treatment for lumbar disc herniation: four-year results for the Spine Patient Outcomes Research Trial (SPORT). Spine (Phila Pa 1976) [Internet]. 2008 Dec;33(25):2789–800.
16. Jacobs WCH, van Tulder M, Arts M, Rubinstein SM, van Middelkoop M, Ostelo R, et al. Surgery versus conservative management of sciatica due to a lumbar herniated disc: a systematic review. Eur Spine J [Internet]. 2011;20(4):513–22.
17. Peul WC, Arts MP, Brand R, Koes BW. Timing of surgery for sciatica: subgroup analysis alongside a randomized trial. Eur Spine J [Internet]. 2009;18(4):538–45.
18. Peul WC, Brand R, Thomeer RTWM, Koes BW. Improving prediction of “inevitable” surgery during non-surgical treatment of sciatica. Pain. 2008;138(3):571–6.
19. Alentado VJ, Mroz TE, Lubelski D, Steinmetz MP, Benzel EC. Optimal Duration of Conservative Management Prior to Surgery for Cervical and Lumbar Radiculopathy : A Literature Review. Glob Spine J. 2014;279–86.
20. Machado GC., Witzleb AJ., Fritsch C., Maher CG., Ferreira PH., Ferreira ML. d. Patients with sciatica still experience pain and disability 5 years after surgery: A systematic review with meta-analysis of cohort studies. Eur J Pain (United Kingdom) [Internet]. 2016;20(March):1–8.
21. Lequin MB, Verbaan D, Jacobs WCH, Brand R, Bouma GJ, Vandertop WP, et al. Surgery versus prolonged conservative treatment for sciatica: 5-year results of a randomised controlled trial. BMJ Open [Internet]. 2013 Jan [cited 2013 Jul 15];3(5):1–10.
22. Reddington M, Walters SJ, Cohen J, Baxter S. Does early intervention improve outcomes in physiotherapy management of lumbar radicular syndrome? A mixed-methods study protocol.

For peer review only - http://bmjopen.bmj.com/site/about/guidelines.xhtml

BMJ Open [Internet]. 2017;7(3):e014422.
23. Fairbank JC, Pynsent PB. The Oswestry Disability Index. Spine (Phila Pa 1976) [Internet]. 2000 Nov 15;25(22):2940–52; discussion 2952.
24. Fairbank, JC. Couper, J. Davies, JB. O'Brien J. The Oswestry low back pain disability questionnaire. Physiother. 1980;66(8):271–3.
25. Hahne AJ, Ford JJ, Hinman RS, Richards MC, Surkitt LD, Chan AYP, et al. Individualized functional restoration as an adjunct to advice for lumbar disc herniation with associated radiculopathy. A preplanned subgroup analysis of a randomized controlled trial. Spine J. Elsevier Inc.; 2017;17(3):346–59.
26. Hahne AJ, Ford JJ, Surkitt LD, Richards MC, Chan AYP, Slater SL, et al. Individualized Physical Therapy Is Cost-Effective Compared With Guideline-Based Advice for People With Low Back Disorders. Spine (Phila Pa 1976) [Internet]. 2017;42(3):E169–76.
27. Hill JC, Dunn KM, Lewis M, Mullis R, Main CJ, Foster NE, et al. A primary care back pain screening tool: identifying patient subgroups for initial treatment. Arthritis Rheum [Internet]. 2008;59(5):632–41.
28. EuroQol Group. EuroQol--a new facility for the measurement of health-related quality of life. Health Policy [Internet]. 1990;16(3):199–208.
29. Teare MD, Dimairo M, Shephard N, Hayman A, Whitehead A, Walters SJ. Sample size requirements to estimate key design parameters from external pilot randomised controlled trials: a simulation study. Trials [Internet]. 2014;15(1):264.
30. Fritz JM, Irrgang J.J. A Comparison of a Modified Oswestry Low Back Pain Disability Questionnaire and the Quebec Back Pain Disability Scale. Phys Ther. 2001;81(2):776–88.
31. Ostelo RWJG, Deyo RA, Stratford P, Waddell G, Croft P, Korff M Von, et al. Interpreting Change Scores for Pain and Functional Status in Low Back Pain. Spine (Phila Pa 1976). 2008;33(1):90–4.
32. Nicholas MK, George SZ. Psychologically informed interventions for low back pain: an update for physical therapists. Phys Ther [Internet]. 2011 May [cited 2012 Nov 22];91(5):765–76.
33. Blyth FM, Macfarlane GJ, Nicholas MK. The contribution of psychosocial factors to the development of chronic pain: the key to better outcomes for patients? Pain [Internet]. 2007;129(1–2):8–11.
34. Linton SJ, Shaw WS. Impact of psychological factors in the experience of pain. Phys Ther [Internet]. 2011 May [cited 2012 Oct 28];91(5):700–11.
35. Coppieters MW, Butler DS. Do "sliders" slide and "tensioners" tension? An analysis of neurodynamic techniques and considerations regarding their application. Man Ther. 2008;13(3):213–21.
36. Basson A, Olivier B, Ellis R, Coppieters M, Stewart A, Mudzi W. The Effectiveness of Neural Mobilization for Neuro-Musculoskeletal Conditions: A Systematic Review and Meta-Analysis. J Orthop Sport Phys Ther [Internet]. 2017;47(1401):1–76.
37. Efstathiou MA, Stefanakis M, Savva C, Giakas G. Effectiveness of neural mobilization inpatients with spinal radiculopathy: Acritical review. J Bodyw Mov Ther [Internet]. Elsevier Ltd; 2015;19(2):205–12.
38. Butler D. The Sensitive Nervous System. 1st ed. NOI Group Publications; 2000.
39. Hengeveld E BK. Maitlands' Vertebral Manipulation. 8th ed. Churchill Livingstone, Edinburgh; 2013.
40. Otoo, S.K.W, Hendrick, P, Ribeiro D. The comparative effectiveness of advice/education compared to active physiotherapy (manual therapy and exercise) in the management of chronic non-specific low back pain. Phys Ther Rev. 2015;20(1):16–26.
41. Searle A., Spink M, Ho A, Chuter V. Exercise interventions for the treatment of chronic low back pain: A systematic review and meta-analysis of randomised controlled trials. Clin Rehabil [Internet]. 2015;12:1155–67.
42. Falla D, Whiteley R, Marco C, Hodges P. Therapeutic Exercise. In: Jull G, Moore A, Falla DL, Lewis J, McCarthy C, Sterling M, editors. Grieves Modern Musculoskeletal Physiotherapy. 4th ed. 2015. p. 298–310.
43. O'Sullivan P. Diagnosis and classification of chronic low back pain disorders: maladaptive movement and motor control impairments as underlying mechanism. Man Ther [Internet]. 2005;10(4):242–55.
44. Lorig KR, Holman HR, Med AB. Self-Management Education : History , Definition , Outcomes , and Mechanisms. Ann Behav Med. 2003;26(1):1–7.
45. Pinto RZ, Maher CG, Ferreira ML, Ferreira PH, Hancock M, Oliveira VC, et al. Drugs for relief of pain in patients with sciatica: systematic review and meta-analysis. Bmj [Internet]. 2012 Feb

For peer review only

13 [cited 2012 Mar 8];344(feb13 1):e497–e497.
46. Clarke CL, Ryan CG, Martin DJ. Pain neurophysiology education for the management of individuals with chronic low back pain: A systematic review and meta-analysis. Man Ther [Internet]. Elsevier Ltd; 2011;16(6):544–9.

Figure 1 POLAR CONSORT flowchart

Figure 2 Leg pain and ODI scores across groups

For peer review only

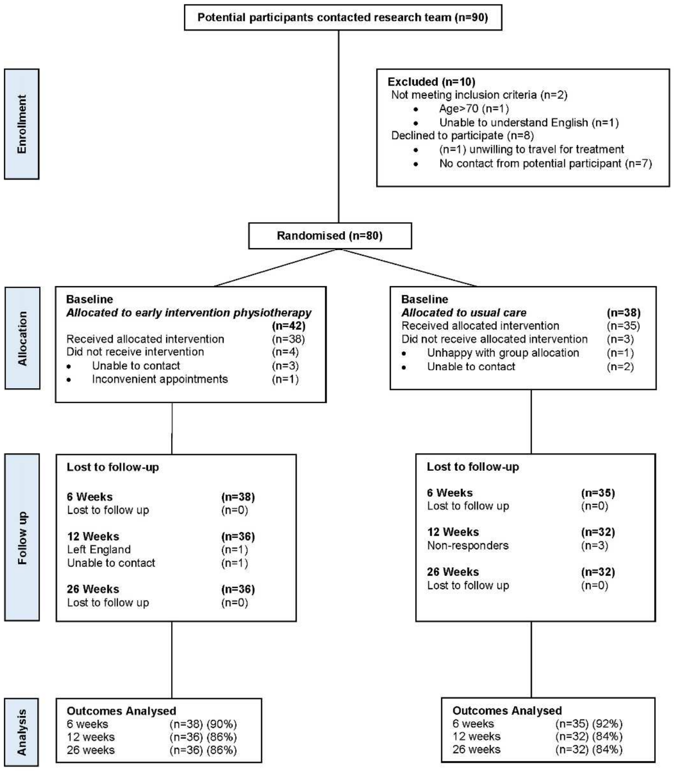

Figure 1 POLAR CONSORT flowchart

209x297mm (300 x 300 DPI)

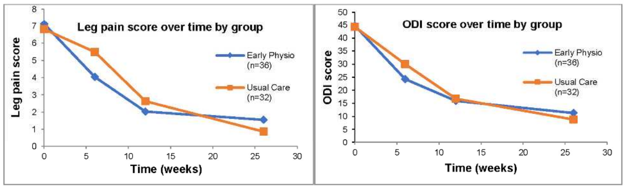

Figure 2 Leg pain and ODI scores across groups

209x297mm (300 x 300 DPI)

POLAR
Physiotherapy for Sciatica Study

PhysiOtherapy Management of LumbAr Radicular Syndrome

Does Early Intervention Improve Outcomes

Patient Recruitment Flow

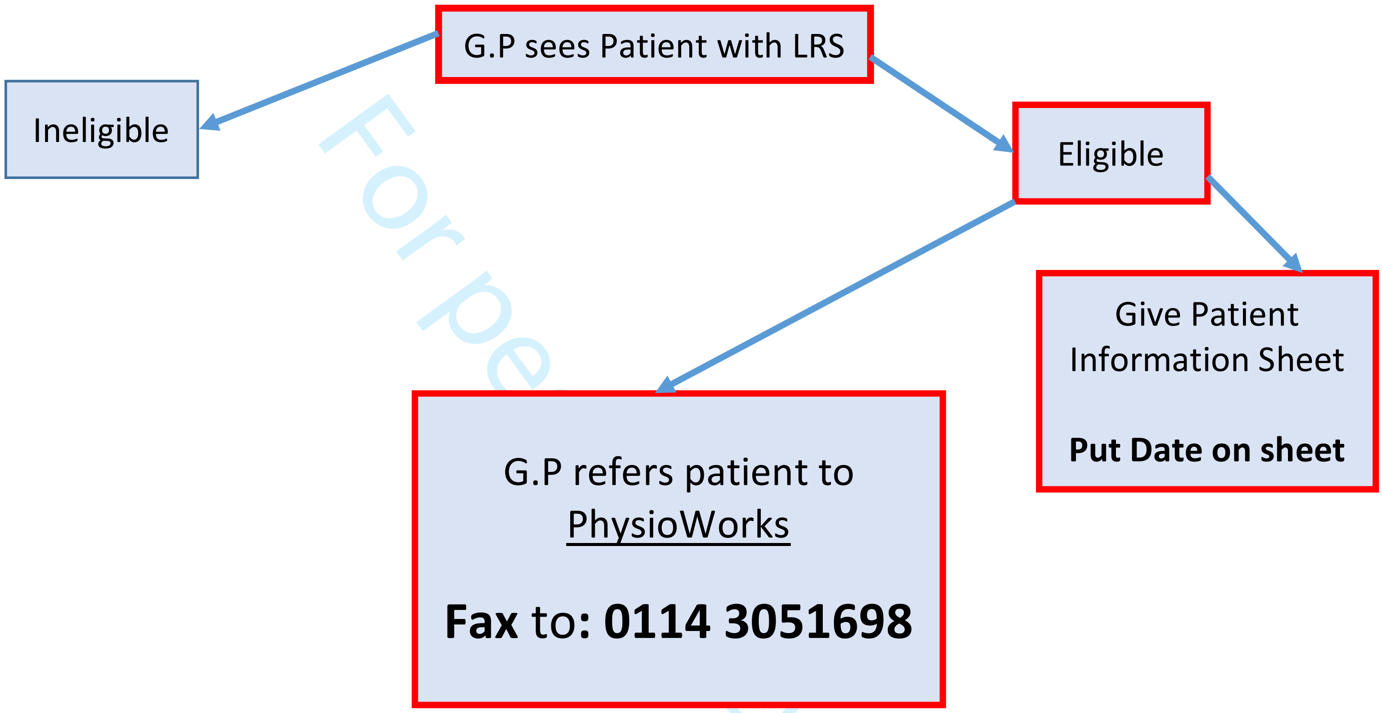

Eligibility Criteria

Must be & have:

1. Unilateral symptoms of **sciatica** or **femoral** radicular pain

   (See over for definitions)

Exclusion Criteria

Must **not** have:

1. 'Red flag' signs or symptoms
2. Cancer at the time of the study
3. Proven vascular claudication
4. Bilateral signs/symptoms
5. Spinal fracture within the last 3 months
6. Poor English skills (necessitating the use of an interpreter)

For peer review only - http://bmjopen.bmj.com/site/about/guidelines.xhtml

Characteristics of Sciatica (L4,5,S1,S2)

- Pain and/or paraesthesia and/or numbness in a L4-S2 dermatomal distribution.
- Symptoms usually buttock, posterior or lateral thigh and anterior/posterior/lateral leg.
- Shooting, burning or lancinating type pain.
- Symptoms extend below the knee.

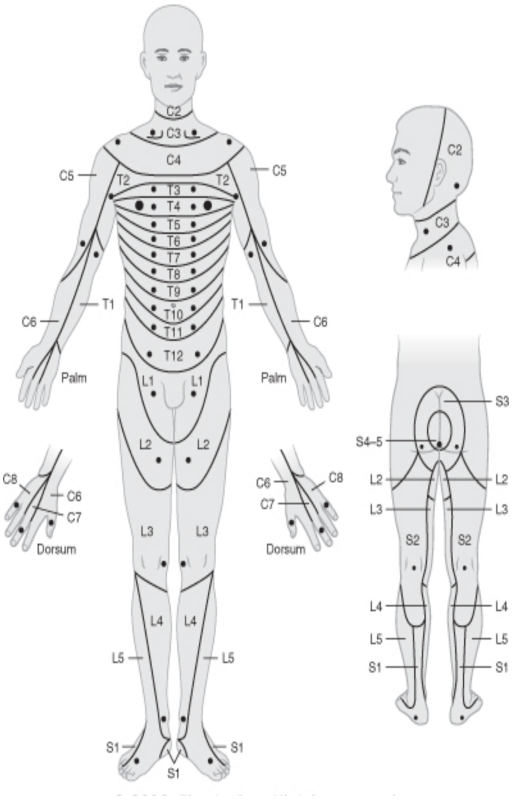

Characteristics of Femoral radicular pain (L1,2,3)

- Anterior/Antero-lateral thigh pain and/or paraesthesia and/or numbness in L1-3 dermatomal distribution.
- Shooting, burning or lancinating type pain.
- Symptoms extend above or to the knee, not below the knee.

Red Flag Signs and Symptoms

Red flag signs or symptoms are those which arose suspicion of potential serious or life-threatening pathology. There are hundreds of red flags in the literature, most of which of spurious validity. However a few useful ones in relation to low back pain and sciatica are:

- Previous cancer
- Systemically unwell (? Potential discitis)
- Spinal deformity (? fracture)
- Significant, unexplained weight loss
- Severe, unremitting pain
- I.V drug use (infection-?discitis)
- Cauda equina syndrome
- Upper motor neurone signs or symptoms (loss of dexterity, worsening balance, gait disturbance, falls, positive UMN tests-hyperreflexia, positive Romberg sign, positive Hoffman sign, muscular hypertonicity etc)

This list is of course not exhaustive and services only to highlight those patients who require further investigation rather than physiotherapy.

For peer review only - http://bmjopen.bmj.com/site/about/guidelines.xhtml

For peer review only

Does early intervention improve outcomes in the physiotherapy management of Lumbar Radicular Syndrome?
Logic Model

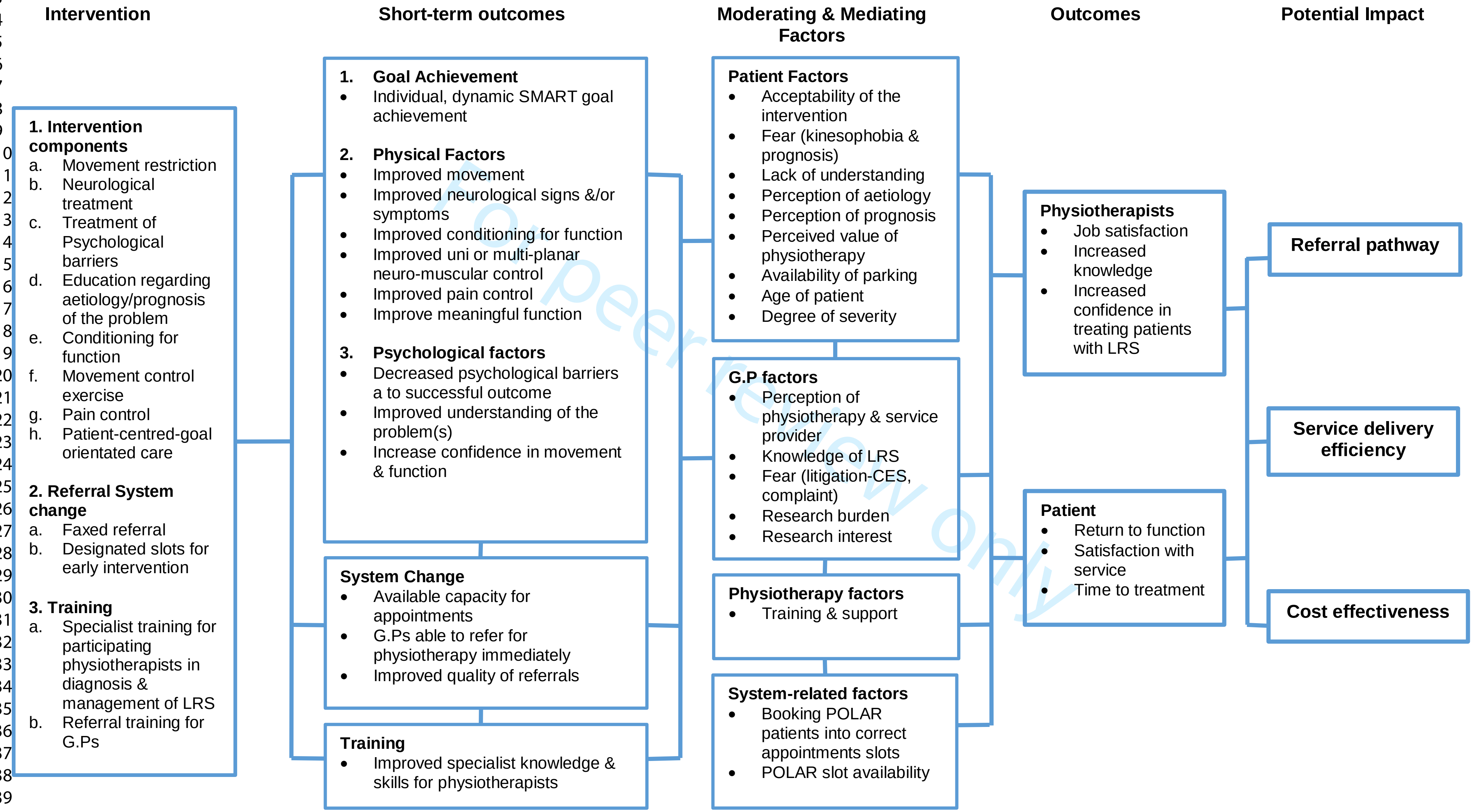

For peer review only

Does early intervention improve outcomes in the physiotherapy management of Lumbar Radicular Syndrome? Logic Model V1 29.6.16

POLAR CONSORT 2010 checklist of information to include when reporting a pilot or feasibility trial*

Section/Topic	Item No	Checklist item	Reported on page No
**Title and abstract**			
	1a	Identification as a pilot or feasibility randomised trial in the title	1
	1b	Structured summary of pilot trial design, methods, results, and conclusions (for specific guidance see CONSORT abstract extension for pilot trials)	2,3
**Introduction**			
Background and objectives	2a	Scientific background and explanation of rationale for future definitive trial, and reasons for randomised pilot trial	4
	2b	Specific objectives or research questions for pilot trial	5
**Methods**			
Trial design	3a	Description of pilot trial design (such as parallel, factorial) including allocation ratio	5,6
	3b	Important changes to methods after pilot trial commencement (such as eligibility criteria), with reasons	5
Participants	4a	Eligibility criteria for participants	6
	4b	Settings and locations where the data were collected	5
	4c	How participants were identified and consented	6
Interventions	5	The interventions for each group with sufficient details to allow replication, including how and when they were actually administered	7
Outcomes	6a	Completely defined prespecified assessments or measurements to address each pilot trial objective specified in 2b, including how and when they were assessed	7, 8
	6b	Any changes to pilot trial assessments or measurements after the pilot trial commenced, with reasons	N/A
	6c	If applicable, prespecified criteria used to judge whether, or how, to proceed with future definitive trial	5
Sample size	7a	Rationale for numbers in the pilot trial	8
	7b	When applicable, explanation of any interim analyses and stopping guidelines	N/A
Randomisation:			
Sequence generation	8a	Method used to generate the random allocation sequence	6
	8b	Type of randomisation(s); details of any restriction (such as blocking and block size)	6
Allocation concealment mechanism	9	Mechanism used to implement the random allocation sequence (such as sequentially numbered containers), describing any steps taken to conceal the sequence until interventions were assigned	6

For peer review only

Implementation	10	Who generated the random allocation sequence, who enrolled participants, and who assigned participants to interventions	6
Blinding	11a	If done, who was blinded after assignment to interventions (for example, participants, care providers, those assessing outcomes) and how	N/A
	11b	If relevant, description of the similarity of interventions	7
Statistical methods	12	Methods used to address each pilot trial objective whether qualitative or quantitative	8
**Results**			
Participant flow (a diagram is strongly recommended)	13a	For each group, the numbers of participants who were approached and/or assessed for eligibility, randomly assigned, received intended treatment, and were assessed for each objective	9
	13b	For each group, losses and exclusions after randomisation, together with reasons	9
Recruitment	14a	Dates defining the periods of recruitment and follow-up	10
	14b	Why the pilot trial ended or was stopped	10
Baseline data	15	A table showing baseline demographic and clinical characteristics for each group	10
Numbers analysed	16	For each objective, number of participants (denominator) included in each analysis. If relevant, these numbers should be by randomised group	10,11,12
Outcomes and estimation	17	For each objective, results including expressions of uncertainty (such as 95% confidence interval) for any estimates. If relevant, these results should be by randomised group	11,12
Ancillary analyses	18	Results of any other analyses performed that could be used to inform the future definitive trial	
Harms	19	All important harms or unintended effects in each group (for specific guidance see CONSORT for harms)	12,13
	19a	If relevant, other important unintended consequences	N/A
**Discussion**			
Limitations	20	Pilot trial limitations, addressing sources of potential bias and remaining uncertainty about feasibility	18
Generalisability	21	Generalisability (applicability) of pilot trial methods and findings to future definitive trial and other studies	18
Interpretation	22	Interpretation consistent with pilot trial objectives and findings, balancing potential benefits and harms, and considering other relevant evidence	18
	22a	Implications for progression from pilot to future definitive trial, including any proposed amendments	18, 19
**Other information**			
Registration	23	Registration number for pilot trial and name of trial registry	19
Protocol	24	Where the pilot trial protocol can be accessed, if available	3
Funding	25	Sources of funding and other support (such as supply of drugs), role of funders	20
	26	Ethical approval or approval by research review committee, confirmed with reference number	19

For peer review only

Citation: Eldridge SM, Chan CL, Campbell MJ, Bond CM, Hopewell S, Thabane L, et al. CONSORT 2010 statement: extension to randomised pilot and feasibility trials. BMJ. 2016;355.
*We strongly recommend reading this statement in conjunction with the CONSORT 2010, extension to randomised pilot and feasibility trials, Explanation and Elaboration for important clarifications on all the items. If relevant, we also recommend reading CONSORT extensions for cluster randomised trials, non-inferiority and equivalence trials, non-pharmacological treatments, herbal interventions, and pragmatic trials. Additional extensions are forthcoming: for those and for up to date references relevant to this checklist, see www.consort-statement.org.

For peer review only
